# Tetrandrine Citrate Suppresses Breast Cancer *via* Depletion of Glutathione Peroxidase 4 and Activation of Nuclear Receptor Coactivator 4-Mediated Ferritinophagy

**DOI:** 10.3389/fphar.2022.820593

**Published:** 2022-05-09

**Authors:** Jiameng Yin, Yajun Lin, Weiwei Fang, Xin Zhang, Jie Wei, Gang Hu, Pu Liu, Jie Niu, Jun Guo, Yongzhan Zhen, Jian Li

**Affiliations:** ^1^ Hebei Key Laboratory for Chronic Diseases, School of Basic Medical Sciences, North China University of Science and Technology, Tangshan, China; ^2^ The Key Laboratory of Geriatrics, Beijing Institute of Geriatrics, Institue of Geriatric Medicine, Chinese Academy of Medical Sciences, Beijing Hospital/National Center of Gerontology of National Health Commission, Beijing, China; ^3^ State Key Laboratory of Molecular Oncology, National Cancer Center/National Clinical Research Center for Cancer/Cancer Hospital, Department of Blood Transfusion, Chinese Academy of Medical Sciences and Peking Union Medical College, Beijing, China

**Keywords:** tetrandrine citrate, breast cancer, ferroptosis, glutathione peroxidase 4, nuclear receptor coactivator 4

## Abstract

Tetrandrine citrate (TetC), a novel tetrandrine salt with high water solubility, demonstrates a potent antitumor activity in chronic myeloid leukemia. Studies have indicated an important role of ferroptosis in breast cancer (BC). However, whether TetC inhibits BC progression *via* ferroptosis has never been explored. In the present study, we showed that TetC had a significant inhibitory effect on the proliferation and migration of MCF7 and MDA-MB-231 cells. Then, we combined TetC with different inhibitors to determine which form of cell death could be driven by TetC. MTT assay showed that ferrostatin (Fer-1) demonstrated the most potent effect on improving TetC-induced cell death in contrast to other inhibitors. TetC was also shown to significantly increase the mRNA level of prostaglandin-endoperoxide synthase 2 (Ptgs2), a ferroptosis marker. Further studies showed that TetC significantly suppressed the expression of glutathione peroxidase 4 (GPX4) and ferritin heavy chain 1 (FTH1) but increased the expression of nuclear receptor coactivator 4 (NCOA4) in MCF7 and MDA-MB-231 cells even in the presence of erastin or Ras-selective lethal 3 (RSL3). Collectively, we showed novel data that ferroptosis was a major form of TetC-induced cell death. Moreover, TetC-induced ferroptotic cell death was achieved *via* suppressing GPX4 expression and activating NCOA4-mediated ferritinophagy in BC cells.

## Introduction

Breast cancer (BC) is one of the most frequently occurring malignancies and ranks second among causes for cancer-related death in women (Harbeck and Gnant, 2017). However, metastasis of vital organs is considered to be the main cause of BC-related deaths (Liang et al., 2020). Although the treatment approaches have been improved in the past years, BC patients still suffer from therapeutic failures due to the increase in chemotherapy or endocrine therapy resistance (Waks and Winer, 2019). Therefore, it is imperative to develop novel therapeutic means to clear tumor cells.

Ferroptosis is a regulated form of necrotic cell death that is accompanied by intracellular iron-dependent oxidative damage of lipids (Yang and Stockwell, 2016). The canonical pathway of ferroptosis is medicated by inactivating the activity or depletion of glutathione peroxidase 4 (GPX4), a lipid peroxide scavenger, which protects membrane liquid from ferroptosis *via* taking glutathione (GSH) as a cofactor (Li Z. et al., 2020). Erastin is an inhibitor of solute carrier family seven member 11 (SLC7A11, also commonly known as xCT), which results in a decreased intracellular GSH level, while Ras-selective lethal 3 (RSL3) directly represses GPX4 activity (Sui et al., 2018). The noncanonical ferroptosis is initiated by elevating the labile iron pool (LIP) (Hassannia et al., 2019). Specifically, nuclear receptor coactivator 4 (NCOA4)-mediated degradation of ferritin heavy chain 1 (FTH1) leads to an increase in iron, thus resulting in ferritinophagy-dependent cell death (Gao et al., 2016; Hassannia et al., 2019; Park and Chung, 2019). Accumulating evidence suggests that ferroptotic cell death plays a key role in inhibiting BC cell growth, and targeting ferroptosis may be of great potential for anticancer therapy in patients with BC (Li Z et al., 2020; Sun et al., 2021).

Tetrandrine (Tet) is a natural product that has been extensively applied for the therapy of lung fibrosis and arthritis in China (Chen, 2002; Su et al., 2020). The antitumor activity of Tet has been reported in chronic myeloid leukemia and human laryngeal cancer (Xu et al., 2012; Li Y et al., 2020), but its application in the clinical field is limited since Tet is a hydrophobic alkaloid with poor solubility in water. Tetrandrine citrate (TetC), a novel tetrandrine salt with high water solubility, demonstrates a potent antitumor activity in chronic myeloid leukemia (Xu et al., 2012). Previous studies have indicated that Tet suppresses tumor growth *via* inducing apoptosis and autophagy in BC (Xu et al., 2011; Liang and Pei, 2017; Wong et al., 2017). However, whether TetC inhibits BC progression *via* ferroptosis has never been explored.

In the present study, we explored the relationship between TetC and ferroptosis in BC cells. For the first time, we demonstrated that TetC induced feroptosis through inhibiting GPX4 and activating ferritinophagy in BC cells.

## Materials and Methods

### Reagents and Chemicals

Thiazolyl blue tetrazolium bromide (MTT) and dimethyl sulfoxide (DMSO) were purchased from SigmaAldrich (Darmstadt, Germany), and tetrandrine (98% purity) was purchased from Nanjing Kisaisi Medical Technology (Nanjing, China). All of the kits used to measure indicators of oxidative stress (including malondialdehyde [MDA], SOD, and GSH-Px) were purchased from Nanjing Jiancheng Institute of Biological Engineering (Nanjing, China). The ferrous ion colorimetric assay kit was purchased from Elabscience. We also purchased a DCFH-DA fluorescent probe from Solarbio (China). The primary antibodies used were anti-NCOA4 (66849T Cell Signaling, Danvers, MA, United States), anti-GPX4 (52455T, Cell Signaling), anti-FTH1 (4393T, Cell Signaling), anti-Atg5 (12994T, Cell Signaling), Atg7 (8558T, Cell Signaling), TfR (13113T, Cell Signaling), LC3A (4599T, Cell Signaling), and anti-*β*-actin (4970T, Cell Signaling). The compounds used were erastin (HY-15763, MedChemExpress, Shanghai, China), RSL3 (HY-100218A, MedChemExpress, Shanghai, China), ferrostatin-1 (HY-100579, MedChemExpress, Shanghai, China), Z-VAD (OMe)-FMK (HY-16658, MedChemExpress, Shanghai, China), 3-methyladenine (3-MA) (HY-19312, MedChemExpress, Shanghai, China), necrostatin-1 (HY-15760, MedChemExpress, Shanghai, China), Chloroquine (CQ) (HY-17589A, MedChemExpress, Shanghai, China), deferiprone (DFO) (HY-B0568, MedChemExpress, Shanghai, China), and acetylcysteine (NAC) (HY-B0215, MedChemExpress, Shanghai, China). Anti-rabbit IgG (cat. no. 7074P2) was purchased from Cell Signaling Technology.

### Cell Lines and Cell Culture

MDA-MB-231 and MCF7 cell lines were obtained from the American Tissue Culture Collection (ATCC) and cultured in a medium recommended by the ATCC. MCF7 cells were cultured in high glucose DMEM supplemented with 10% fetal bovine serum (FBS) and 1% penicillin–streptomycin in 5% CO_2_ at 37°C. MDA-MB-231 cells were cultured using complete RPMI 1640 supplemented with 10% FBS, 1% penicillin, and streptomycin) (Cyclone Utah, United States). Unless otherwise indicated, the cell culture medium was changed every 2 days, and cells were passaged using 0.05% trypsin/EDTA. All cell lines were cultured in a humidified incubator at 37°C under 5% CO_2_.

### Cell Proliferation Assay

A total of 2000 cells per well (MCF7 and MDA-MB-231) were seeded in a 96-well plate and treated with erastin (MedChemExpress, Shanghai, China), RSL3 (MedChemExpress, Shanghai, China), and/or TetC (Kisaisi, Nanjing, China) with or without ferrostatin-1 (Fer-1, inhibitor of erastin-induced ferroptosis; MedChemExpress) for 24 h. MCF7 (2×10^3^ cells/well) and MDA-MB-231 (2×10^3^ cells/well) cells were seeded into a 96-well microplate, cultured at 37°C for 24 h, and treated with 1 µM ferrostatin (Fer-1) 2 h prior to TetC, erastin, and RSL3 treatment. After 24 h, 5 mg/ml 3-(4,5-dimethylimidazole-2-y1)-2,5-diphenyltetrazolium bromide (MTT) solution (20 µl/well) was added and then incubated for 4 h at 37°C in the dark. Finally, the supernatants were removed and 150 µl DMSO was added to dissolve the formazan crystals. The cell viability was determined at a wavelength of 490 nm by a microplate reader (Multiskan™ MK3; Thermo Fisher Scientific, Inc., US). The IC_50_ values were calculated using GraphPad Prism 8.4 software (GraphPad Software, Inc.)

### Determination of Reactive Oxygen Species

Intracellular ROS production was assessed by measuring the fluorescence intensity of 2,7-dichlorodi-hydrofluorescein diacetate (DCFH-DA). MCF7 and MDA-MB-231 were seeded in 6-well plates (1 × 10^5^ cells/well). Then, the cells were treated with 20 µM TetC, 1 µM RSL3, and/or 20 µM erastin for 24 h. After 24 h, the cells were washed three times in phosphate-buffered saline (PBS) and then loaded with a DCFH-DA probe (1:1,000, 20 min) at 37°C in a CO_2_ incubator. After rinsing in PBS, the images were finally acquired by fluorescence microscopy (Olympus, Tokyo, Japan).

### Oxidative Stress Detection

MCF7 and MDA-MB-231 cells were collected and dissolved in 300 ul PBS. The collected cells were then broken by ultrasound on ice. Then, we measured the levels of malondialdehyde (MDA), glutathione (GSH), and Fe^2+^ using the malondialdehyde (MDA) assay kit (TBA method), glutathione peroxidase (GSH-PX) assay kit (colorimetric method), and ferrous ion colorimetric assay kit according to the manufacturer’s instructions.

### Immunoblot Analysis

Total protein extracts were then used in Western blotting with a variety of antibodies, including *β*-actin (1:1,000, CST, US), NCOA4 (1:1,000, CST, US), FTH1 (1:1,000,CST, US), autophagy related 5 (ATG5), autophagy related 7 (ATG7), microtubule-associated protein one light chain 3 (LC3), and transferrin receptor (TfR), GPX4 (1:1,000, CST, US). These proteins were analyzed using enhanced chemiluminescence (ECL) Western blot assay (GE Healthcare, US). ImageJ software version 1.0 was then used to quantify the optical density of each protein band and *β*-actin was used as an internal control.

### Flow Cytometry

A total of 1 × 10^5^ MCF7 and MDA-MB-231 cells were seeded in six-well plates, and then the cells were treated with erastin, RSL3, TetC, and/or Fer-1. The cells were collected and stained with Annexin V-FITC and 7-AAD (AnnexinV-FITC Kit; Baiaolaibo Technology, Beijing), following the manufacturer’s instruction, and analyzed using FlowJo 10 software (Tree Star Inc, Ashland, OR).

### RNA Preparation and Quantitative qRT-PCR

Total cell RNA was extracted using the TRIzol reagent (TIANGEN, Beijing, China). The extracted RNA was used on a One Step TB Green PrimeScript RT-PCR kit (Perfect Real Time) (Takara, Tokyo, Japan) with GAPDH served as an inner control, according to the manufacturer’s instructions.

The primers used in the present study are listed as follows:Ptgs2-F: 5′- CAT​CAA​TGC​AAG​TTC​TTC​CC-3’; Ptgs2-R: 5′- CAG​TCG​AAC​GTT​CTT​TTA​GT-3’; CHAC1-F: 5′- CCA​CTG​AGC​AGA​TAT​GGT​G-3’; CHAC1-R: 5′- ACA​CCA​ACA​TGG​TGC​AAT​AA-3’; GPX4-F: 5′-GAA​CTT​CAC​CAA​GTT​TGG​AC-3’; GPX4-R: 5′-CTT​GTC​GAT​GAG​GAA​CTG​TG-3’; GAPDH-F: 5′- CTC​TGA​CTT​CAA​CAG​CGA​C-3’; GAPDH-R: 5′- CGT​TGT​CAT​ACC​AGG​AAA​TG -3’.

### Statistical Analysis

All statistical analyses were performed using Graphpad Prism 8.4. Data were presented as mean ± standard deviation (SD). The two-tailed unpaired Student’s t-tests were used for comparisons of two groups. The one-way ANOVA multiple comparison test (SPSS 20.0), followed by the Tukey post hoc test, was used to determine the statistical significance of multiple comparisons. *p* < 0.05 was considered statistically significant.

## Results

### MCF7 and MDA-MB-231 Cells are Sensitive to Erastin- and RSL3-Induced Ferroptosis

**FIGURE 1:**
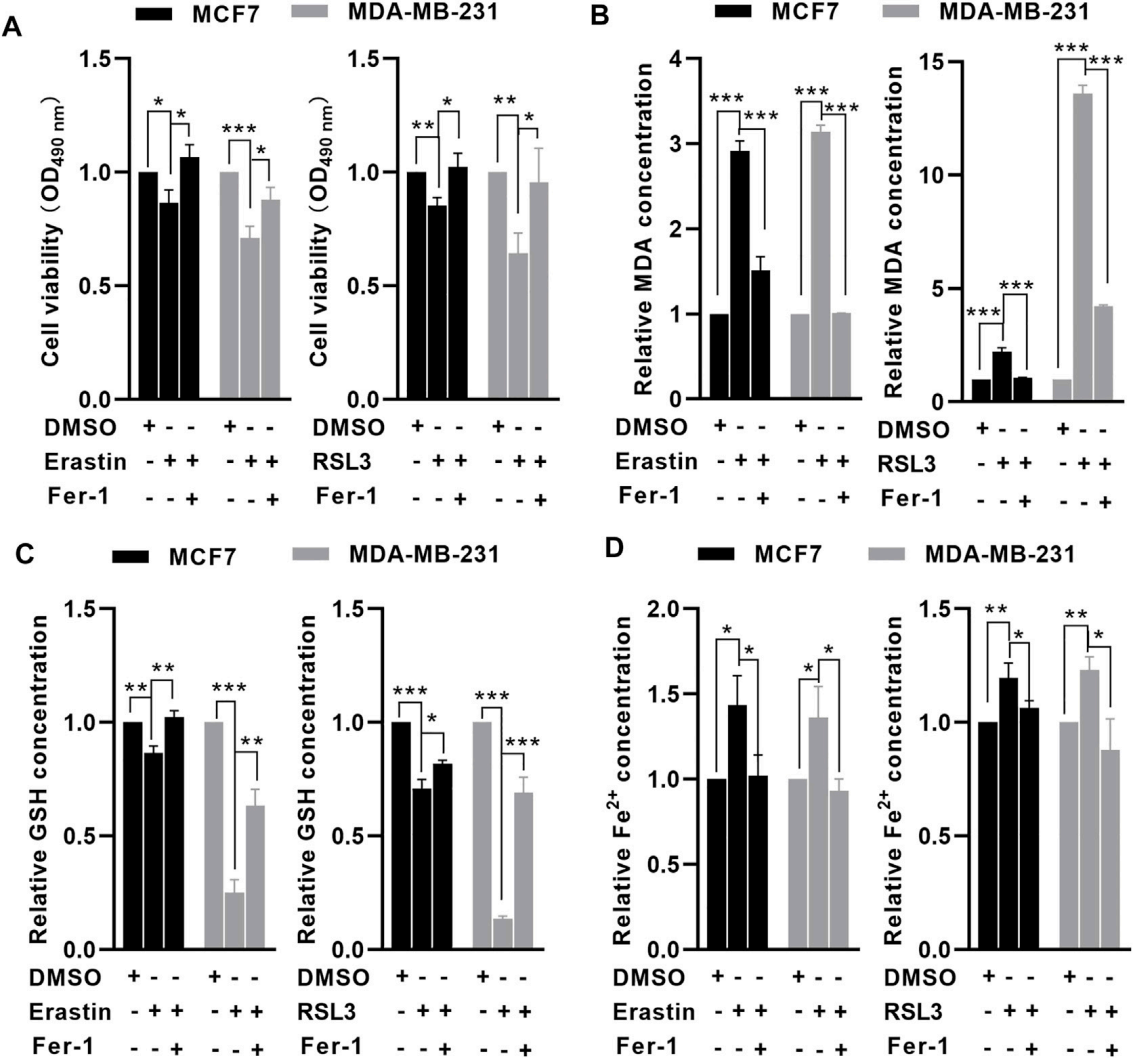
MCF7 and MDA-MB-231 cells were sensitive to erastin- and RSL3-induced ferroptosis. MCF7 and MDA-MB-231 cells were preincubated with or without 1 μM Fer-1 for 1 h. After that, the cells were treated with erastin (10 μM) or RSL3 (1 μM) for 24 h. MTT assay showed that erastin or RSL3 significantly reduced MCF7 and MDA-MB-231 cell viability. **(A)** Erastin or RSL3 reduced GSH **(B)** levels but increased intracellular MDA **(C)** and free iron **(D)** levels in MCF7 and MDA-MB-231 cells (*n* = 3 independent repeats). ^*^ *p* < 0.05; ^**^ *p* < 0.01; ^***^ *p* < 0.001.

To investigate whether MCF7 and MDA-MB-231 cells were sensitive to ferroptosis, an MTT assay was performed to examine the effects of erastin and RSL3 on BC cell viability. Following treatment with 10 μM erastin and 1 μM RSL3 for 24 h, cell viability was significantly reduced in MCF7 and MDA-MB-231 cells compared with untreated cells, but ferrostatin-1 (Fer-1, a ferroptosis inhibitor) could significantly reverse erastin- and RSL3-induced cell death (Figure 1A). Following erastin and RSL3 treatment, the level of GSH was decreased (Figure 1B), while the relative levels of MDA and free iron were increased in MCF7 and MDA-MB-231 cells (Figures 1C,D). In contrast, preincubation with Fer-1 could rescue such effects induced by erastin and RSL3 in BC cells (Figures 1C,D). These data indicated that MCF7 and MDA-MB-231 cells were sensitive to erastin- and RSL3-induced ferroptosis.

### TetC Exerts Dose- and Time-Dependent Proliferation Inhibitory Effects on BC Cells

**FIGURE 2:**
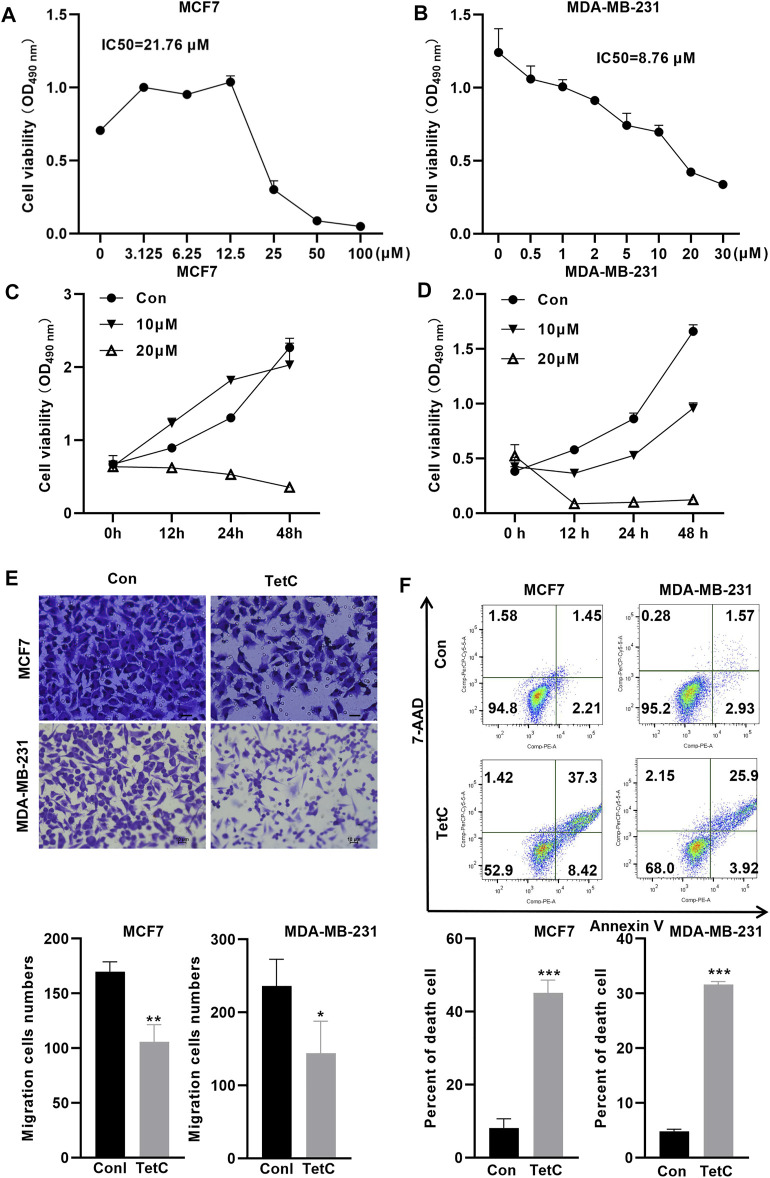
TetC exerts dose- and time-dependent proliferation inhibitory effects on BC cells. **(A,B)** IC50 of TetC in MCF7 and MDA-MB-231 cells was determined using MTT assay. **(C,D)** MCF7 and MDA-MB-231 cells were treated with 20 and 10 μM of TetC for 0, 12, 24, and 48 h. MTT assay showed that TetC exerted dose- and time-dependent proliferation inhibitory effects on MCF7 and MDA-MB-231 cells. **(E)** Transwell assay showed that TetC significantly suppressed BC cell migration. **(F)** Flow cytometry assay indicated that TetC induced MCF7 and MDA-MB-231 cell death (*n* = 3 independent repeats). ^*^ *p* < 0.05; ^**^ *p* < 0.01; ^***^ *p* < 0.001.

We further explored the toxic effect of TetC on BC cells using MTT assay. The IC50 values of TetC in MCF7 and MDA-MB-231 cells at 24 h were 21.76 and 8.76 μmol/l, respectively (Figures 2A,B). Then, we treated MCF7 and MDA-MB-231 BC cells with 20 and 10 μM TetC for 12, 24, and 48 h. MTT assay showed that TetC exerted dose- and time-dependent toxicity and proliferation inhibitory effects on MCF7 and MDA-MB-231 cells (Figures 2C,D). Meanwhile, transwell assay showed that 20 and 10 μM TetC significantly suppressed cell migration (Figure 2E). In addition, flow cytometry demonstrated that the number of dead cells was significantly upregulated in MCF7 and MDA-MB-231 cells (Figure 2F).

### TetC Drives Ferroptosis in BC Cells

**FIGURE 3:**
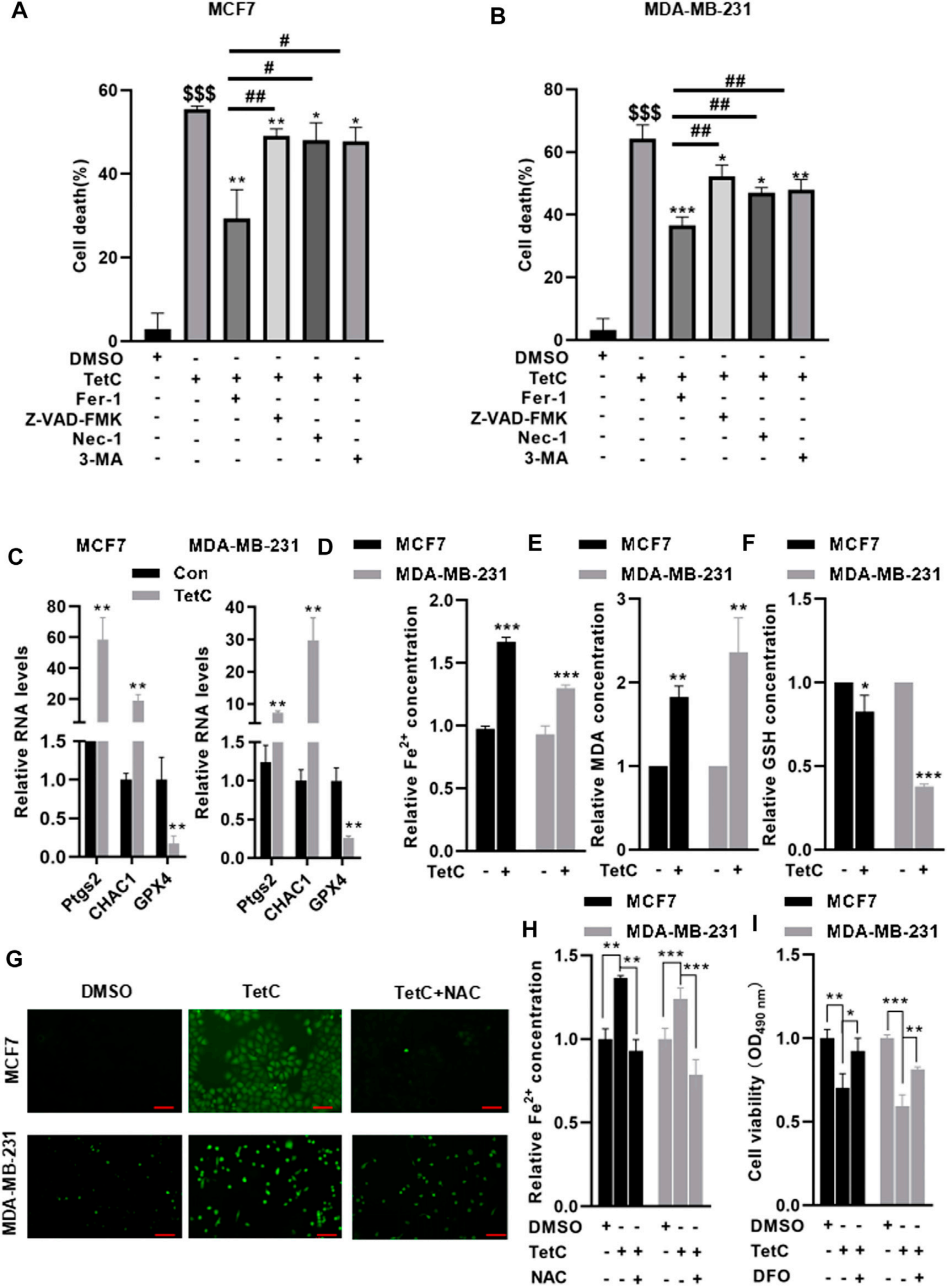
TetC drives ferroptosis in BC cells. MCF7 and MDA-MB-231 cells were preincubated with or without 1 μM Fer-1, 20 μM Z-VAD-FMK, 20 μM Nec-1, and 20 μM 3-MA for 1 h. Then, MCF7 and MDA-MB-231 cells were treated with 20 μM or 10 μM TetC for 24 h. MTT assay showed that compared with z-VAD-FMK, Nec-1, and 3-MA, Fer-1 demonstrated the potent cell death inhibitory effects in MCF7 **(A)** and MDA-MB-231 **(B)** cells. **(C)** RT-PCR analysis showed that TetC significantly upregulated the mRNA levels of Ptgs2 and Chac1 in BC cells but reduced GPX4 mRNA levels compared with those of control. TetC elevated the intracellular contents of Fe^2+^ **(D)** and MDA **(E)** but decreased the contents of GSH **(F)** in MCF7 and MDA-MB-231 cells. MCF7 and MDA-MB-231 cells were preincubated with 10 μM NAC or 10 μM DFO for 2h, followed by TetC treatement for another 24 h. DCFH-DA staining showed that TetC-induced accumulation of ROS could be largely attenuated by preincubation with NAC in both MCF7 and MDA-MB-231 cells; scale bar, 100 µm. **(G)** Upregulation of Fe^2+^ induced by TetC was also reversed by NAC preincubation in MCF7 and MDA-MB-231 cells **(H)**. **(I)** MTT assay showed that DFO significantly reversed TetC-induced cell death (*n* = 3 independent repeats). ^$$$^ *p* < 0.001 vs. DMSO; ^*^ *p* < 0.05; ^**^ *p* < 0.01; ^***^ *p* < 0.001 vs. TetC; ^#^ *p* < 0.05; ^##^ *p* < 0.01 vs. TetC + Fer-1.

Next, we explored which form of cell death could be driven by TetC in MCF7 and MDA-MB-231 cells. TetC was combined with various cell death inhibitors including Z-VAD-FMK (an apoptosis inhibitor), necrostatin-1 (Nec-1, a necroptosis inhibitor), 3-methyladenine (3-MA, an autophagy inhibitor), and Fer-1 (a ferroptosis inhibitor) in MCF7 and MDA-MB-231 cells (Figures 3A,B). Compared with TetC alone, preincubation with Z-VAD-FMK, Nec-1, and 3-MA decreased TetC-induced cell death with approximately 6.43%, 7.47%, and 7.78%, respectively, in MCF7 and MDA-MB-231 cells. In contrast, TetC-induced cell death could be attenuated by Fer-1 with about 26.15% in MCF7 and 16.75% in MDA-MB-231 cells. Strikingly, compared with Z-VAD-FMK, Nec-1, and 3-MA, Fer-1 demonstrated the most potent cell death inhibitory effects in MCF7 and MDA-MB-231 cells (Figures 3A,B). We then tested the effects of TetC on ferropotsis-related markers, including prostaglandin endoperoxide synthase 2 (Ptgs2), ChaC glutathione-specific gamma-glutamylcyclotransferase 1 (CHAC1), and GPX4. Compared with the control group, TetC significantly upregulated the mRNA levels of Ptgs2 and CHAC1 but reduced the GPX4 mRNA levels in BC cells (Figure 3C). Meanwhile, TetC elevated the intracellular contents of Fe^2+^ and MDA (Figures 3D,E) but decreased the levels of GSH in MCF7 and MDA-MB-231 cells (Figure 3F). DCFH-DA staining showed that TetC-induced accumulation of ROS could be largely attenuated by preincubation with N-acetyl-cysteine (NAC), an antioxidant, in both MCF7 and MDA-MB-231 cells (Figure 3G). Meanwhile, the upregulation of Fe^2+^ induced by TetC was also reversed by NAC preincubation in MCF7 and MDA-MB-231 cells (Figure 3H). Deferoxamine (DFO), an iron chelator, was added to emphasize the role of iron in TetC cytotoxicity. Our data showed that DFO significantly reversed TetC-induced cell death (Figure 3I). These findings indicated that both ROS and iron levels play a key role in TetC cytotoxicity for BC cells.

### TetC Enhances Erastin- and RSL3-Induced Ferroptosis in BC Cells

**FIGURE 4:**
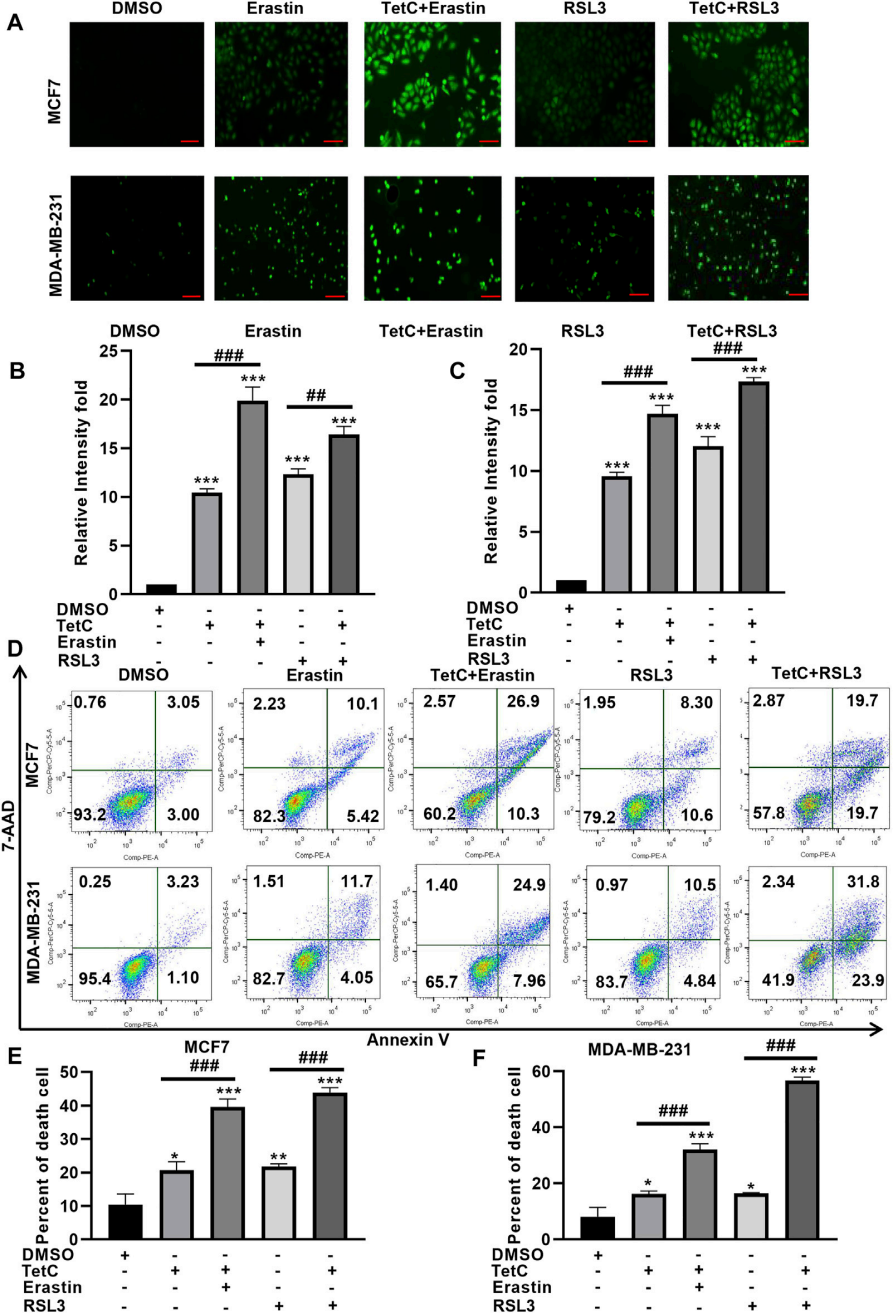
TetC enhances erastin- and RSL3-induced ferroptosis in BC cells. **(A–C)** DCFH-DA staining showed that a combination of TetC and erastin or RSL3 enhanced ROS generation in MCF7 and MDA-MB-231 cells, compared with those of erastin or RSL3 alone; scale bar, 100 µm. **(D–F)** Annexin V/7-AAD assay showed that erastin- and RSL3-induced ferroptotic damage could be further enhanced after combination with TetC in BC cells (*n* = 3 independent repeats). ^*^ *p* < 0.05; ^**^ *p* < 0.01; ^***^ *p* < 0.001 vs DMSO; ^##^ *p* < 0.01; ^###^ *p* < 0.001 vs erastin or RSL3 alone.

Then, we explored whether TetC could exert synergistic effects of the ferroptotic damage induced by RSL3 and erastin. DCFH-DA staining showed that the combination of TetC and erastin or RSL3 enhanced ROS generation in MCF7 and MDA-MB-231 cells, compared with erastin or RSL3 alone (Figures 4A–C). Meanwhile, Annexin V/7-AAD assay showed that erastin and RSL3 significantly elevated MCF7 and MDA-MB-231 cell death (Figures 4D–F), and such ferroptotic damage could be further enhanced after combination with TetC in BC cells (Figures 4D–F). These observations indicated that TetC synergizes with erastin and RSL3 to aggravate ferroptosis in BC cells.

### TetC Activates NCOA4-Mediated Ferritinophagy and Inhibits GPX4 in BC Cells

**FIGURE 5:**
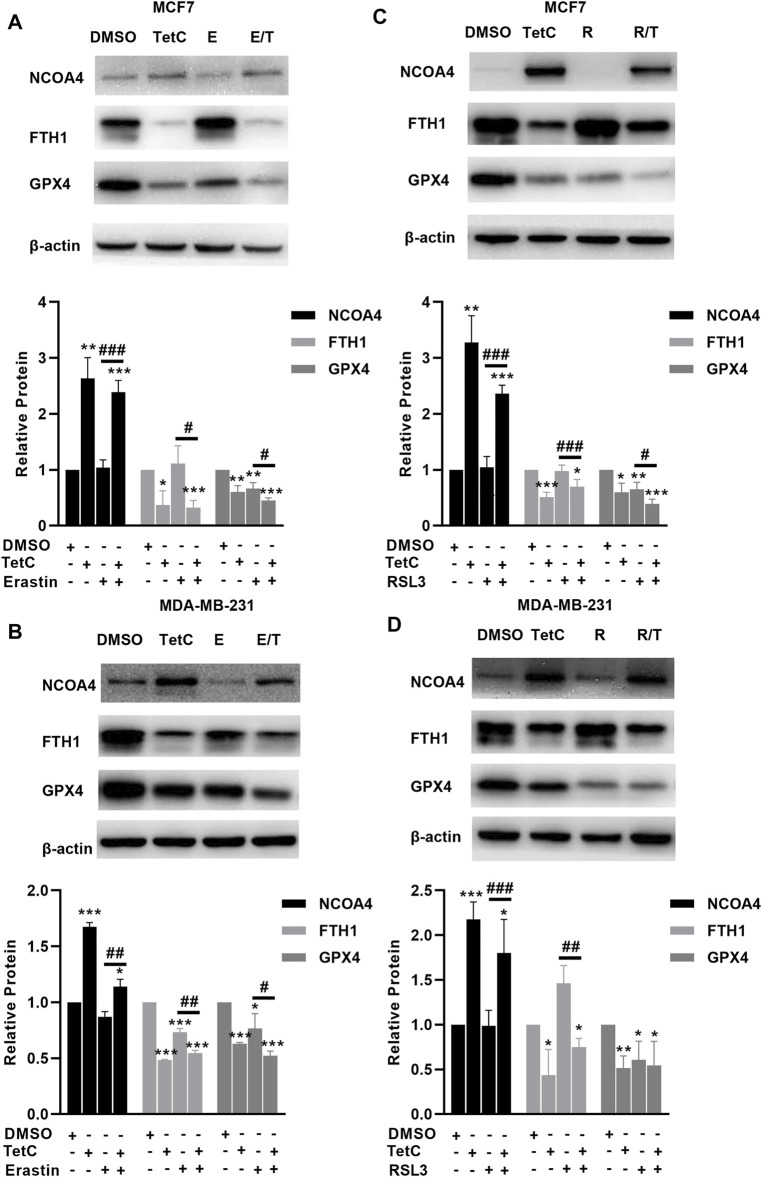
TetC activates NCOA4-mediated ferritinophagy and inhibits GPX4 in BC cells. Western blot assay was performed to analyze the effects of TetC on the genes related to ferroptosis compared with erastin **(A,B)** or RLS3 **(C,D)** alone in MCF7 and MDA-MB-231 cells (*n* = 3 independent repeats). *p < 0.05; **p < 0.01; ***p < 0.001 vs. DMSO; ^#^ *p* < 0.05; ^##^ *p* < 0.01 vs. erastin or RSL3 alone.

We then explored the effects of TetC on genes involved in the canonical and noncanonical pathways of ferropotosis. In MCF7 and MDA-MB-231 cells, TetC, erastin, or RSL3 alone significantly suppressed the expression of GPX4 (Figures 5A–D). Moreover, the combination of TetC and erastin or RSL3 further reduced the protein levels of GPX4 compared with erastin or RLS3 alone in MCF7 and MDA-MB-231 cells (Figures 5A–D). In comparison, erastin and RSL3 treatment did not change the expression of NCOA4 and FTH1 in MCF7 and MDA-MB-231 cells (Figures 5A–D). However, TetC was shown to elevate the expression of NCOA4 and reduce the expression of FTH1 in MCF7 and MDA-MB-231 cells (Figures 5A–D). These observations indicated that TetC-induced BC cell death was achieved *via* activating NCOA4-mediated ferritinophagy and inhibiting GPX4.

### FTH1 Degradation is Achieved by TetC-Induced Ferritophagy in BC Cells

**FIGURE 6:**
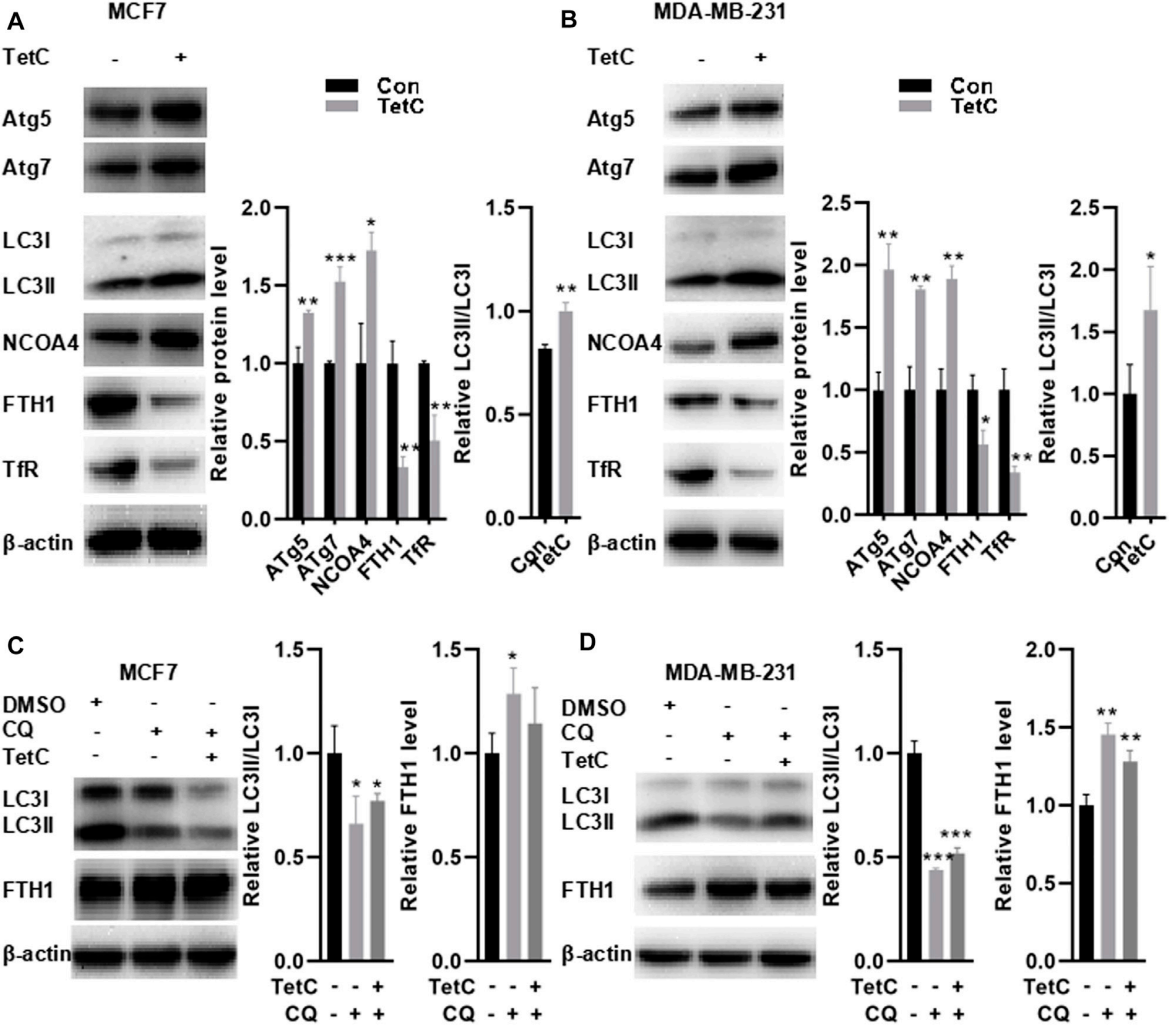
FTH-1 degradation is achieved by TetC-induced ferritophagy in BC cells. MCF7 and MDA-MB-231 cells were treated 20 and 10 μM TetC for 24 h in the presence or absence of 10 μM chloroquine (CQ). Western blot assay showed that TetC significantly elevated the expression of ATG5, ATG7, and NCOA4 but decreased FTH1 and TfR protein levels in MCF7 **(A)** and MDA-MB-231 cells **(B)**. TetC-induced degradation of FTH-1 expression was obviously rescued by blocking the lysosome function with CQ in MCF7 **(C)** and MDA-MB-231 **(D)** cells. ^*^ *p* < 0.05; ^**^ *p* < 0.01; ^***^ *p* < 0.001 vs DMSO.

Studies have indicated that autophagy triggers NCOA4-mediated ferritinophagy (Kaur and Debnath, 2015; Zhang et al., 2018; Santana-Codina et al., 2021). Depletion of autophagy-related (ATG) genes (e.g., ATG5 or ATG7) is reported to suppress ferritin degradation, decrease free iron levels, and thus suppress the activation of NCOA4 (Liu et al., 2020). Hence, we explored the markers of autophagosome, including ATG5, ATG7, and microtubule associated protein one light chain 3 (LC3), thereby evaluating alteration in NCOA4 activation. As shown in Figures 6A,B, TetC significantly elevated the expression of ATG5, ATG7, and NCOA4 but decreased FTH1 and TfR protein levels in MCF7 and MDA-MB-231 cells. Meanwhile, the ratio between LC3II and LC3I was also enhanced in MCF7 and MDA-MB-231 cells treated with TetC compared with those of controls, indicating that autophagy altered NCOA4 activation in BC cells. To further evaluate whether TetC-induced reduction of FTH1 was achieved *via* ferritophagy, we blocked the lysosome function with 10 μM chloroquine (CQ). Our data showed that CQ inhibited autophagy as evidenced *via* decreased LC3II/LC3I ratio in MCF7 and MDA-MB-231 cells (Figures 6C,D). More importantly, TetC-induced degradation of FTH1 expression was obviously rescued by blocking the lysosome function with CQ in MCF7 and MDA-MB-231 cells (Figures 6C,D). The aforementioned results indicated that FTH1 degradation was achieved by TetC-induced ferritophagy in breast cancer cells.

### Fer-1 Alleviates TetC-Induced Ferroptosis in BC Cells

**FIGURE 7:**
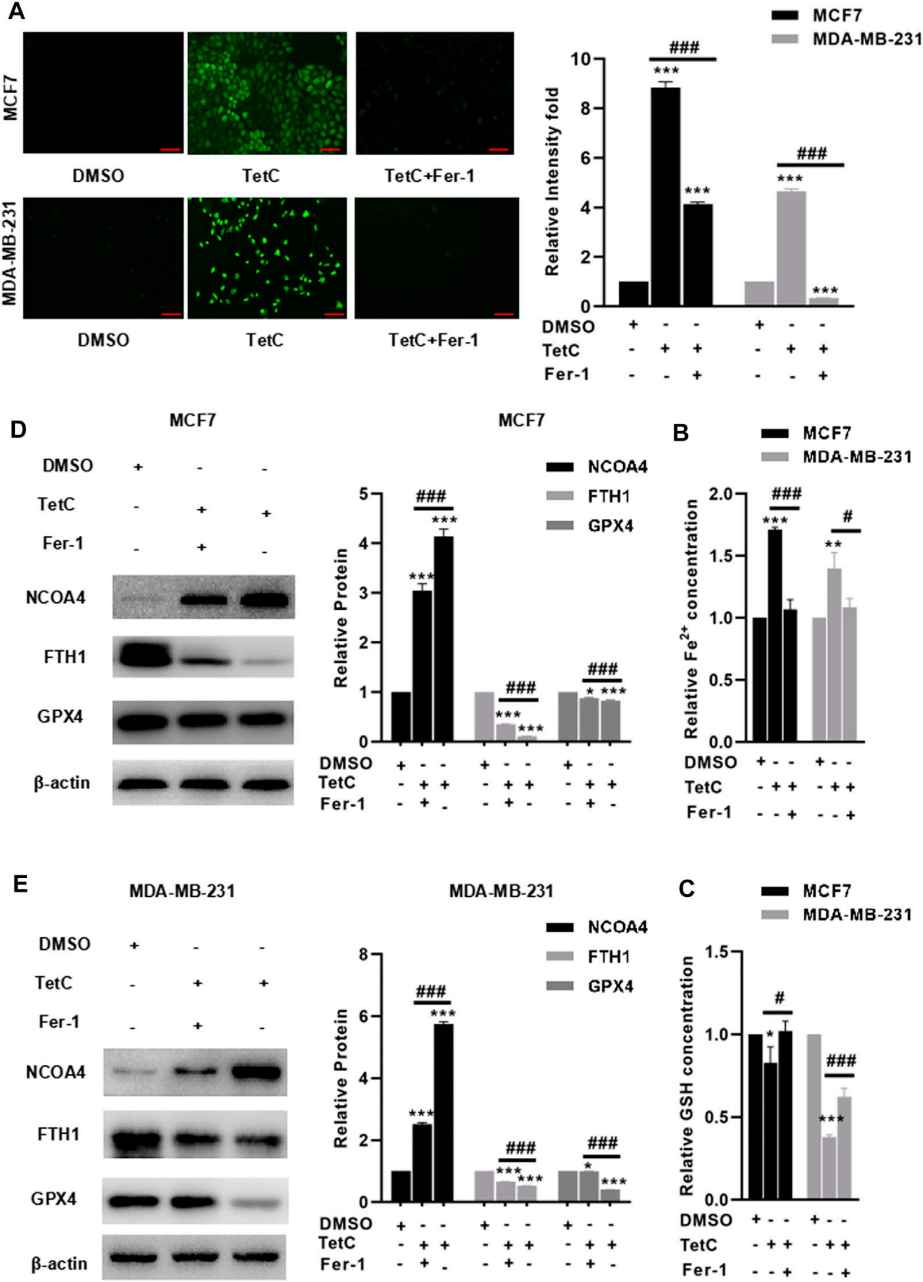
Fer-1 alleviates TetC-induced ferroptosis in BC cells. Preincubation with Fer-1 significantly reduced the intracellular ROS **(A)** and Fe^2+^ **(B)** in both MCF7 and MDA-MB-231 cells treated with TetC; scale bar, 100 µm. **(C)** There was an increase in GSH in the TetC-treated group, but addition of Fer-1 obviously reversed such an effect. Fer-1 treatment significantly antagonized TetC-induced elevation of NCOA4 and downregulation of GPX4 and FTH1 in MCF7 **(D)** and MDA-MB-231 **(E)** cells. *p < 0.05; **p < 0.01; ***p < 0.001 vs DMSO; ^#^ *p* < 0.05; ^###^ *p* < 0.001 vs TetC alone.

Finally, we explored the protective effects and the underlying mechanism of Fer-1 against TetC-induced ferroptosis in BC cells. As shown in Figures 7A,B, preincubation with Fer-1 significantly reduced the intracellular ROS and Fe^2+^ in both MCF7 and MDA-MB-231 cells treated with TetC. Meanwhile, there was a decrease in GSH in the TetC-treated group, but addition of Fer-1 obviously reversed such effects (Figure 7C). To elucidate the mechanism of the cytoprotective effect of Fer-1 in TetC-treated cells, the expression of GPX4, FTH1, and NCOA4 was investigated. Our data showed that the expression of NCOA4 increased, but the expression of GPX4 and FTH1 reduced after TetC treatment in MCF7 and MDA-MB-231 cells (Figures 7D,E). In contrast, Fer-1 treatment significantly antagonized such effects (Figures 7D,E).

## Discussion

Increasing evidence suggests that ferroptotic cell death plays a key role in BC tumor growth inhibition (Ma et al., 2016; Li Z et al., 2020). To develop therapeutic methods for targeting ferroptosis, increasing efforts have been made in identifying ferroptosis-inducing agents in cancer therapy (Ma et al., 2016; Li Z et al., 2020).

Tet is characterized by anti-allergenic and anti-inflammatory properties (Ding et al., 2021b). The anticancer effects of TetC have been reported in previous studies (Yu et al., 2019; Wang et al., 2020). Guo and Pei (2019) report that Tet significantly suppresses the tumor growth and induced autophagy in BC cells *via* PI3K/AKT/mTOR pathway. Wang et al. (2020) show that Tet exerts its anticancer activity in MDA-MB-231 cells *via* increasing apoptosis. Consistent with these findings, we also identified growth inhibitory effects of TetC in BC cells. Obviously, the two breast cancer cell lines used were sensitive to TetC treatment. However, whether TetC can also affect normal proliferating cells is a question worthy of consideration. In clinics, Tet is widely applied for the therapy of lung fibrosis, rheumatoid arthritis, silicosis, and cardiovascular disease in the Chinese population (Cheng et al., 2021). A previous study has also indicated that 10 μM Tet does not affect the viability of MPC5 podocytes (Ding et al., 2021b). In addition, preincubation with 20 μM Tet slightly reduced the viability of HaCaT human keratinocyte cells (Lin et al., 2019). In the present study, we demonstrated that the IC50 of TetC for MCF7 and MDA-MB-231 cells were 21.76 and 8.76 μmol/l, respectively, indicating that low dose of TetC significantly led to breast cancer cell death. Considering its extensive use in clinics, we propose that low dose of TetC exerts minimal toxicity in normal human cells and it may be specific for tumor cells in breast cancer patients, Herein, we combined TetC with different inhibitors, including Z-VAD-FMK, Nec-1, and 3-MA. Consistent with previous findings (Guo and Pei, 2019) (Wang et al., 2020), our data indicated that TetC could induce apoptosis and autophagy in BC cells. We then pre-incubated MCF7 and MDA-MB-231 cells with Fer-1, a potent inhibitor of ferroptosis. MTT assay showed that Fer-1 significantly reversed TetC-induced BC cell death. More importantly, compared with other inhibitors, Fer-1 demonstrated the most potent effects on improving TetC-induced BC cell death. Ptgs2 is a key enzyme involved in the synthesis of prostaglandins, which enhances the activity of peroxidase and ROS production (Li N et al., 2020). CHAC1 acts as a useful pharmacodynamic marker that inhibits system x_c−_ and triggers ferroptosis (Dixon et al., 2014). RT-PCR analysis demonstrated that TetC enhanced the mRNA level of Ptgs2 and CHAC1 in BC cells, indicating that ferroptosis plays a key role in TetC-induced cell death in BC cells.

We then examined the combined effects of TetC and erastin/RSL3 on MCF7 and MDA-MB-231 cells, respectively. Our data indicated that TetC and erastin/RSL3 synergistically elevated the ROS production and induced BC cell death. GPX4 is reported to suppress ferroptosis by inhibiting phospholipid peroxidation in erastin and RSL3-induced ferroptosis (Chu et al., 2020; Lee et al., 2021). A recent study indicates that GPX4 is increased in BC cancer tissues than in the normal control (Ding et al., 2021a). In addition, elevated expression of GPX4 is shown to be a novel prognostic biomarker for patients with BC receiving neoadjuvant chemotherapy (Sha et al., 2021). Blockage of GPX4 may be a novel strategy to trigger ferroptosis and improve drug resistance in BC cells (Sha et al., 2021). In the present study, we showed novel data that TetC reduced the expression of GPX4 in MCF7 and MDA-MB-231 cells. Importantly, TetC in combination with RSL3 or erastin further reduced the expression of GPX4 in BC cells compared with RSL3 or erastin alone. Hence, we proposed that ferroptosis induced by TetC is partially achieved *via* suppression of GPX4 in BC cells.

NCOA4 is a major regulator of ferritin levels and is hence involved in the process of ferritinophagy (Mancias et al., 2014; Santana-Codina and Mancias, 2018). Generally, NCOA4 interacts with the surface arginine of FTH1, which is then fused with the autophagy machinery *via* the nascent autophagosomes, thereby inducing ferritinophagy-dependent cell death (Gryzik et al., 2017). Emerging evidence has suggested that NCOA4 is a positive regulator in inducing ferroptosis *via* clearing the generation of intracellular free iron, GSH, and ROS (Bellelli et al., 2016). Here, we showed novel data that TetC elevated the expression of autophagosome markers, including ATG5, ATG7, and LC3II. Meanwhile, the expression of NCOA4 was also increased by TetC but reduced the expression of FTH1 and TfR in MCF7 and MDA-MB-231 cells. Moreover, blocking the lysosome function with CQ would rescue TetC-induced reduction of FTH1 in BC cells, indicating that FTH1 degradation is achieved through ferritophagy. Interestingly, treatment with erastin or RSL3 alone had little effect on NCOA4 and FTH1 levels in BC cells. This observation was consistent with a previous study that RSL3-induced cell death is independent of NCOA4-mediated ferritin degradation (Gryzik et al., 2021). Erastin is indicated to enhance ferritinophagy in HeLa cells overexpressing NCOA4 (Gryzik et al., 2021). In contrast, we did not find that erastin triggered ferritinophagy in MCF7 and MDA-MB-231 cells. This may be due to the fact that NCOA4-mediated ferritinophagy enhances erastin-induced ferroptosis *via* elevating intracellular iron content and ROS production (Gao et al., 2016; Hou et al., 2016). Thus, TetC also enhanced erastin- or RSL3-induced ferroptosis in BC cells *via* activation of the NCOA4-mediated ferritinophagy pathway.

However, there are limitations to the present study. First, since FTH1 degradation is an important mechanism in TetC-induced breast cancer cell death, whether FTH1 overexpression alters TetC cytotoxity in breast cancer cells deserves further study. Second, the cell lines need to be used in mice to determine whether TetC can inhibit tumor growth through ferroptosis. We will perform the *in vivo* assay in the following study. Third, SynergyFinder is a web application for analyzing drug dose–response matrix data. It is helpful to systematically determine the preclinical significance of pairwise drug combinations. In the following study, we will dedicatedly use SynergyFinder to distinguish if the combination of TetC with either RSL3 or erastin has a synergistic effect or just an additive effect.

**FIGURE 8:**
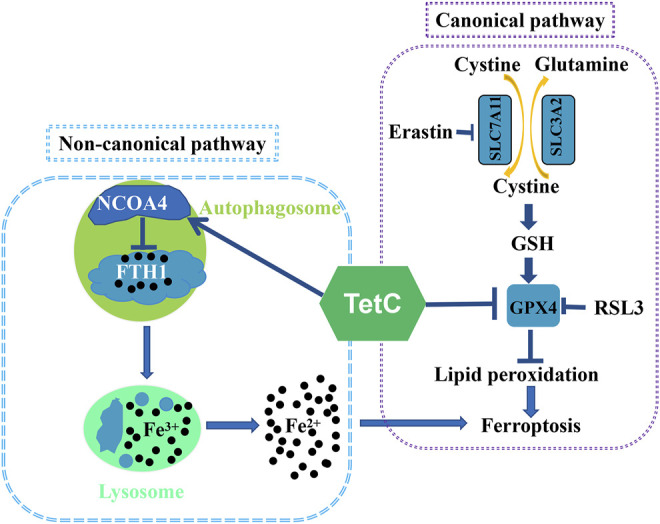
Mechanism by which TetC suppresses ferroptosis in BC cells.

In summary, we showed novel data that ferroptosis was a major contributor to TetC-induced cell death in BC cells. Moreover, TetC-induced ferroptotic cell death was achieved *via* suppressing GPX4 expression and activating NCOA4-mediated ferritinophagy (Figure 8). As both pathways are key regulators in ferroptosis, TetC may be of great potential for breast cancer therapy.

## Data Availability

The original contributions presented in the study are included in the article/Supplementary Material, further inquiries can be directed to the corresponding authors.
