# Supplementary material for: Tetrandrine Citrate Suppresses Breast Cancer via Depletion of Glutathione Peroxidase 4 and Activation of Nuclear Receptor Coactivator 4-Mediated Ferritinophagy
Source: Front Pharmacol. 2022 May 9;13:820593. doi: 10.3389/fphar.2022.820593 (PMC9124810; doi:10.3389/fphar.2022.820593)

3G-MCF7-DMSO

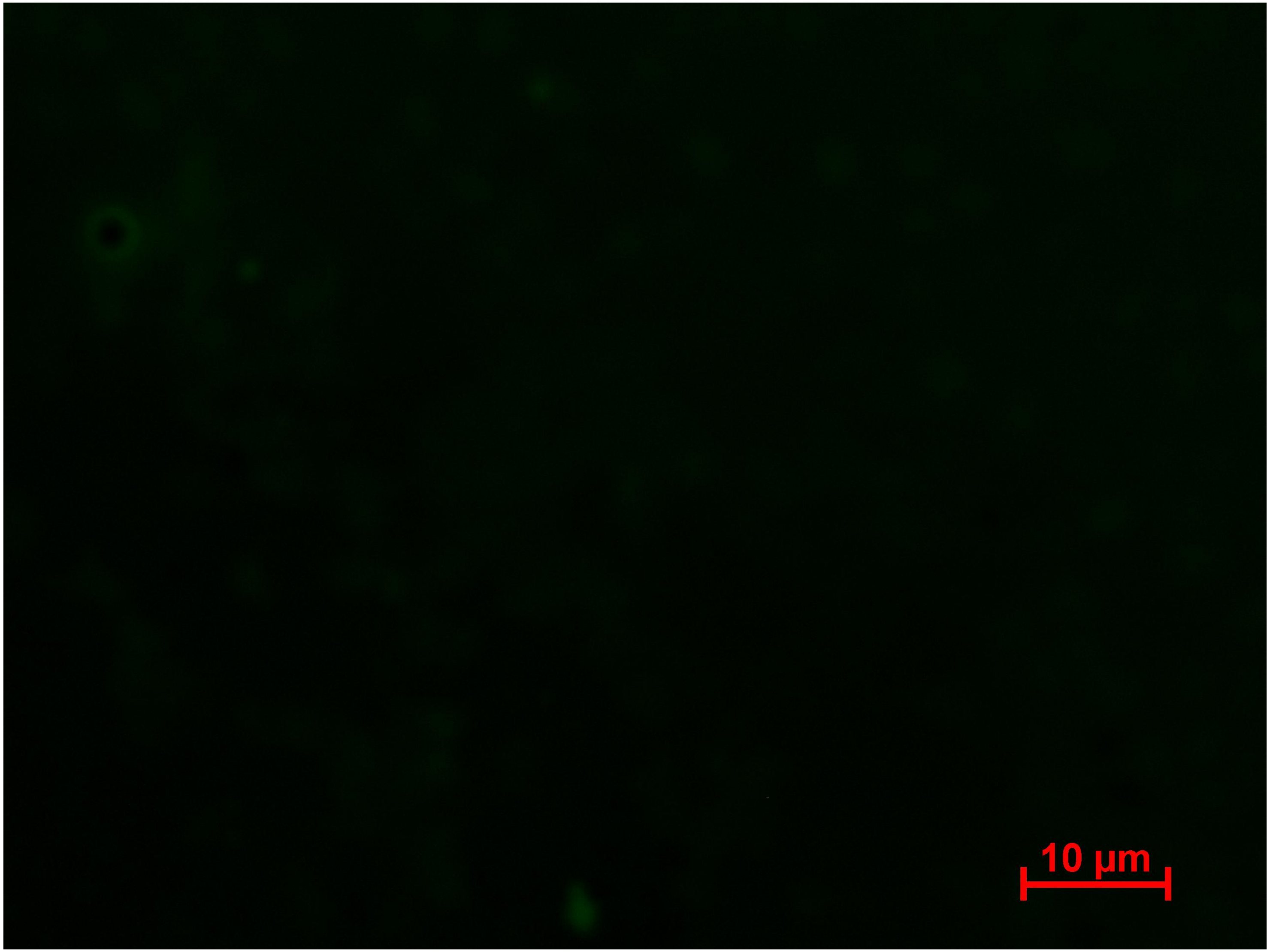

3G-MCF7-TetC

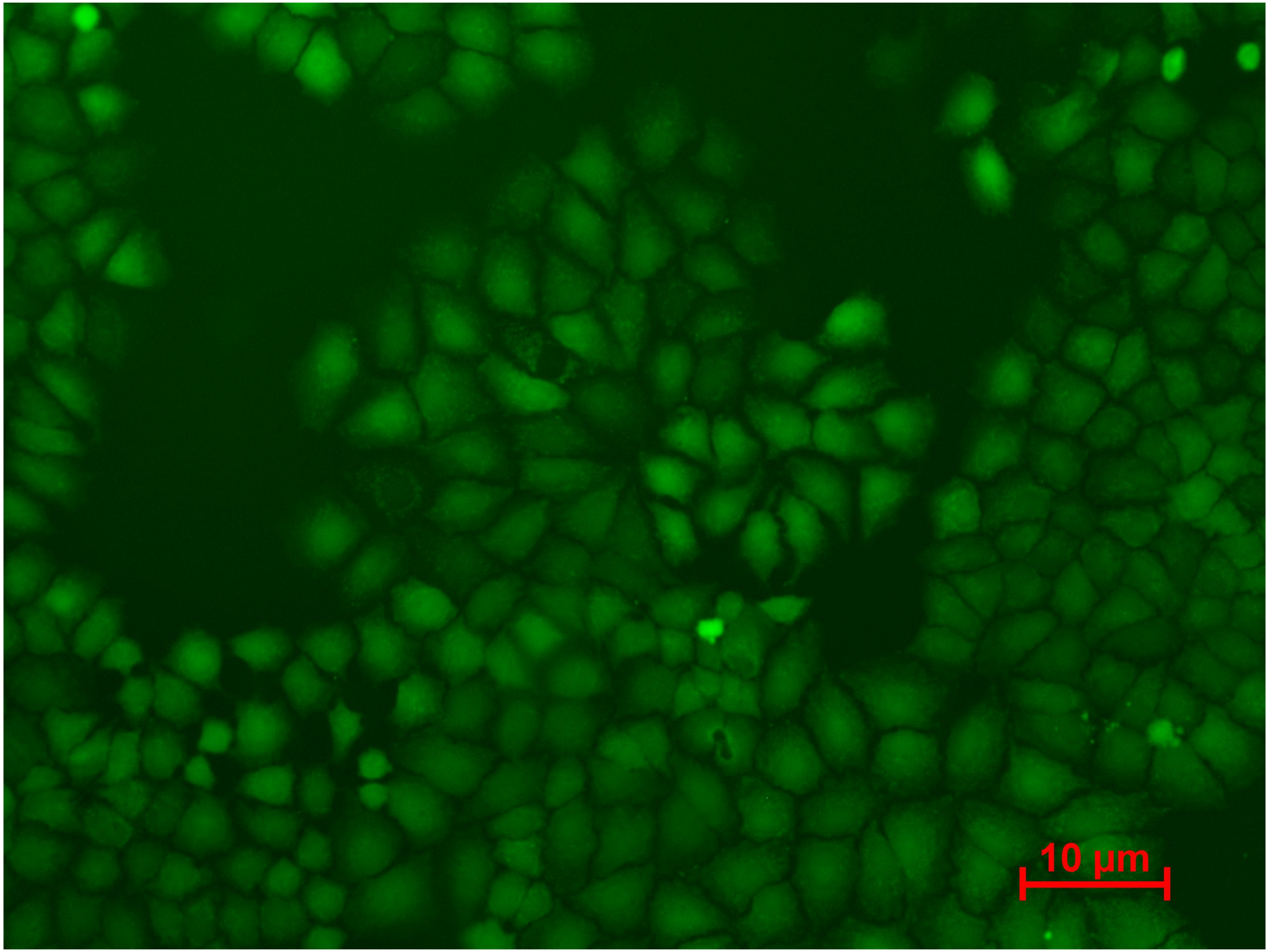

3G-MCF7-TetC+NAC

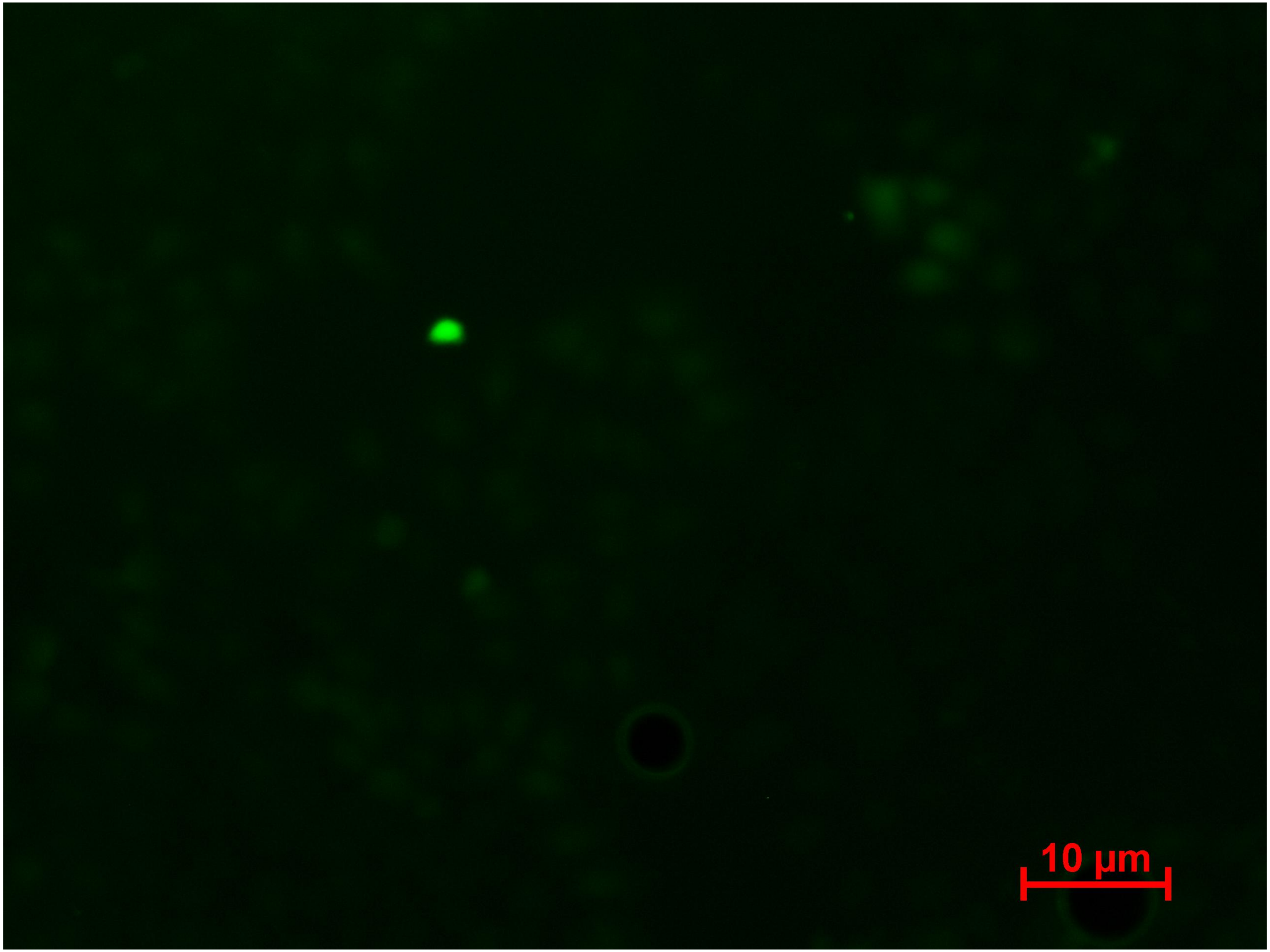

3G-MDA-MB-231-DMSO

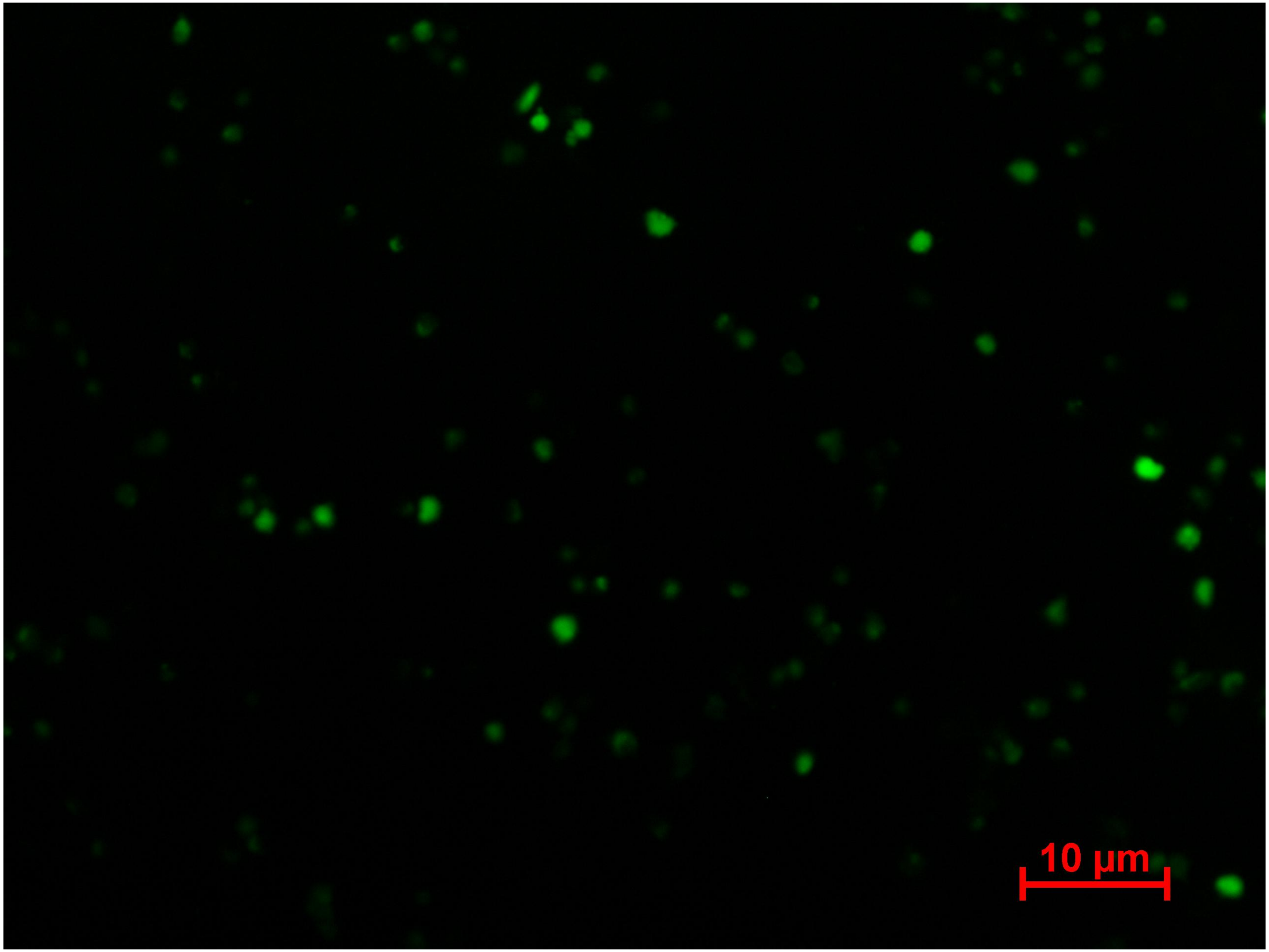

3G-MDA-MB-231-TetC

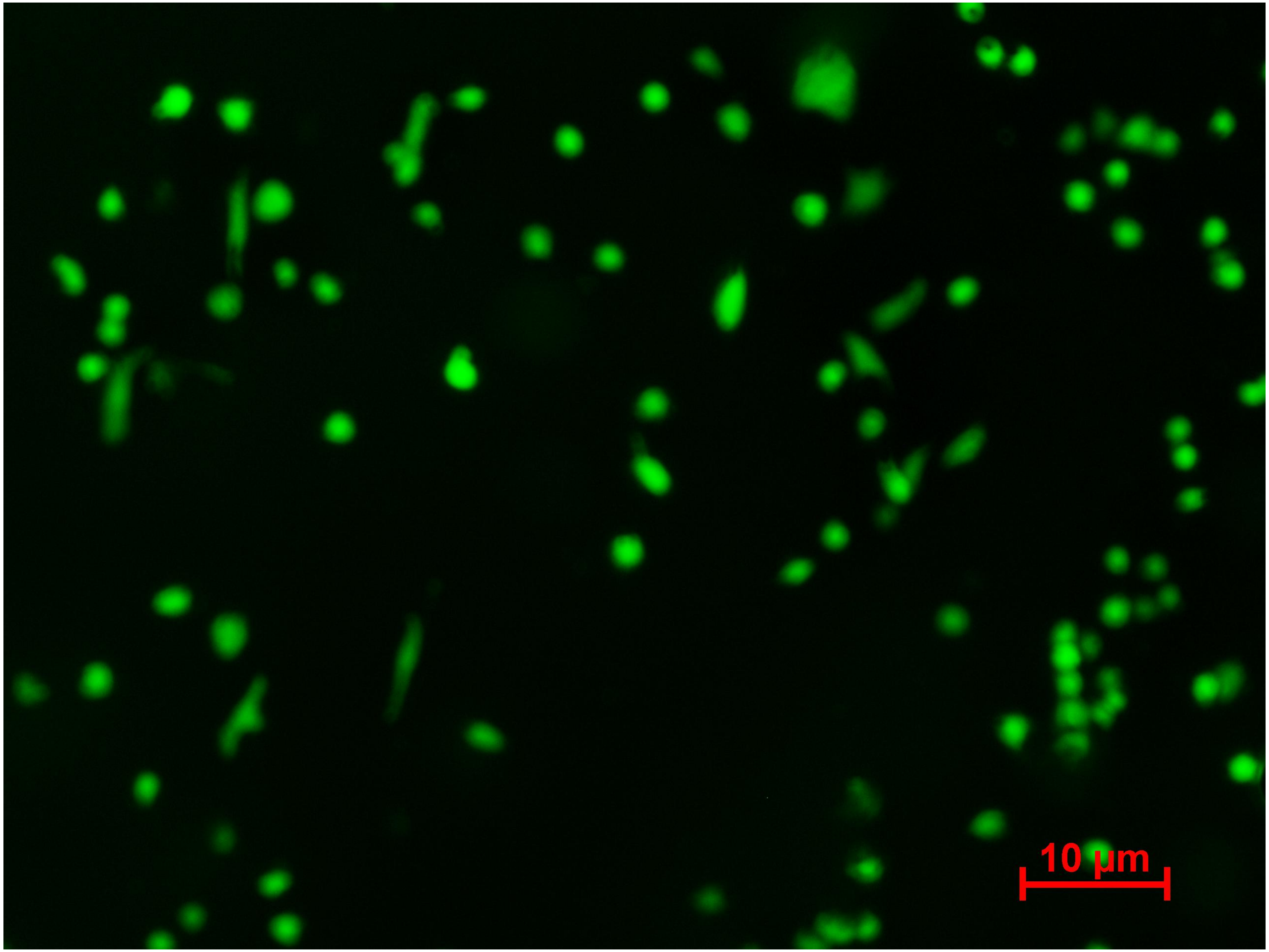

3G-MDA-MB-231-TetC+NAC

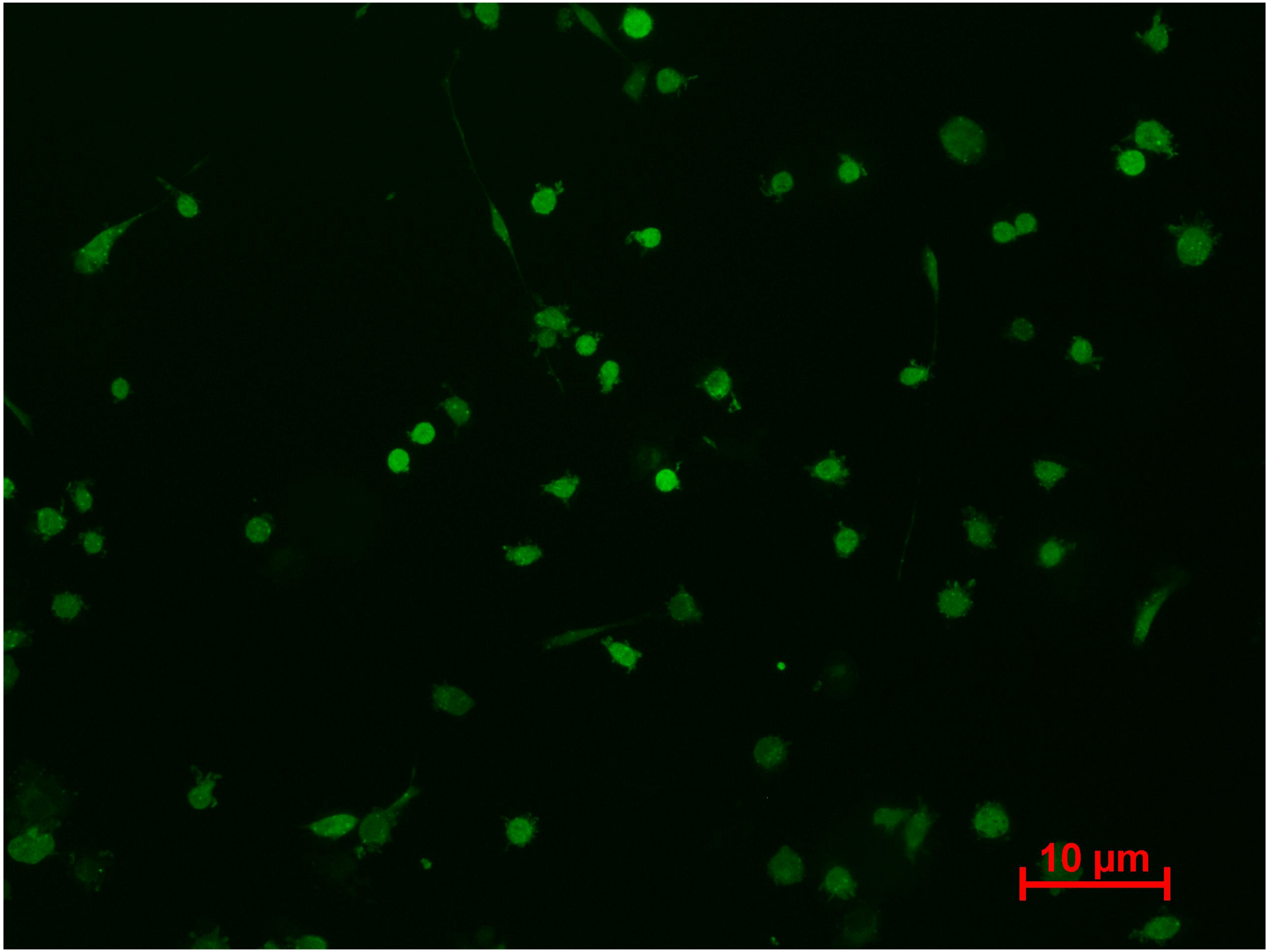

6A-MCF7-β-actin

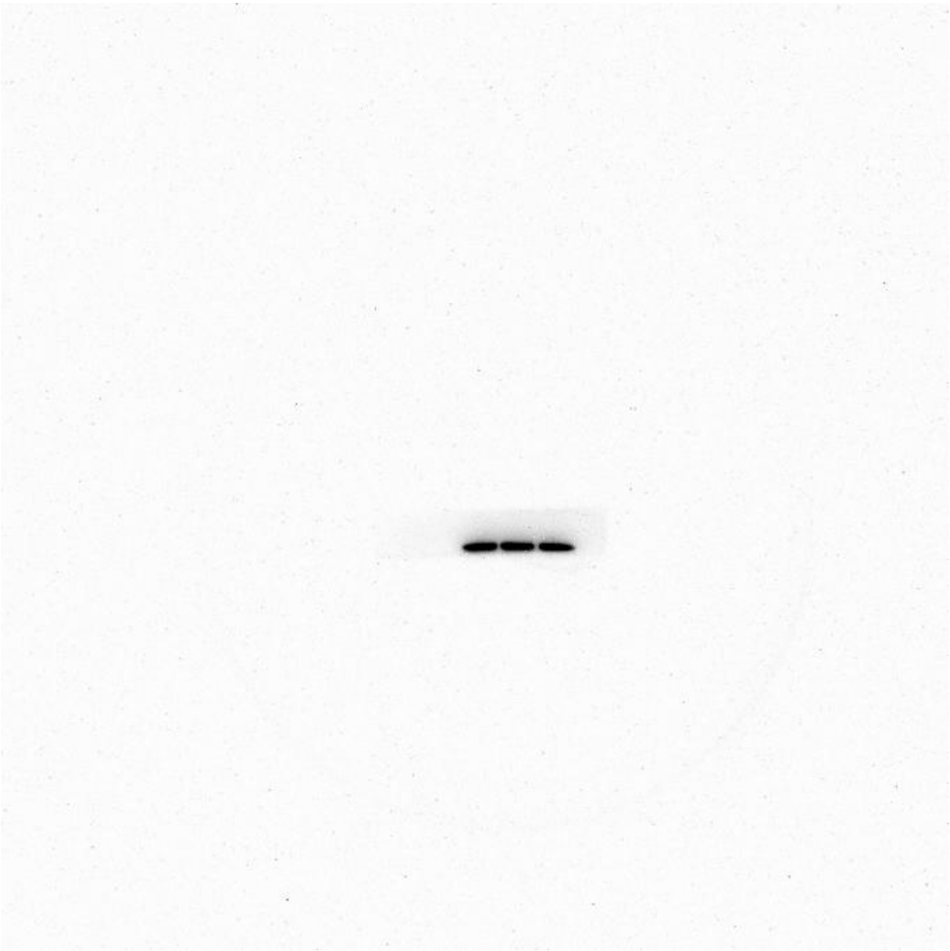

6A-MCF7-Atg5

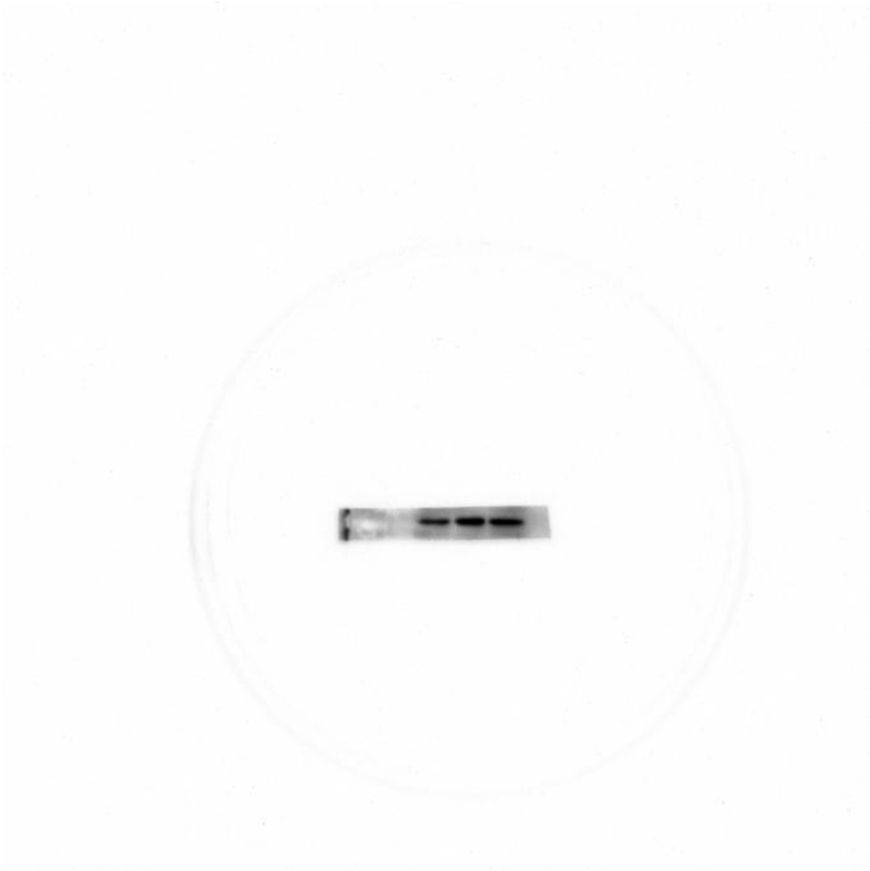

6A-MCF7-Atg7

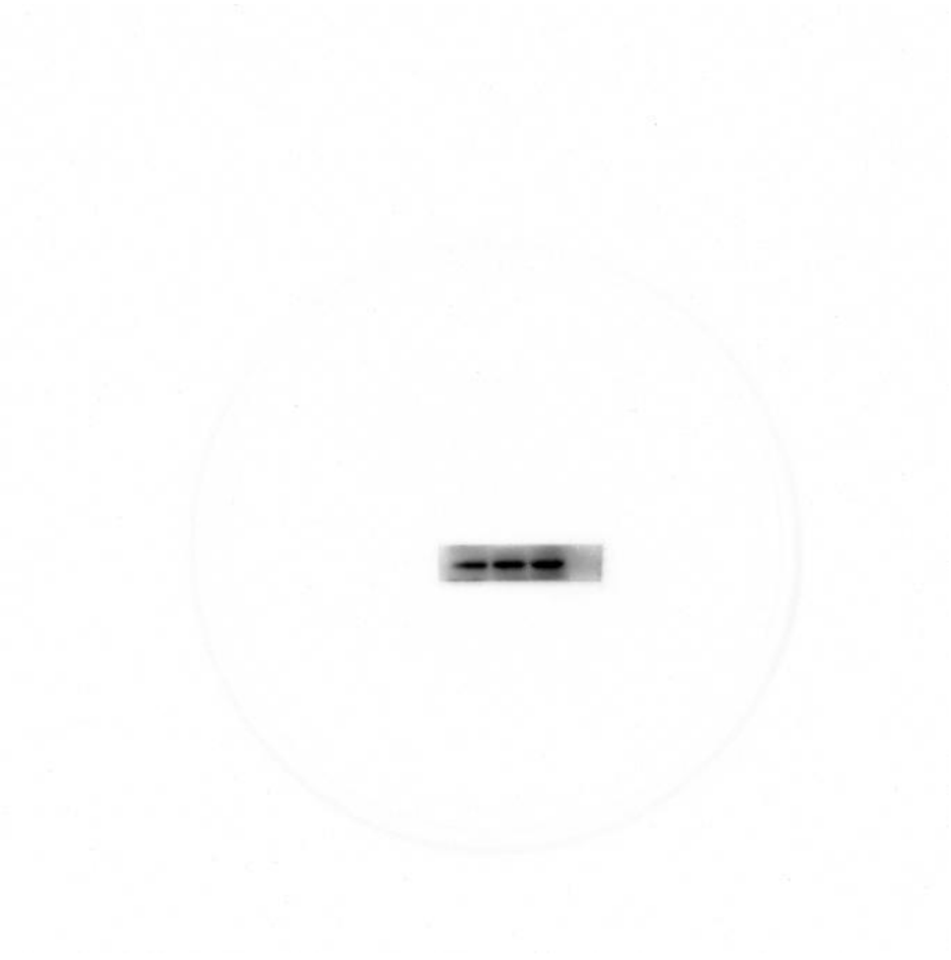

6A-MCF7-FTH1

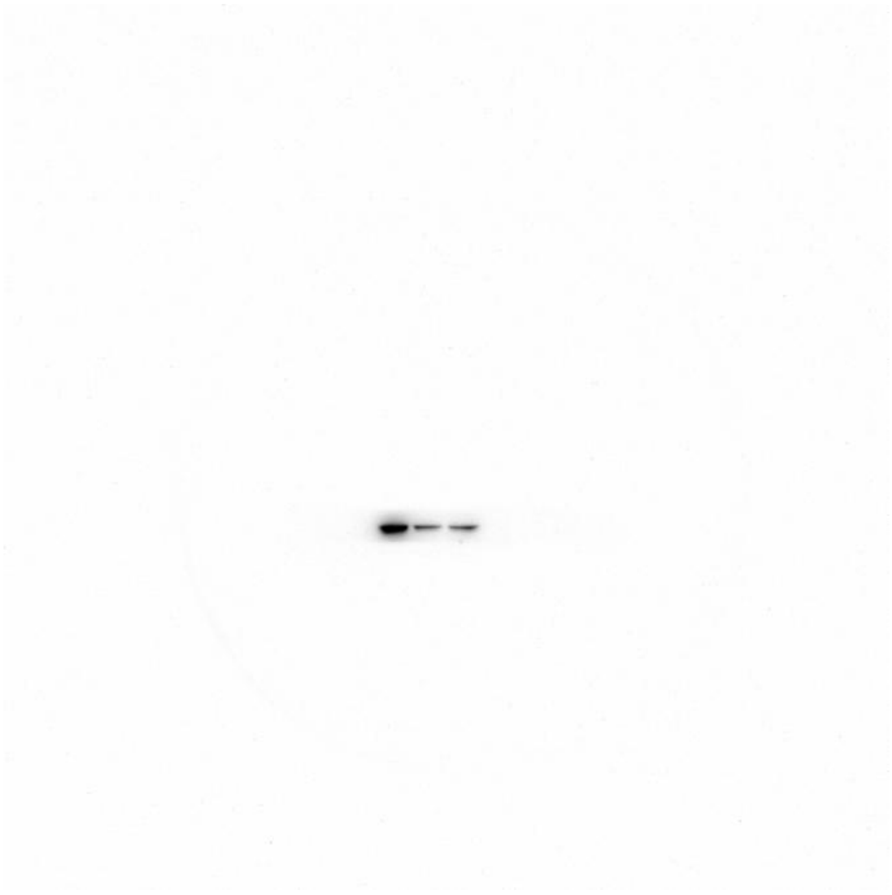

6A-MCF7-LC3

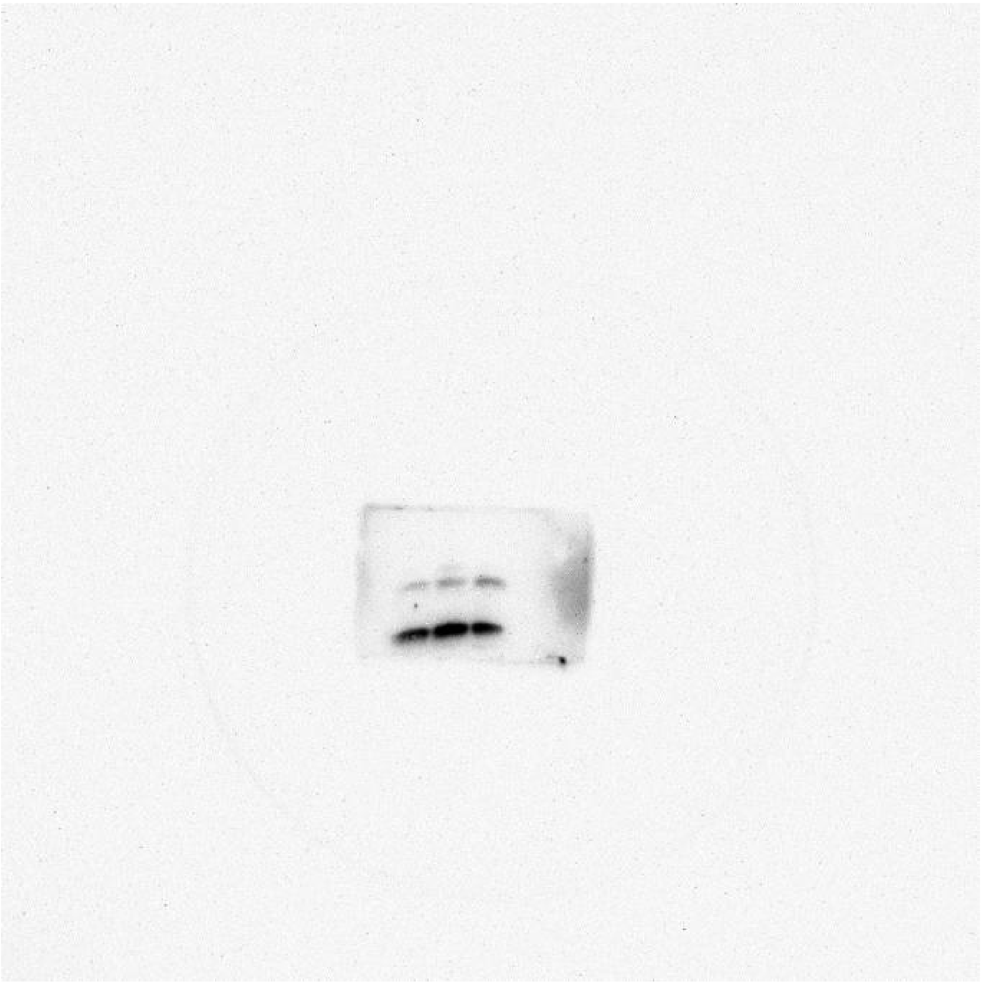

6A-MCF7-NCOA4

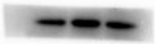

6A-MCF7-TfR

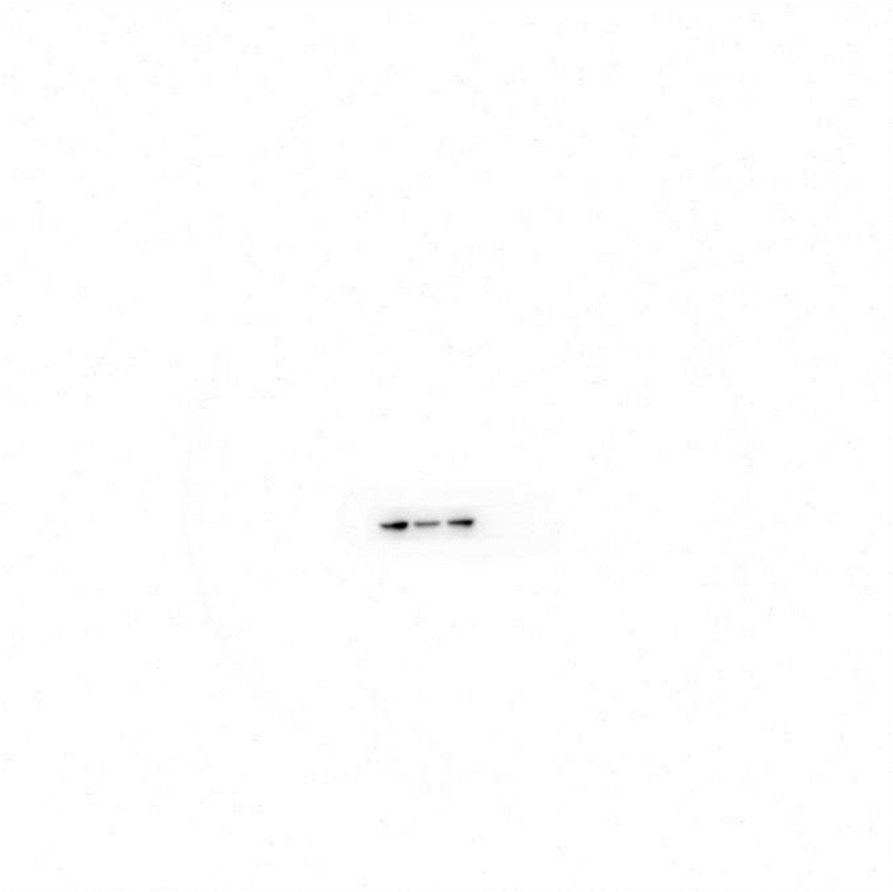

6B-MDA-MB-231-β-actin

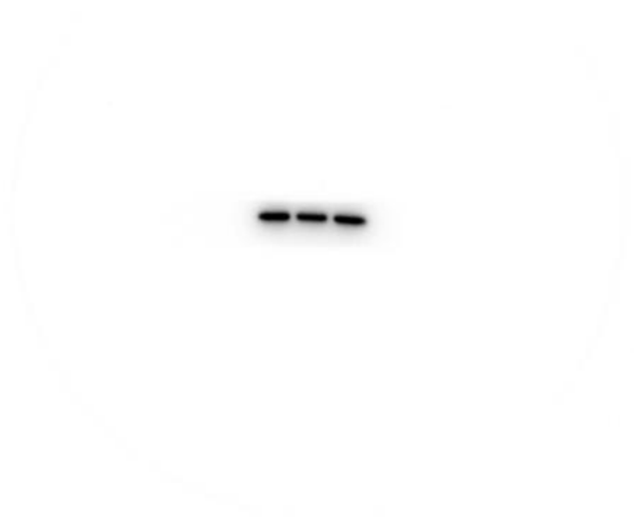

6B-MDA-MB-231-Atg5

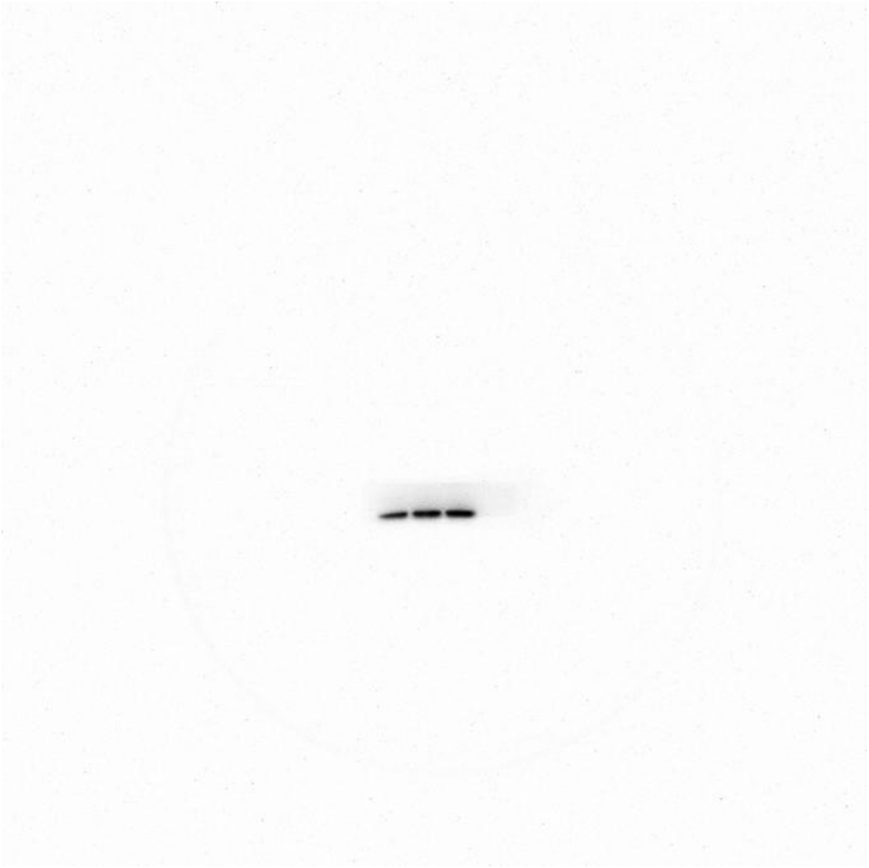

6B-MDA-MB-231-Atg7

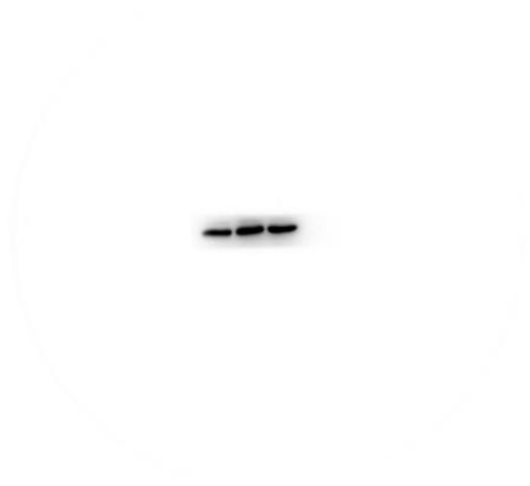

6B-MDA-MB-231-FTH1

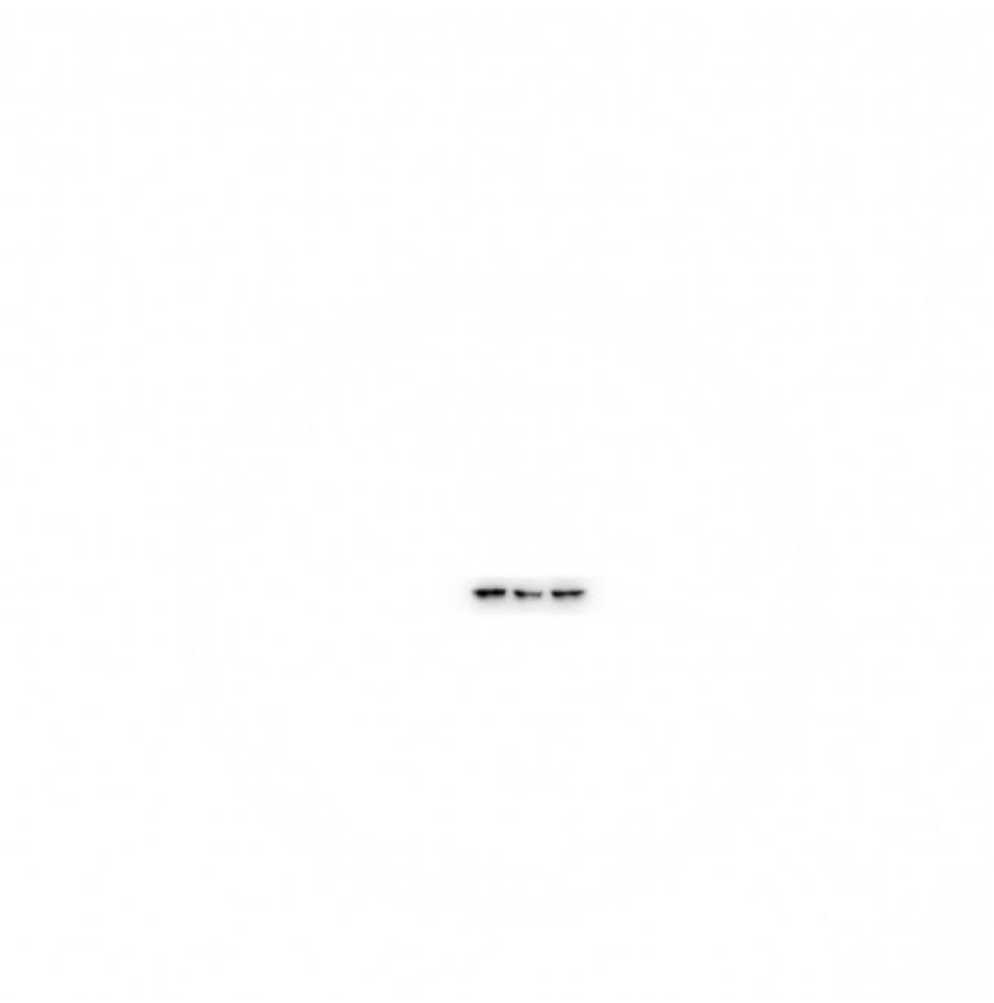

6B-MDA-MB-231-LC3

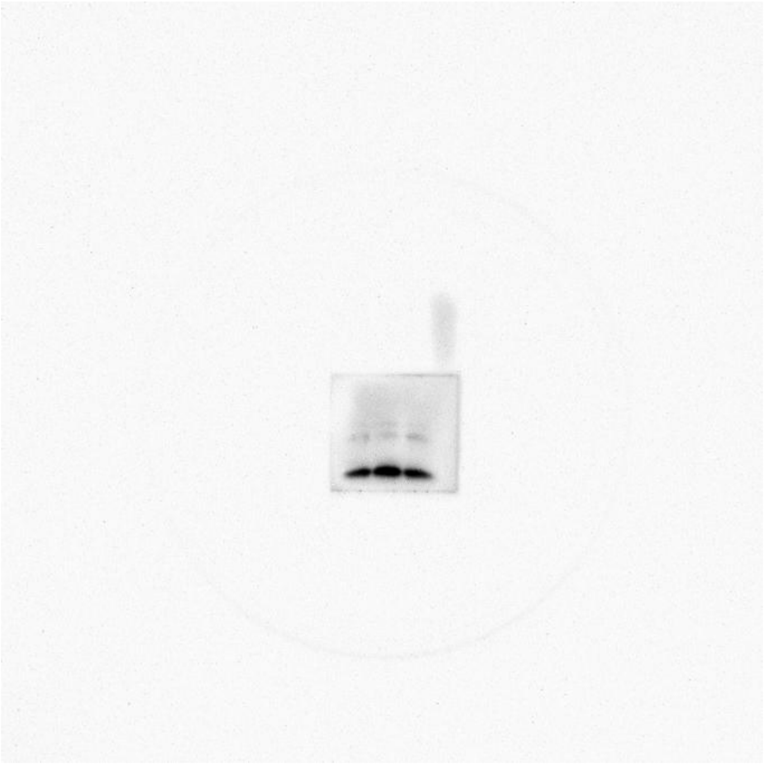

6B-MDA-MB-231-NCOA4

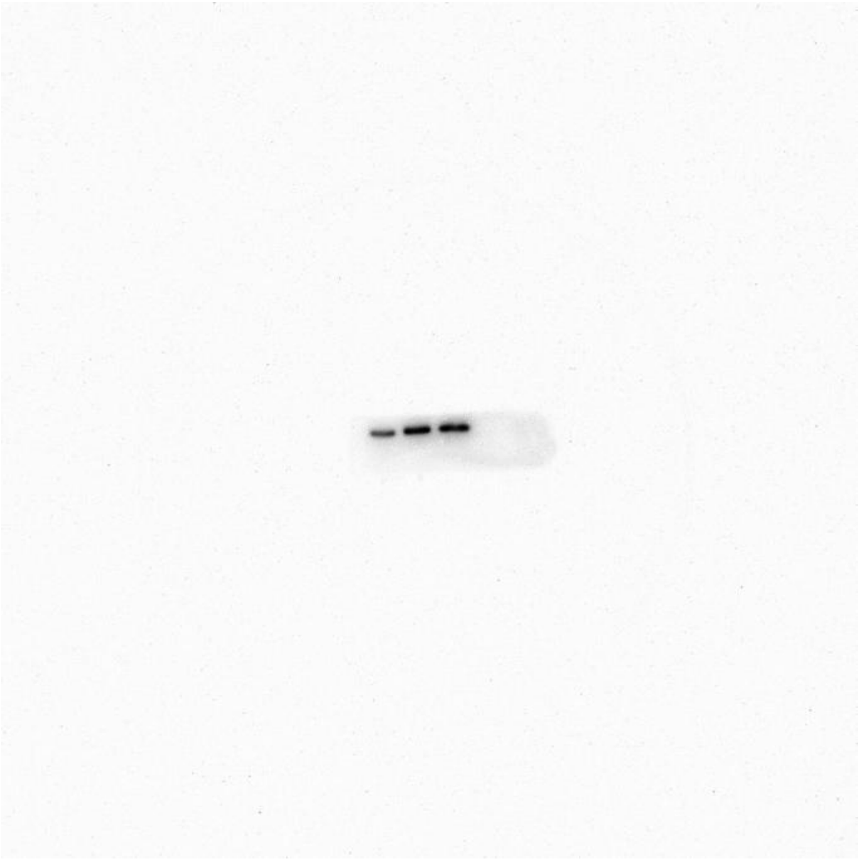

6B-MDA-MB-231-TfR

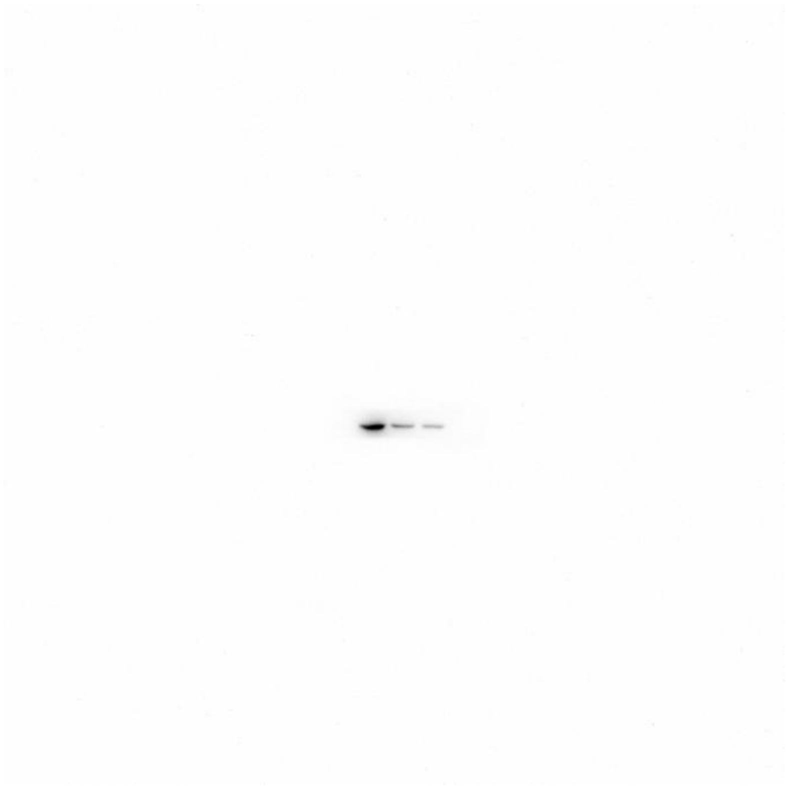

6C-MCF7-β-actin

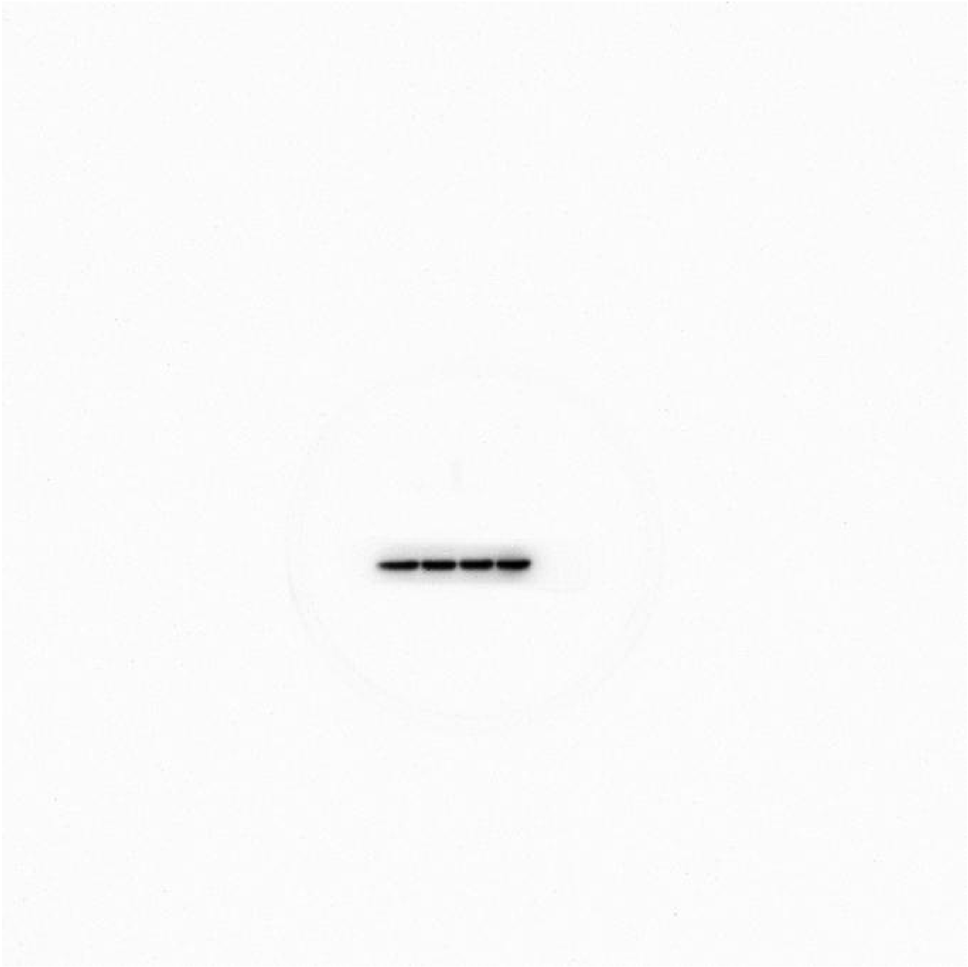

6C-MCF7-LC3

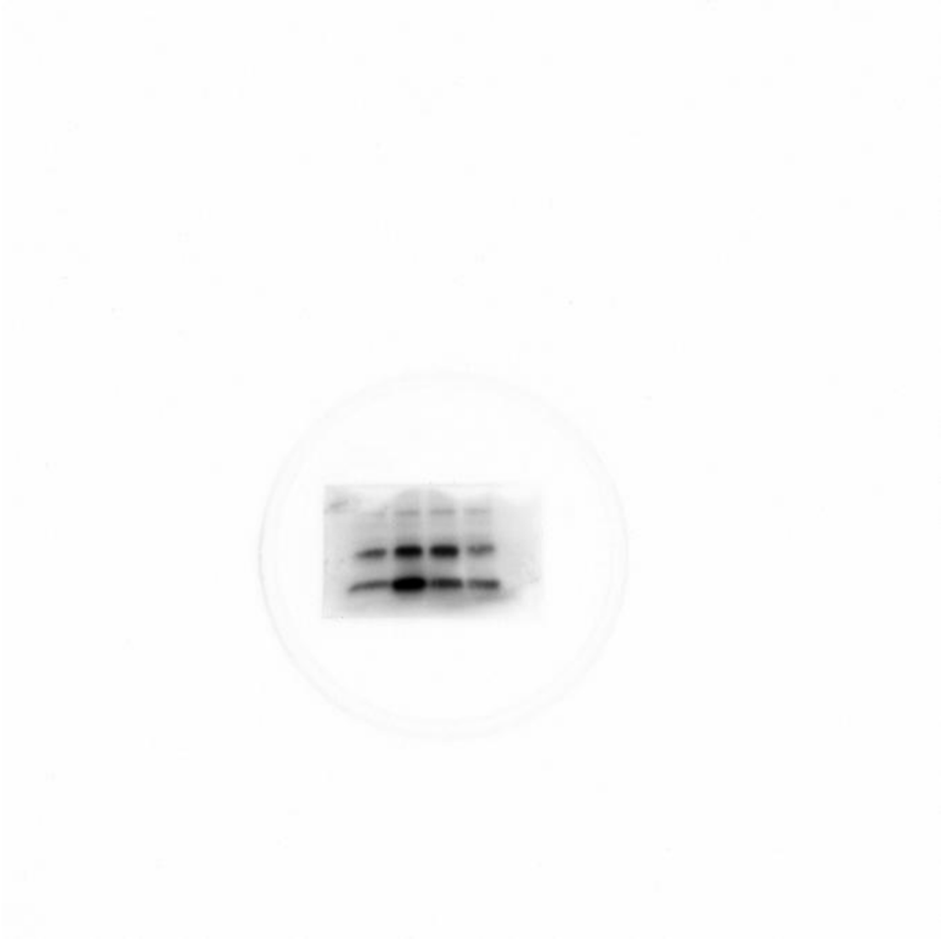

6C-MCF7-FTH1

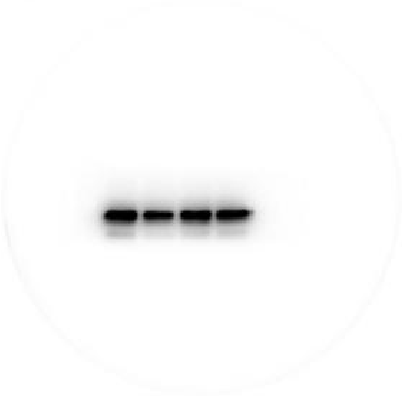

6D-MDA-MB-231-β-actin

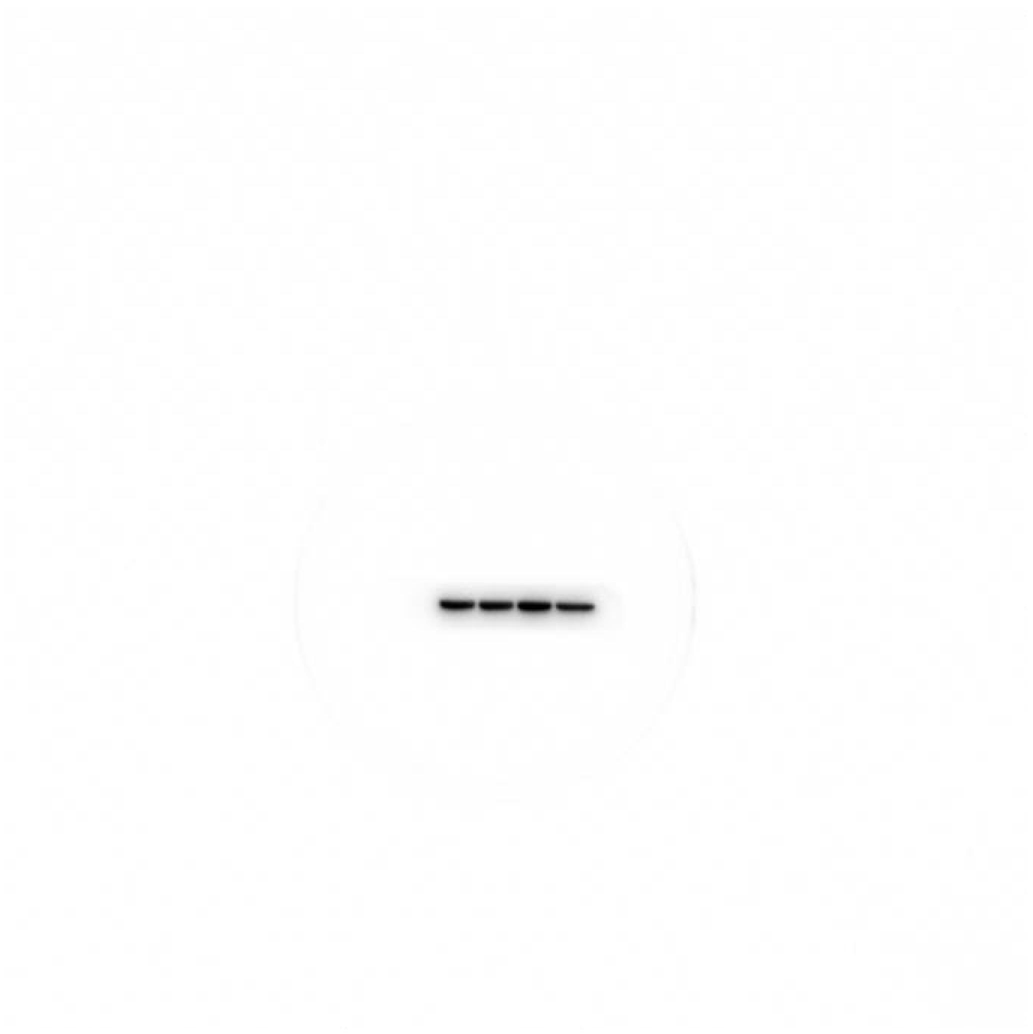

6D-MDA-MB-231-LC3

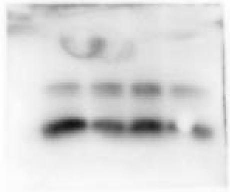

6D-MDA-MB-231-FTH1

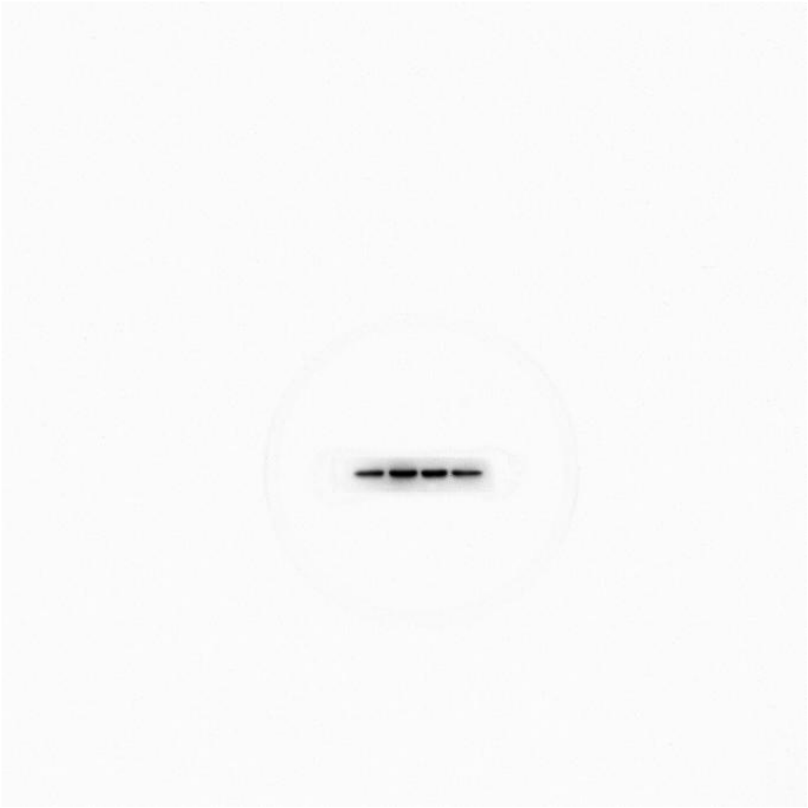

Supplement: Supplementary file 1 [file DataSheet2.PDF]
