# Supplementary material for: Tetrandrine Citrate Suppresses Breast Cancer via Depletion of Glutathione Peroxidase 4 and Activation of Nuclear Receptor Coactivator 4-Mediated Ferritinophagy
Source: Front Pharmacol. 2022 May 9;13:820593. doi: 10.3389/fphar.2022.820593 (PMC9124810; doi:10.3389/fphar.2022.820593)

2E-MCF7-Con

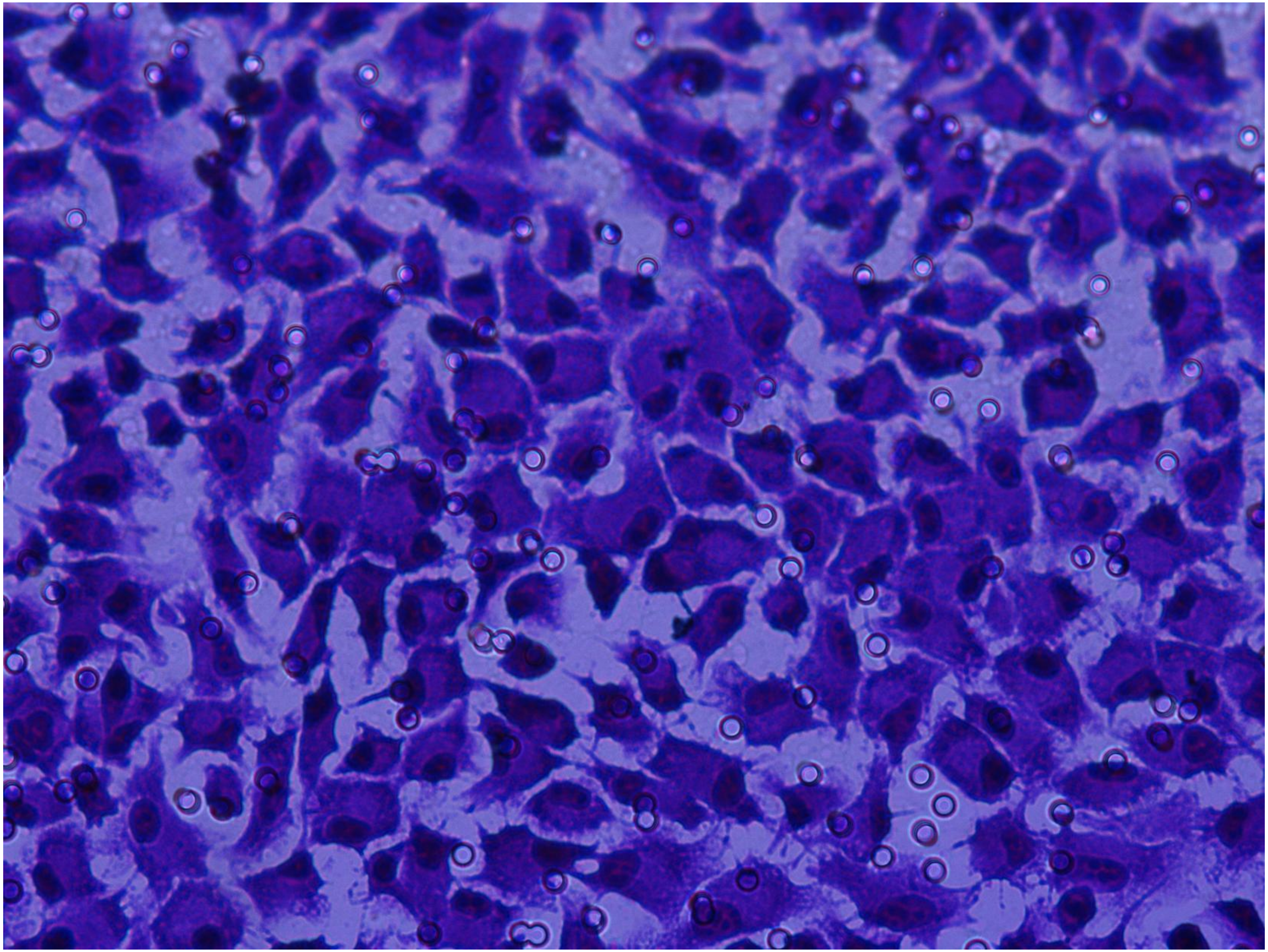

2E-MCF7-TetC

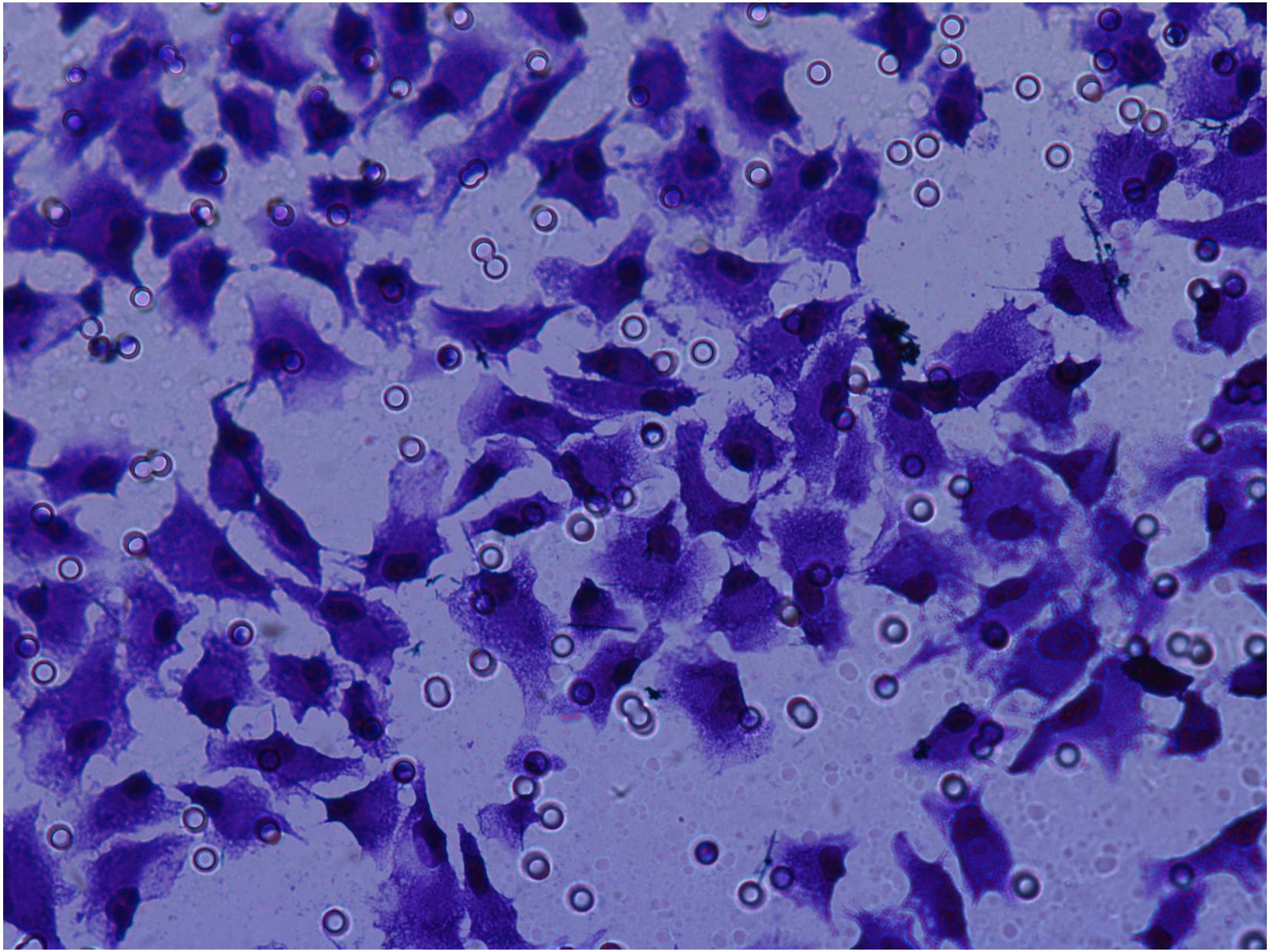

2E-MDA-MB-231-Con

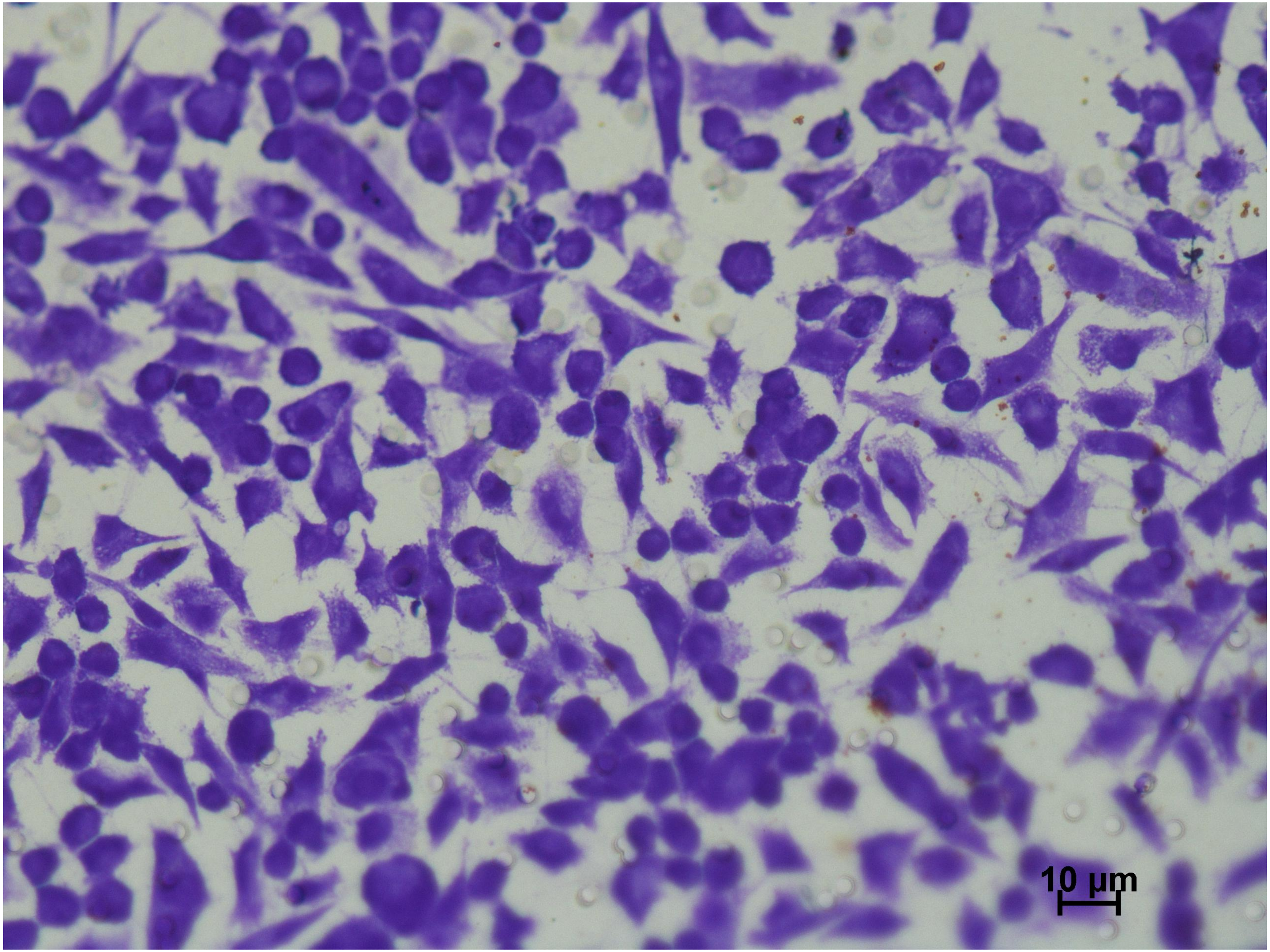

2E-MDA-MB-231-TetC

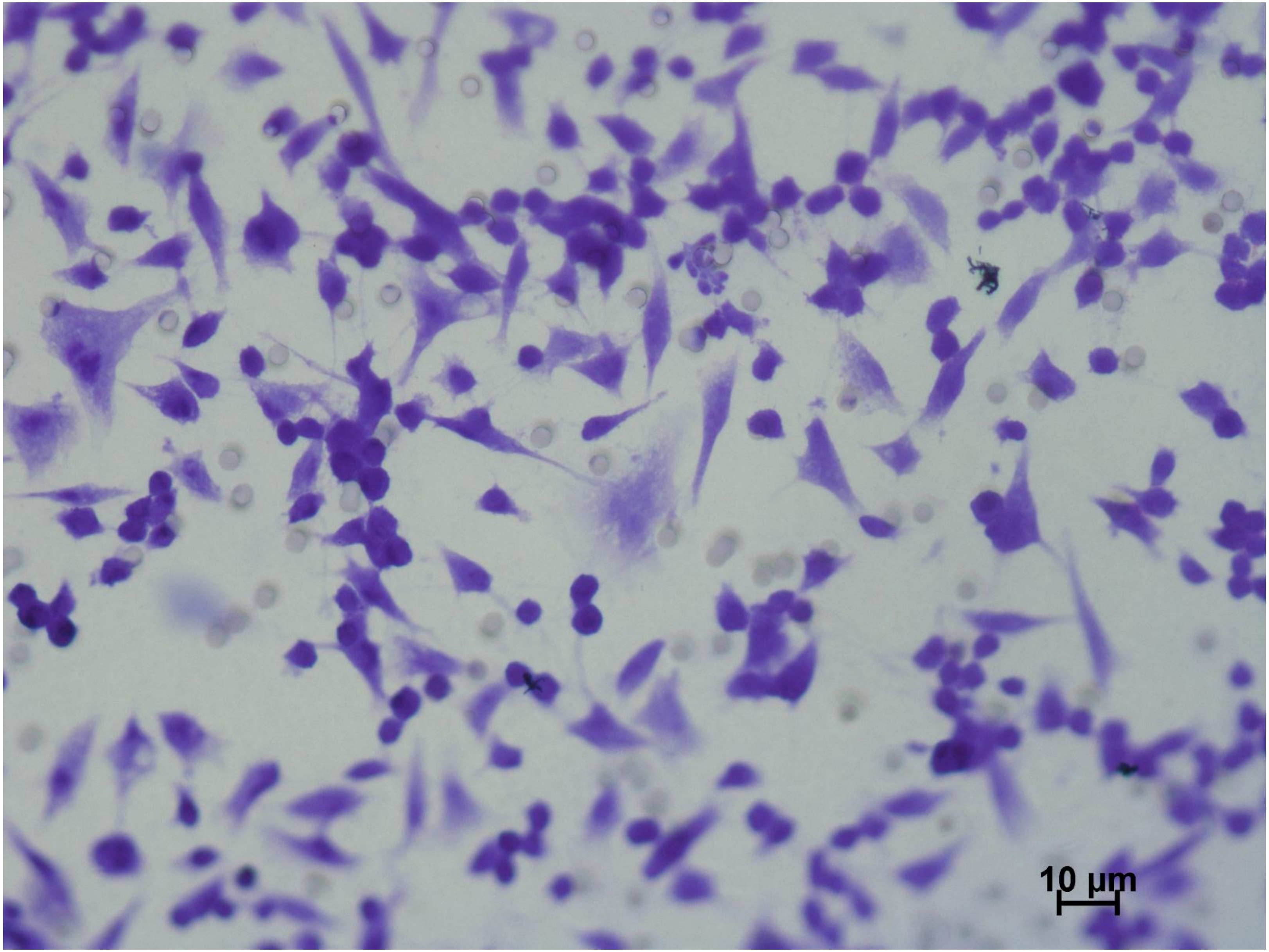

2F-MCF7-Con

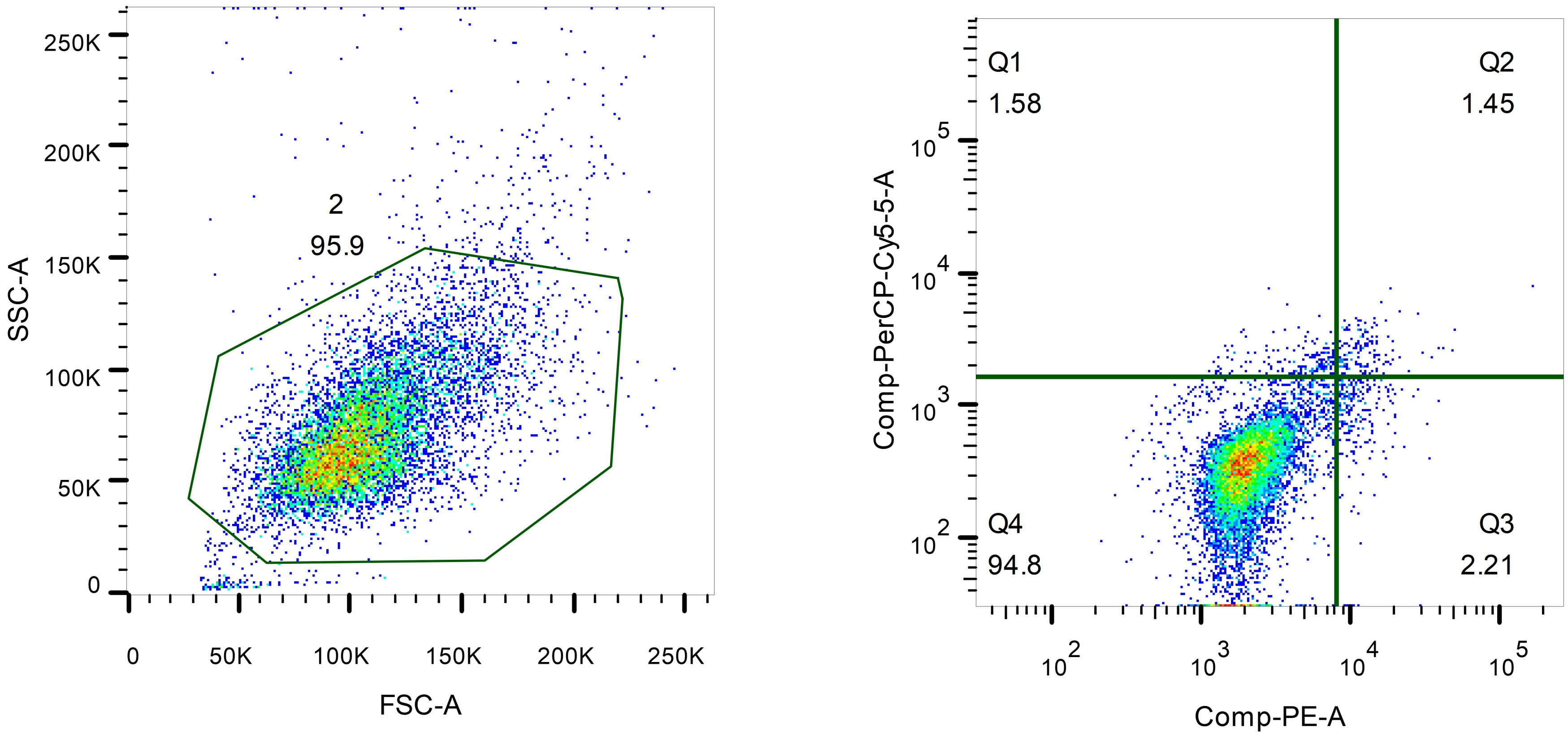

20210922_4_004.fcs
1
11177

20210922_4_004.fcs
2
10724

2F-MCF7-TetC

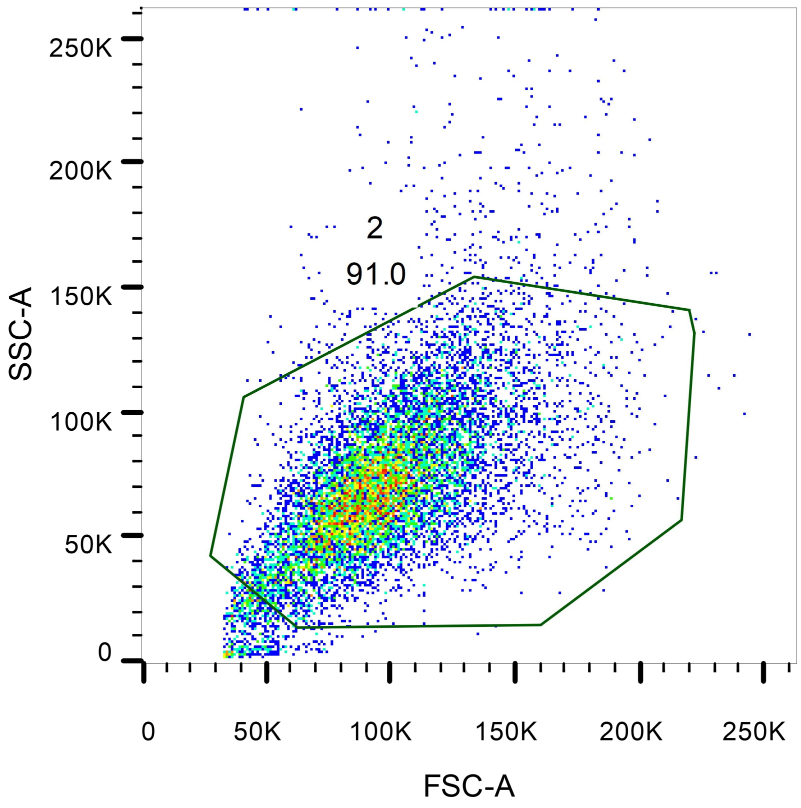

20210922_6_006.fcs
1
11805

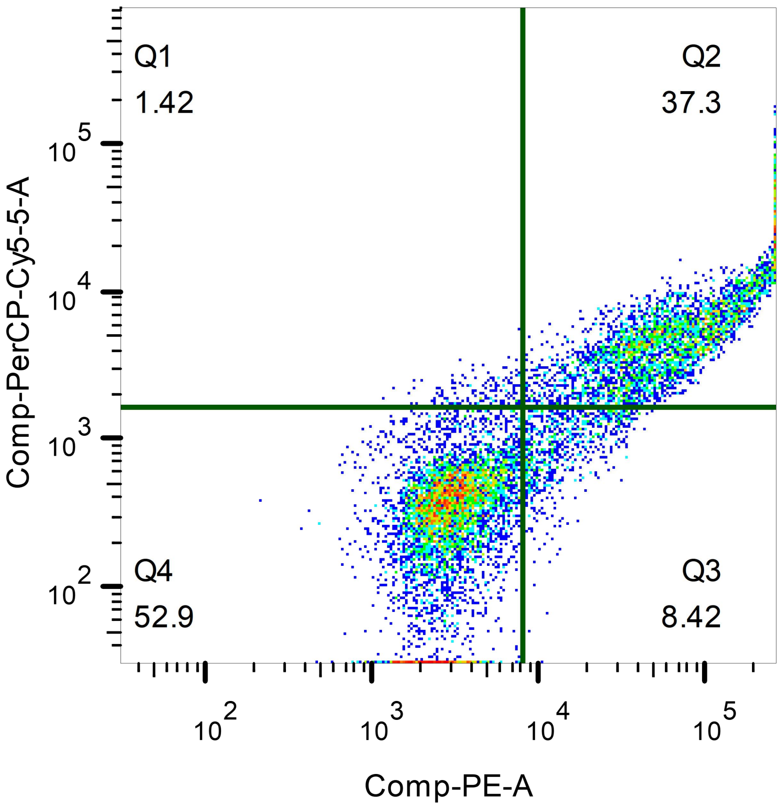

20210922_6_006.fcs
2
10743

2F-MDA-MB-231-Con

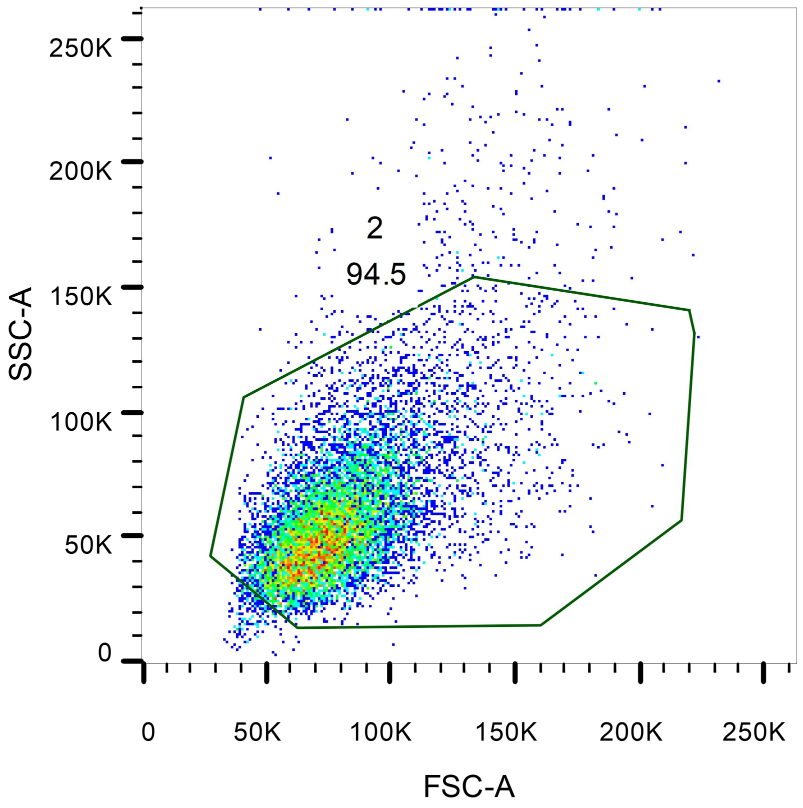

20210922_22_022.fcs
1
10900

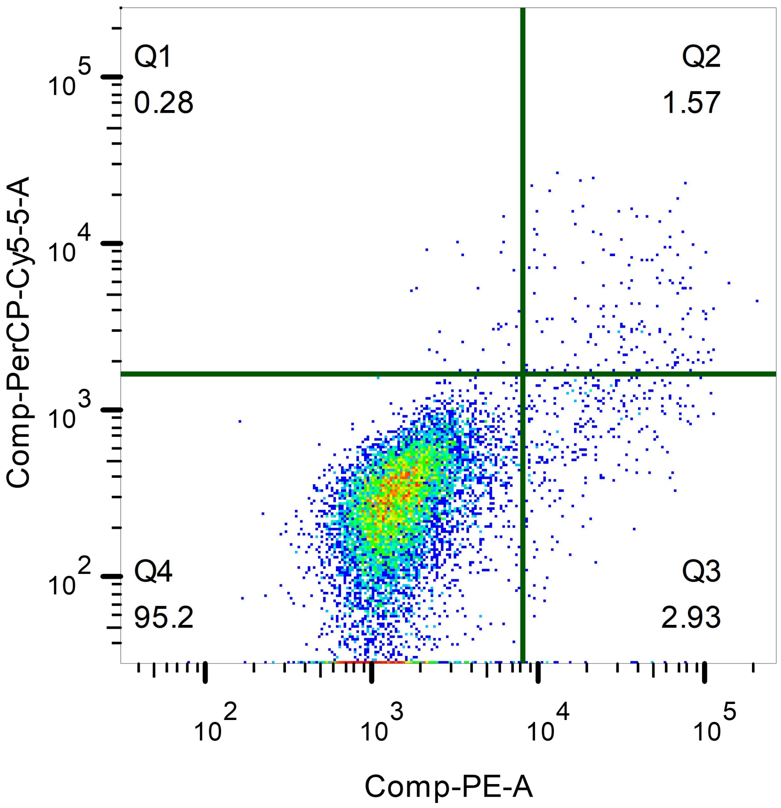

20210922_22_022.fcs
2
10303

2F-MDA-MB-231-TetC

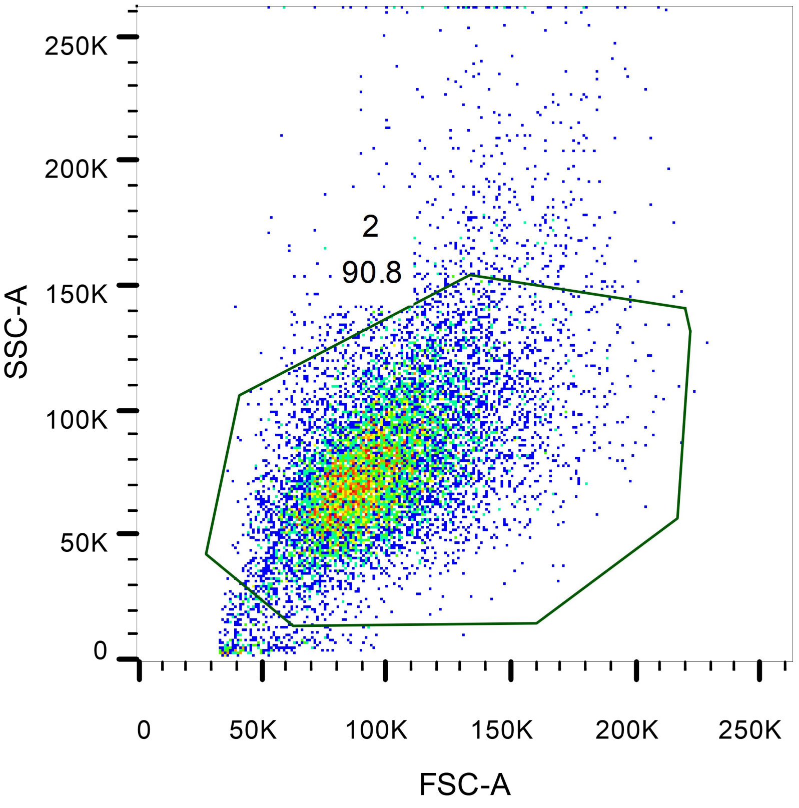

20210922_12_012.fcs
1
11500

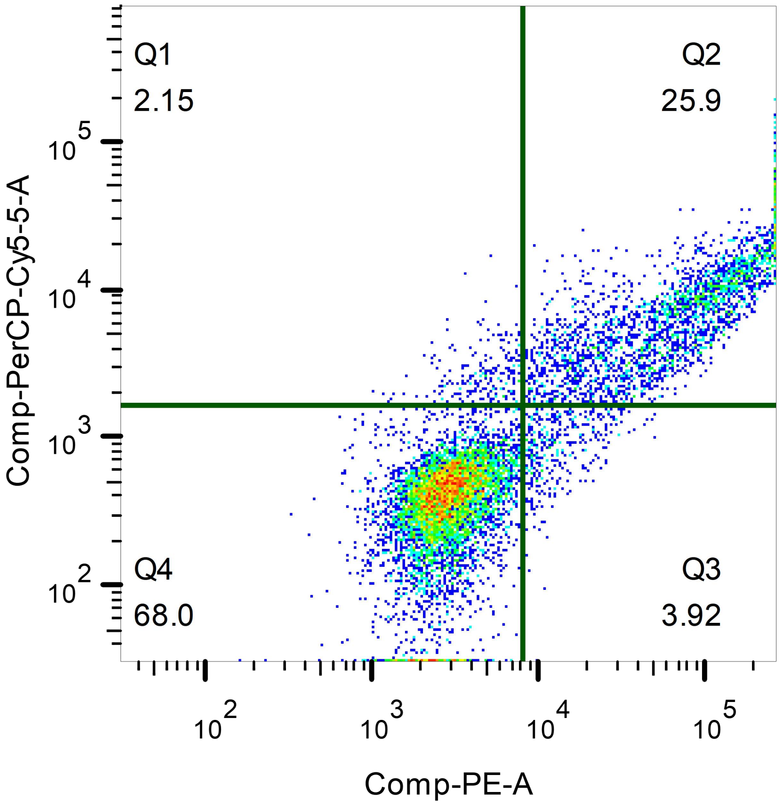

20210922_12_012.fcs
2
10446

4A-MCF7-DMSO

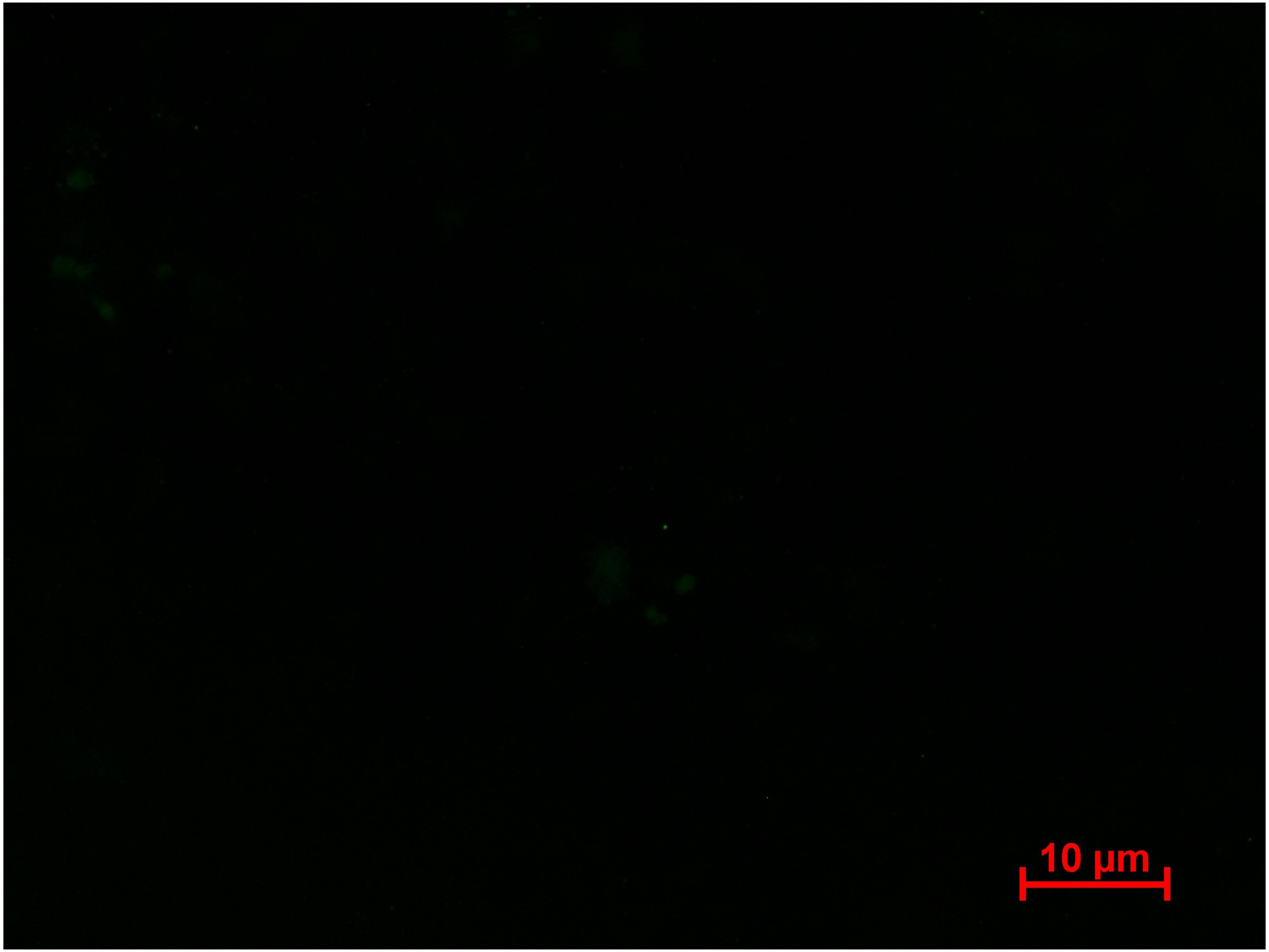

4A-MCF7-Erastin

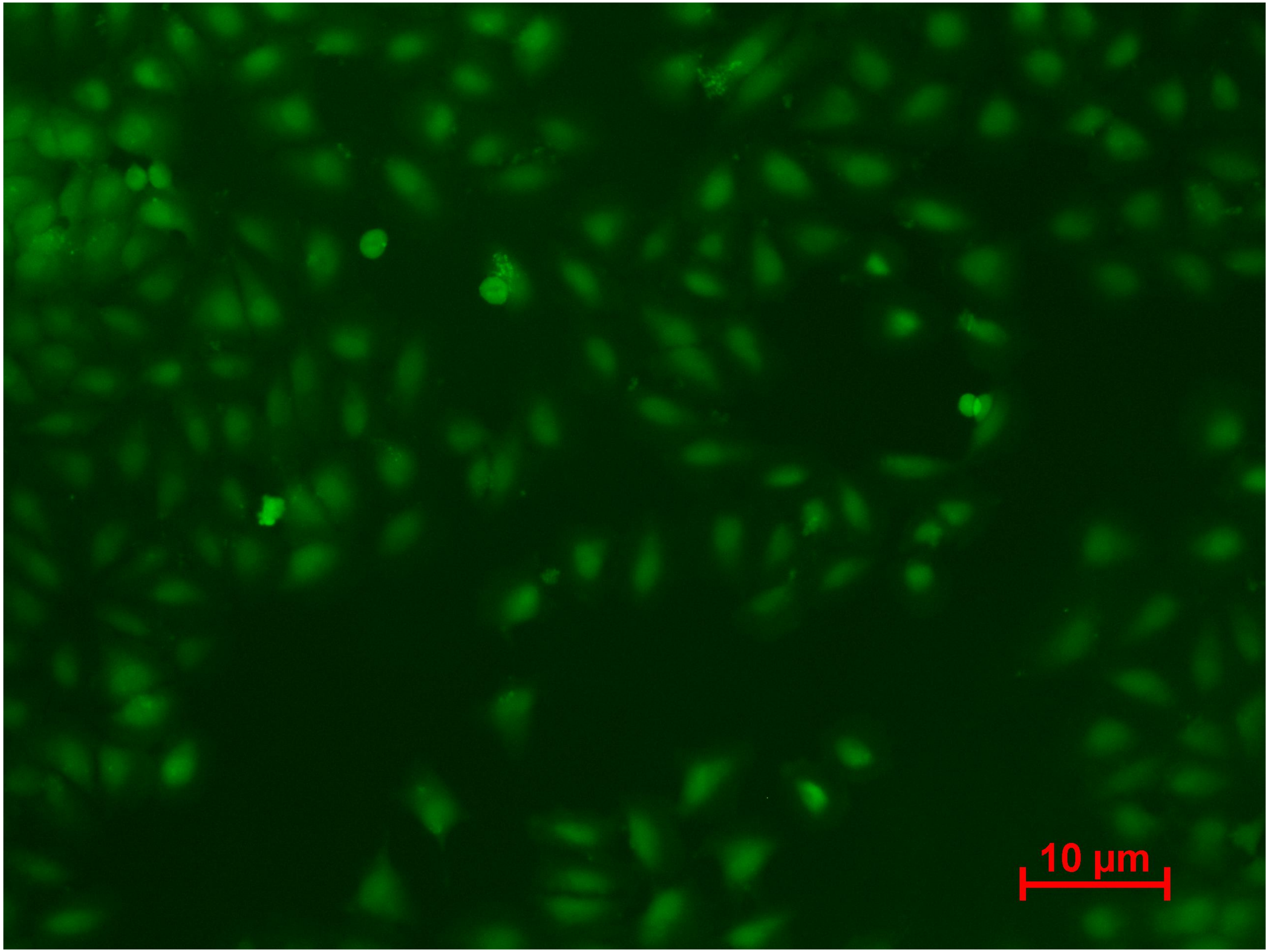

4A-MCF7-TetC+Erastin

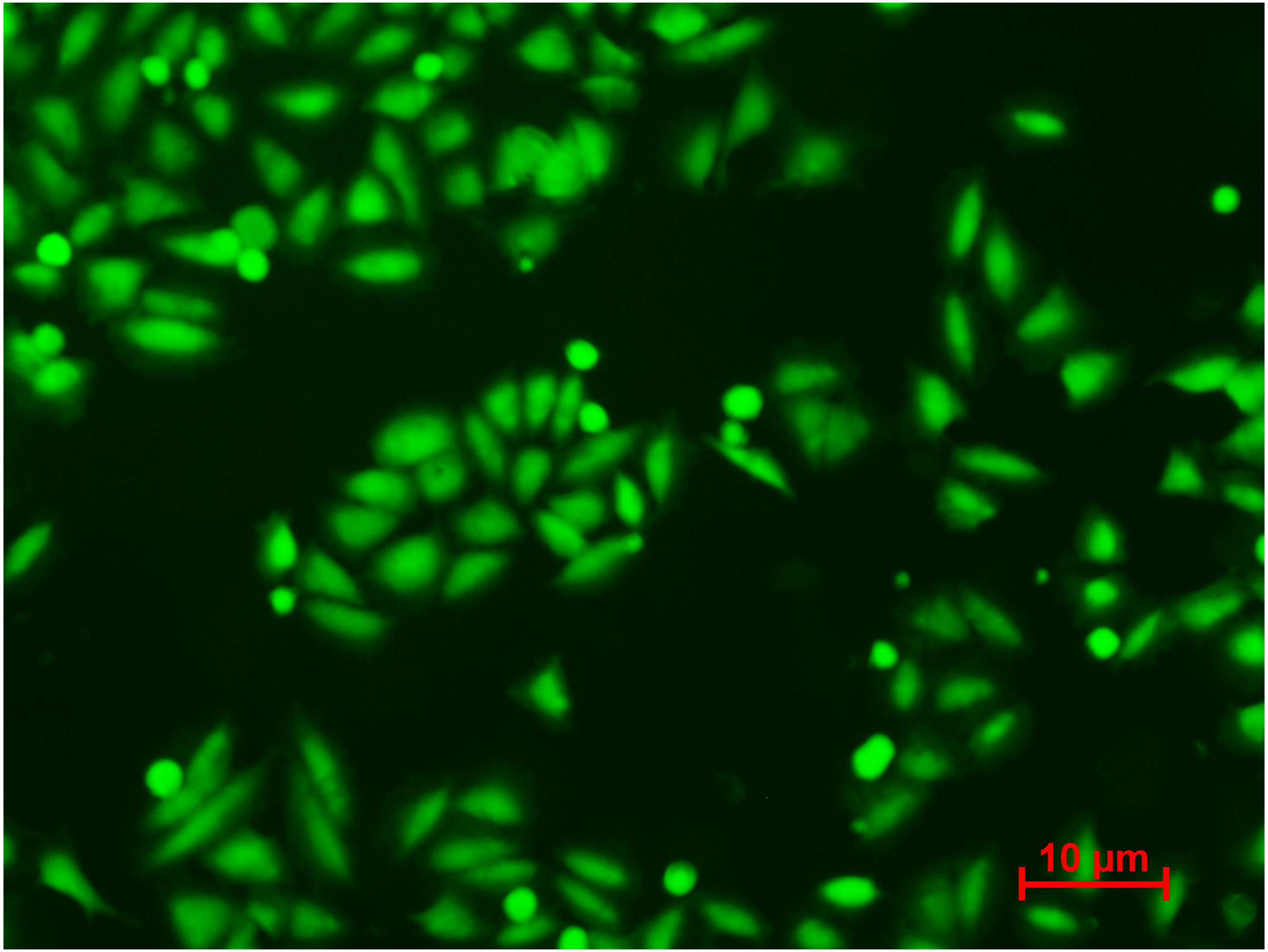

4A-MCF7-RSL3

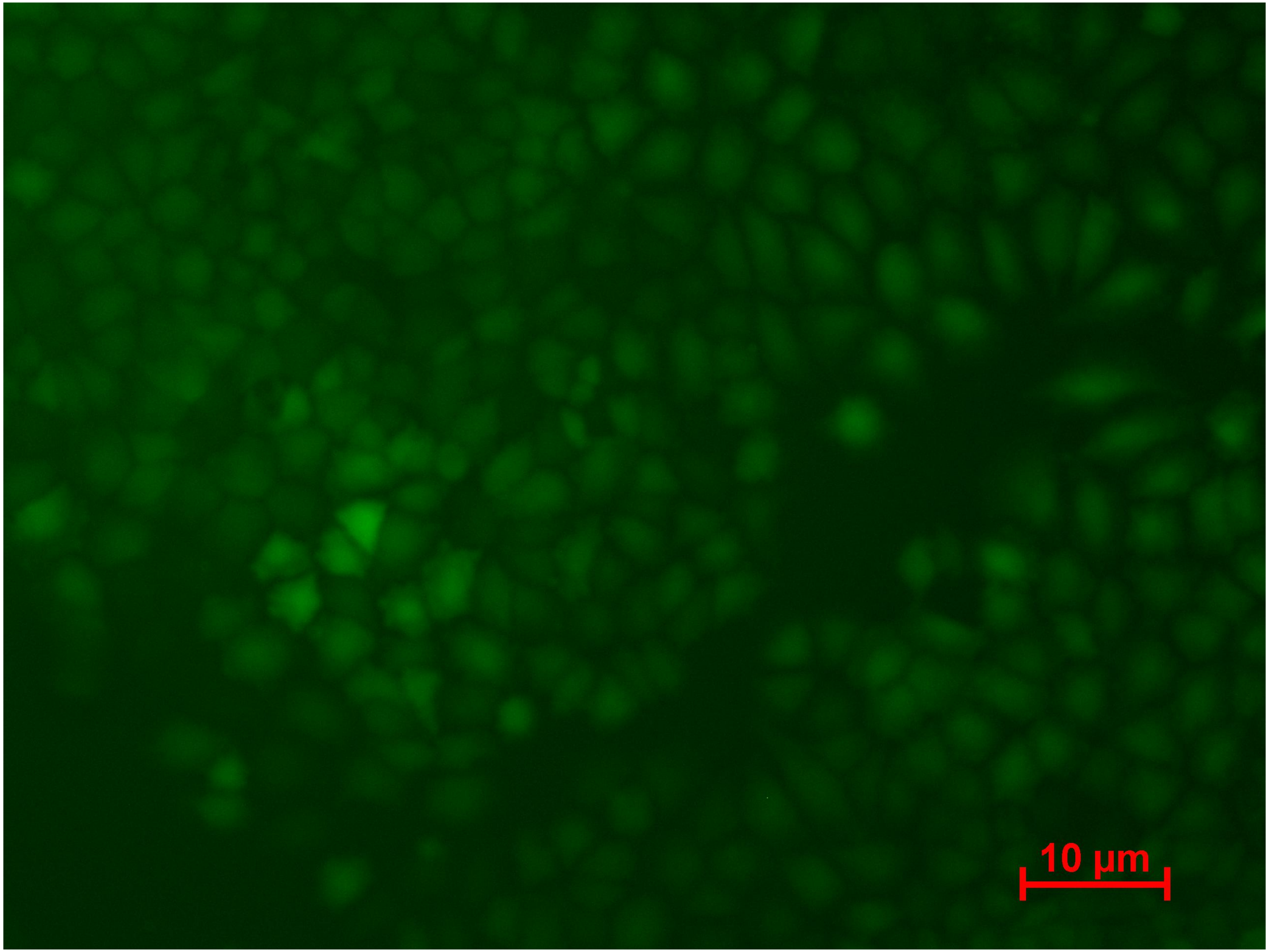

4A-MCF7-TetC+RSL3

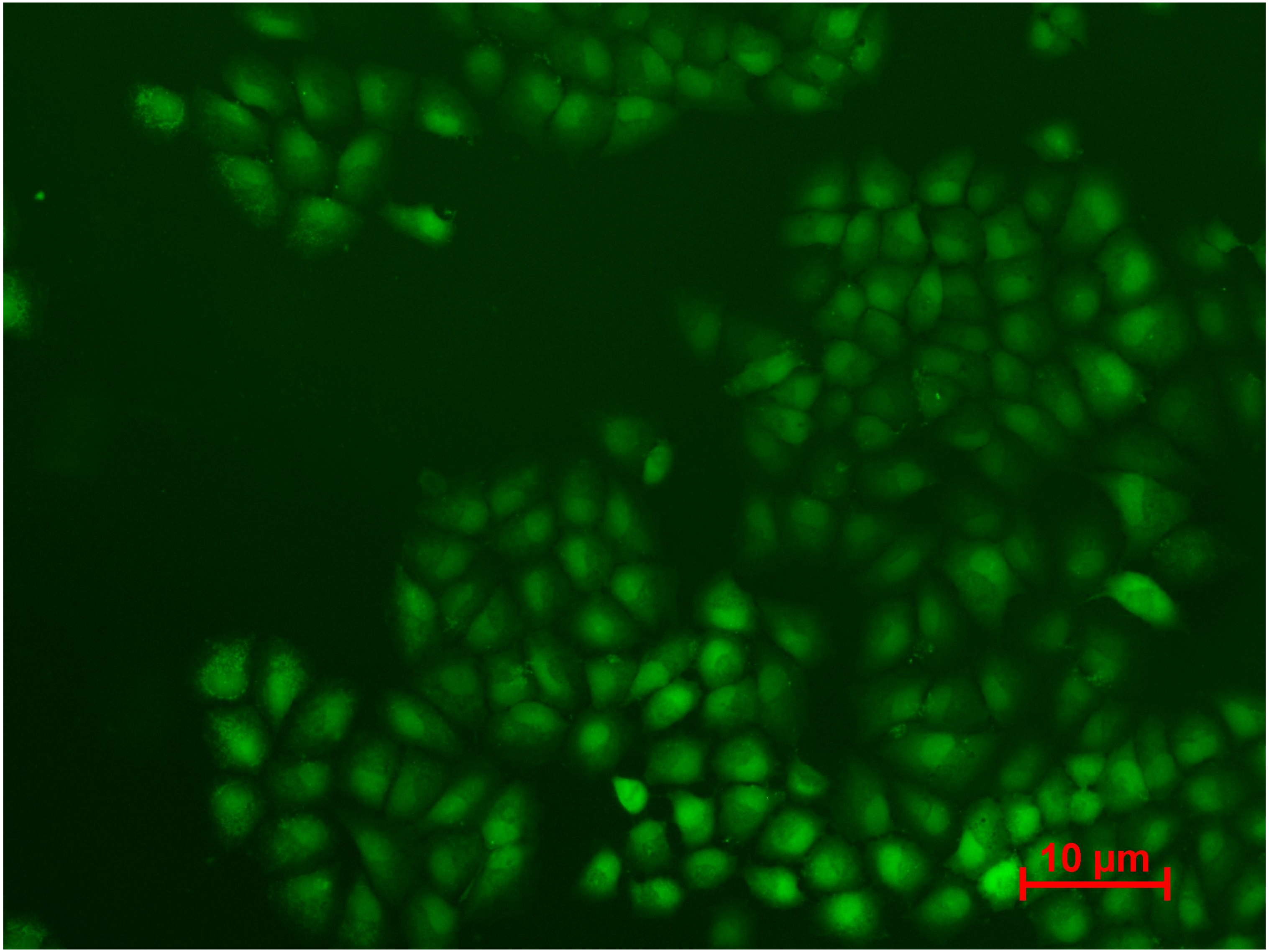

4A-MDA-MB-231-DMSO

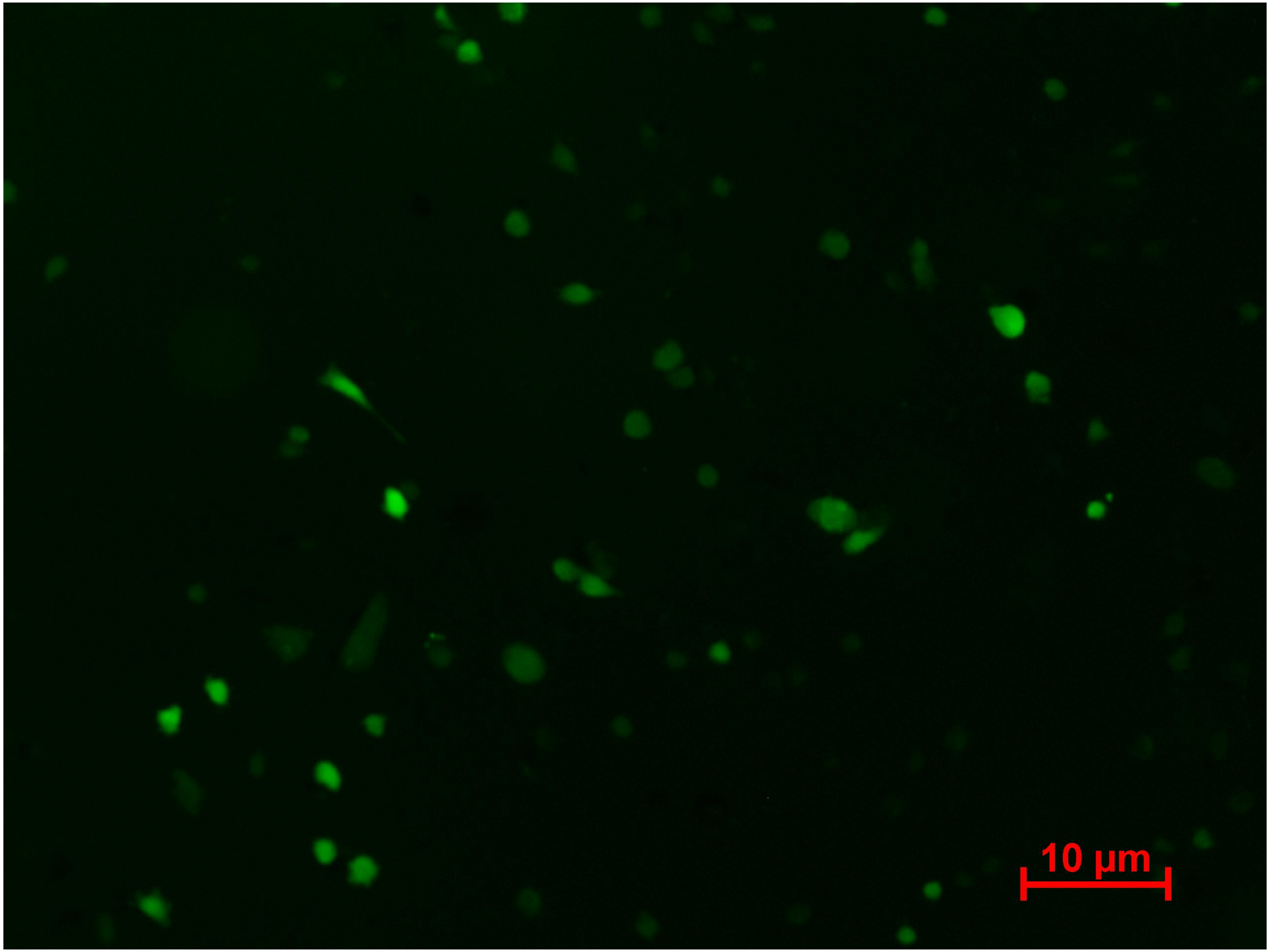

4A-MDA-MB-231-Erastin

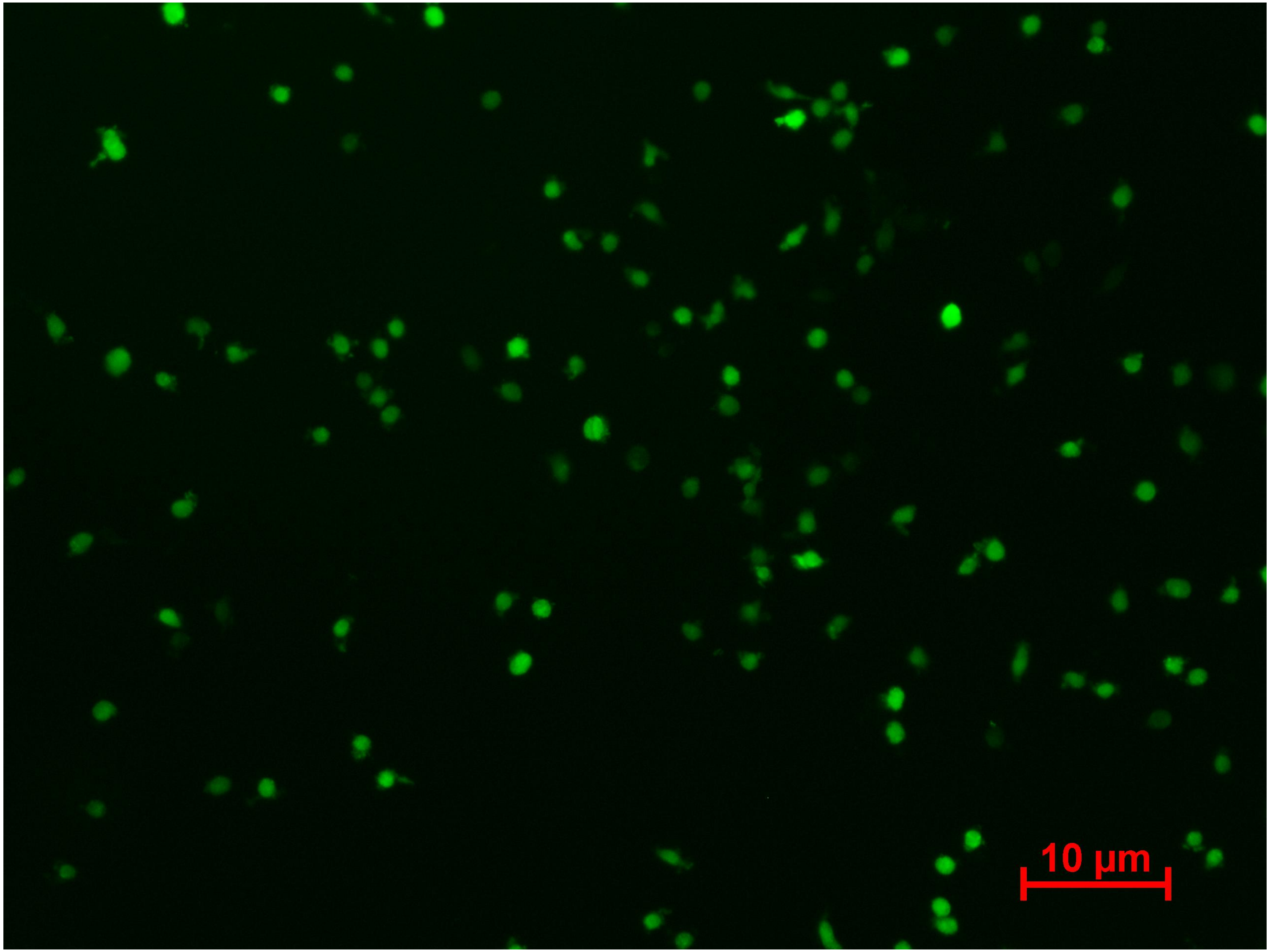

4A-MDA-MB-231-TetC+Erastin

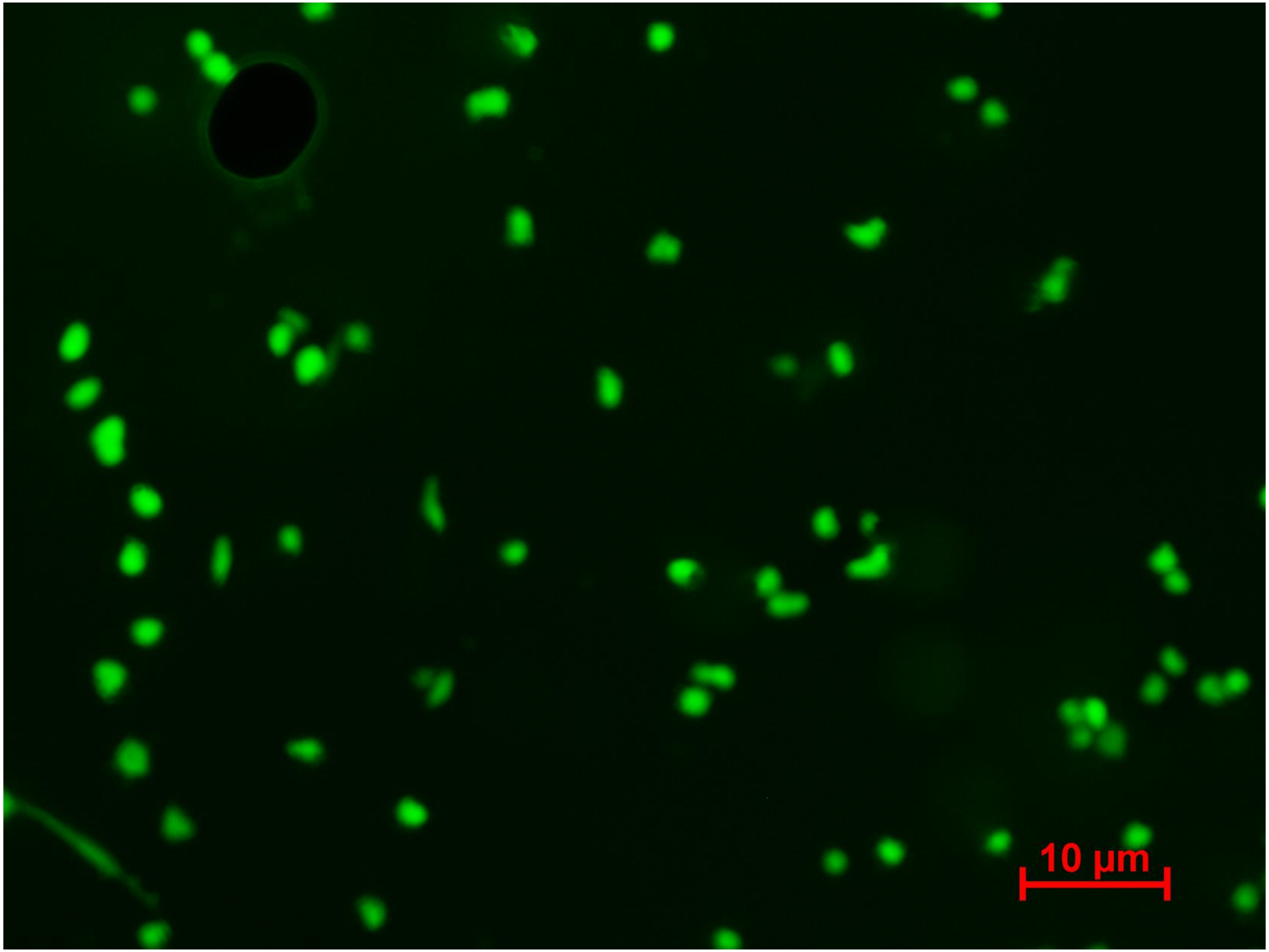

4A-MDA-MB-231-RSL3

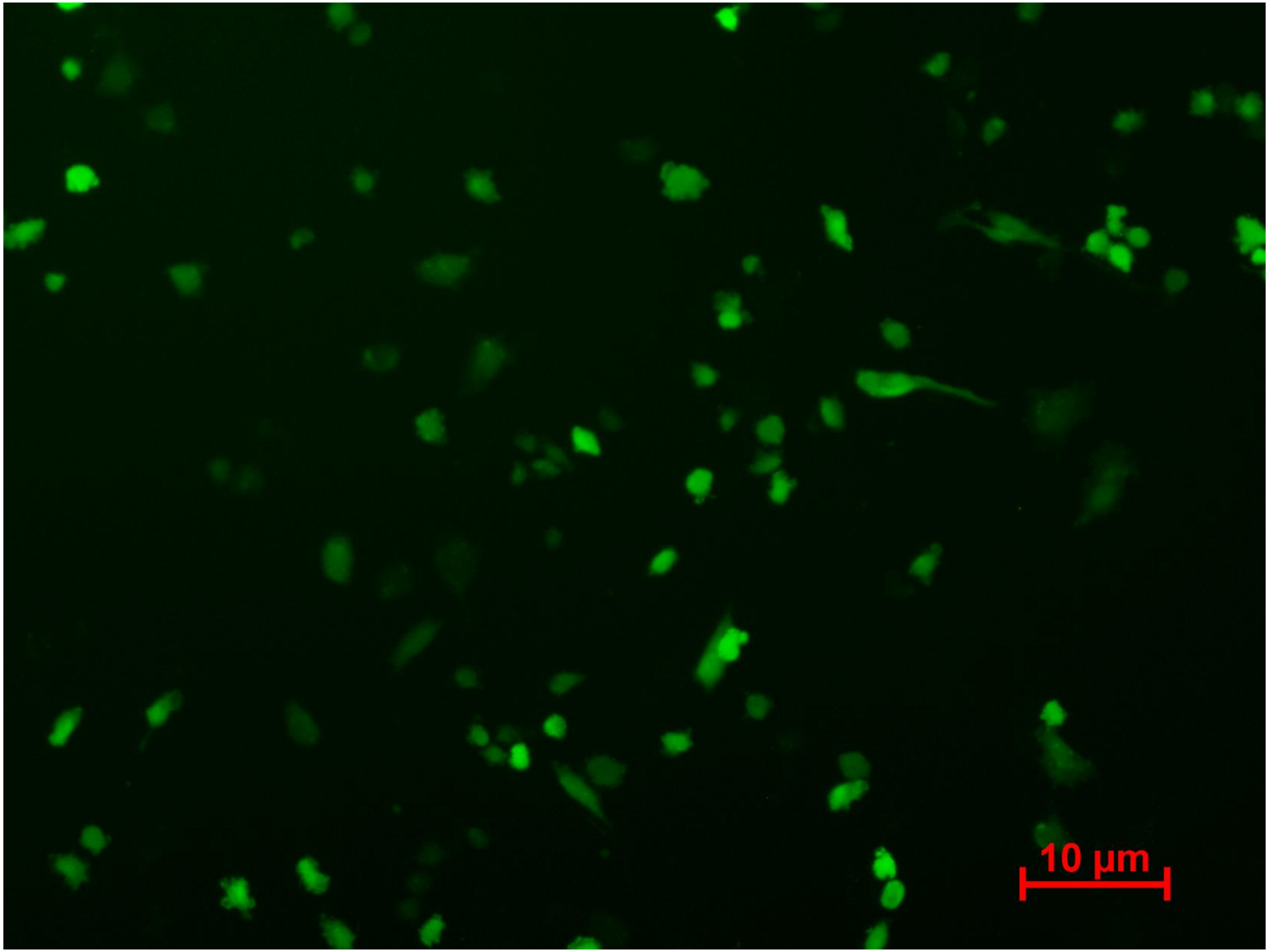

4A-MDA-MB-231-TetC+RSL3

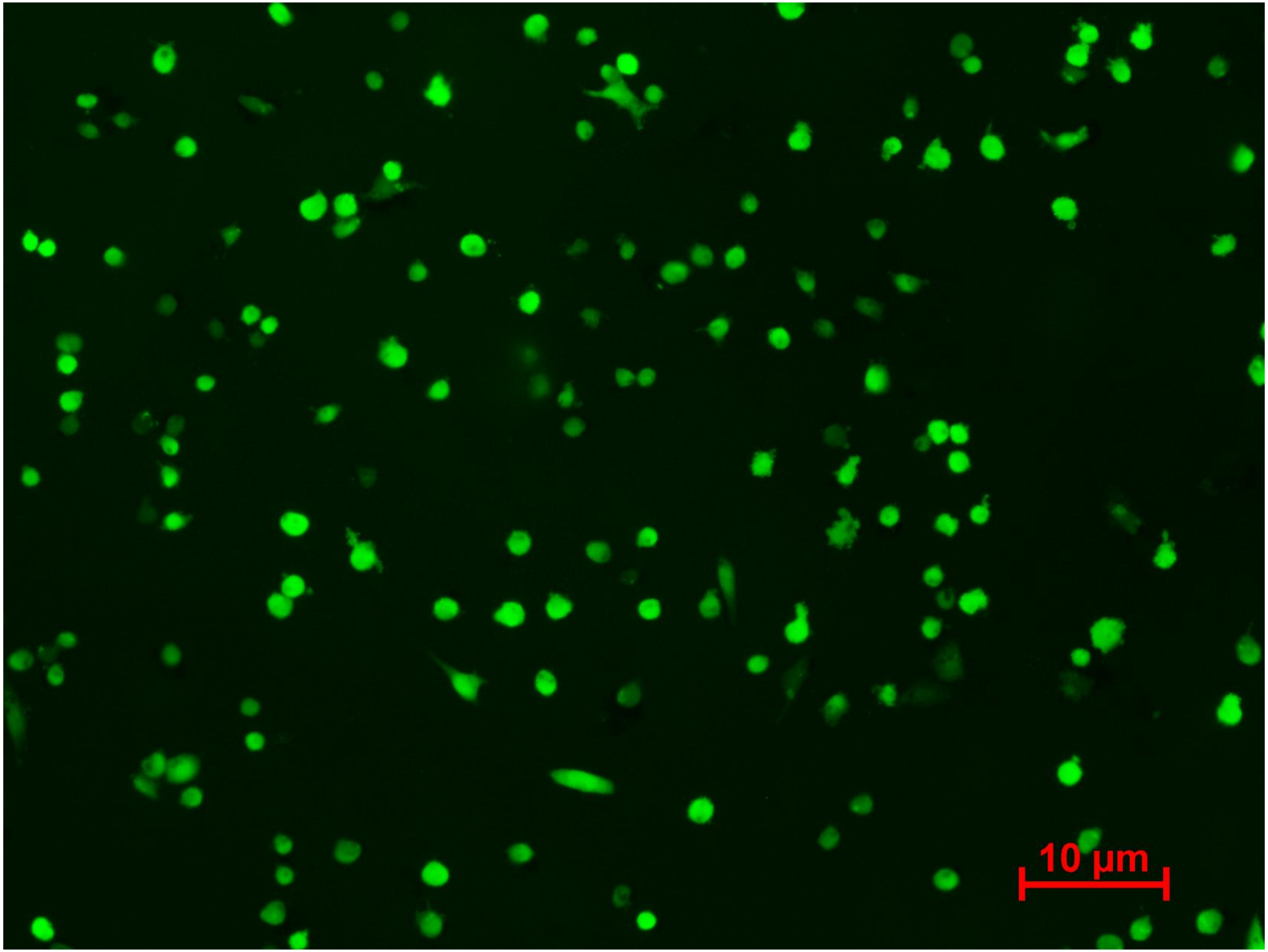

4D-MCF7-DMSO

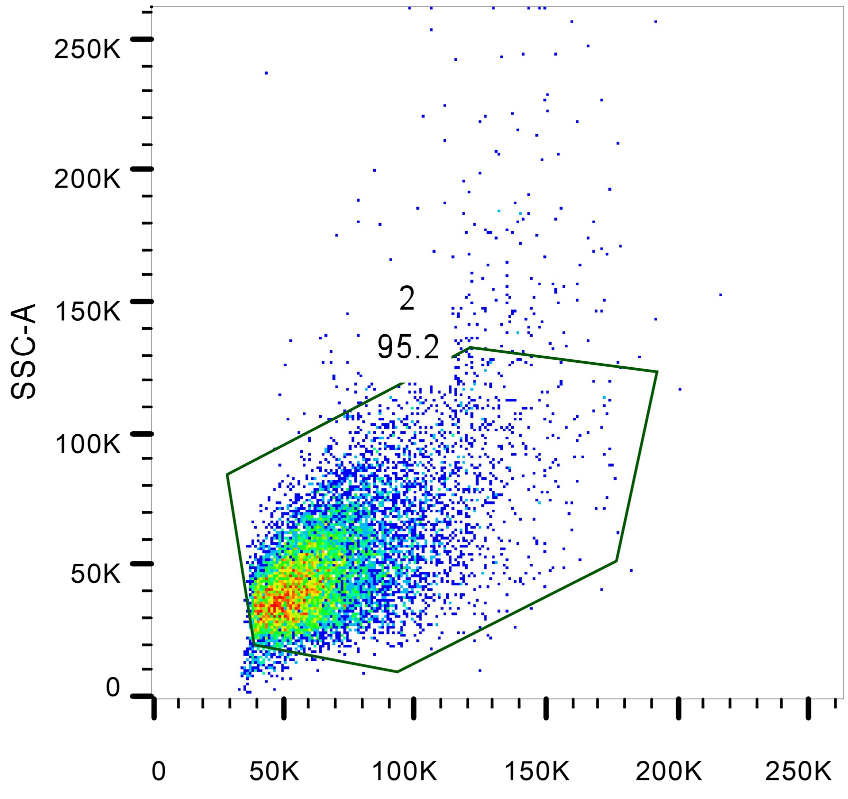

20210917_18_018.fcs
1
11199

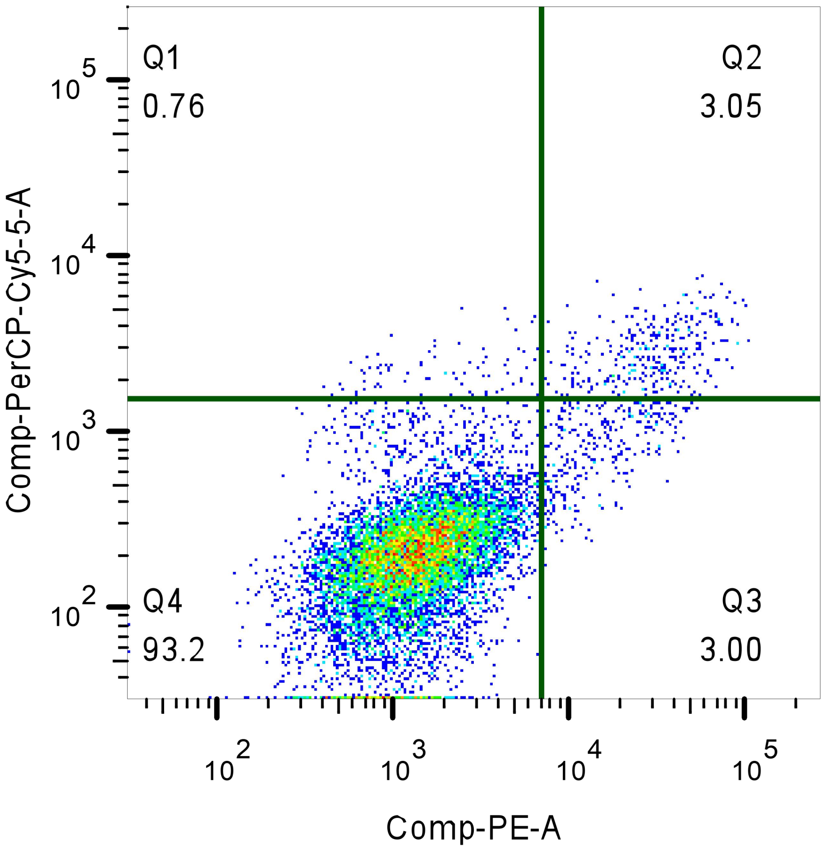

20210917_18_018.fcs
2
10664

4D-MCF7-Erastin

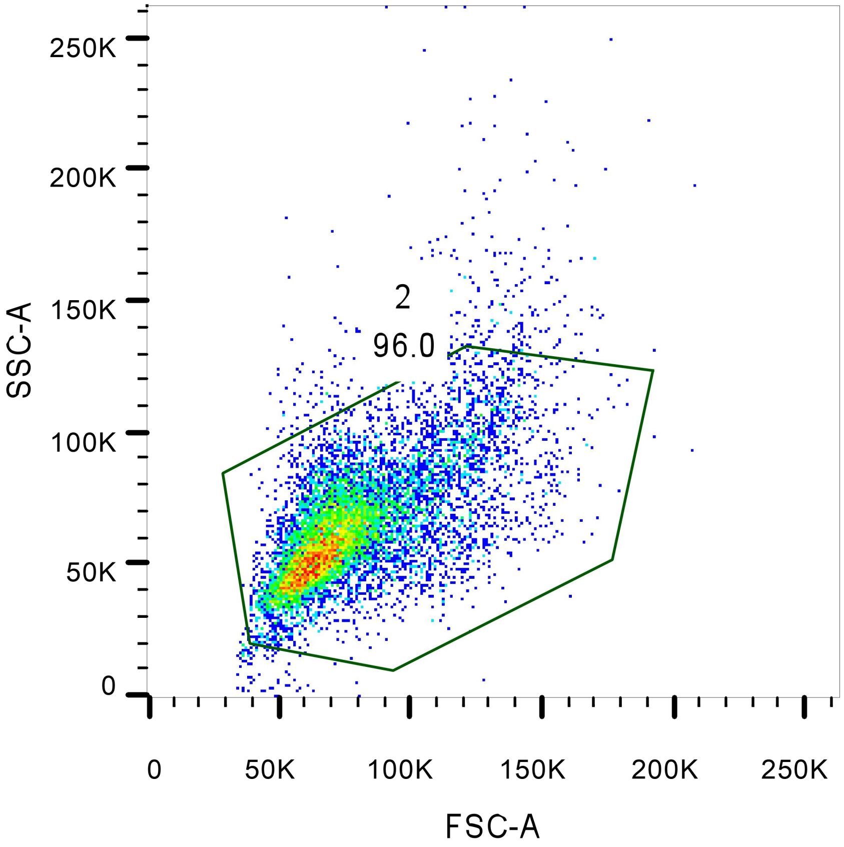

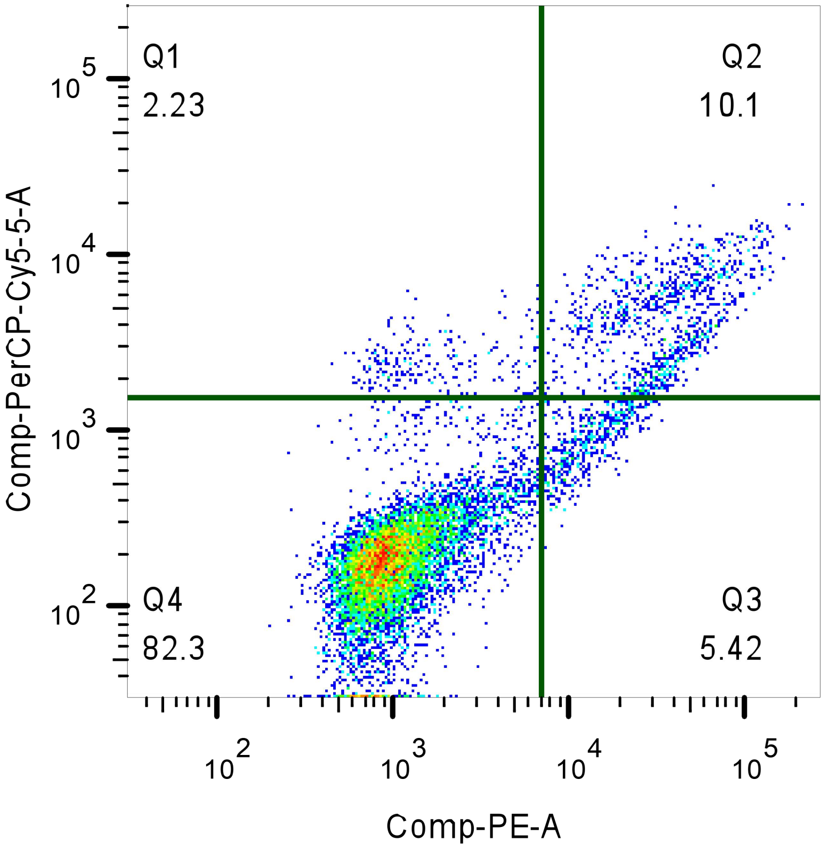

20210917_6_006.fcs
1
10566

20210917_6_006.fcs
2
10145

4D-MCF7-TetC+Erastin

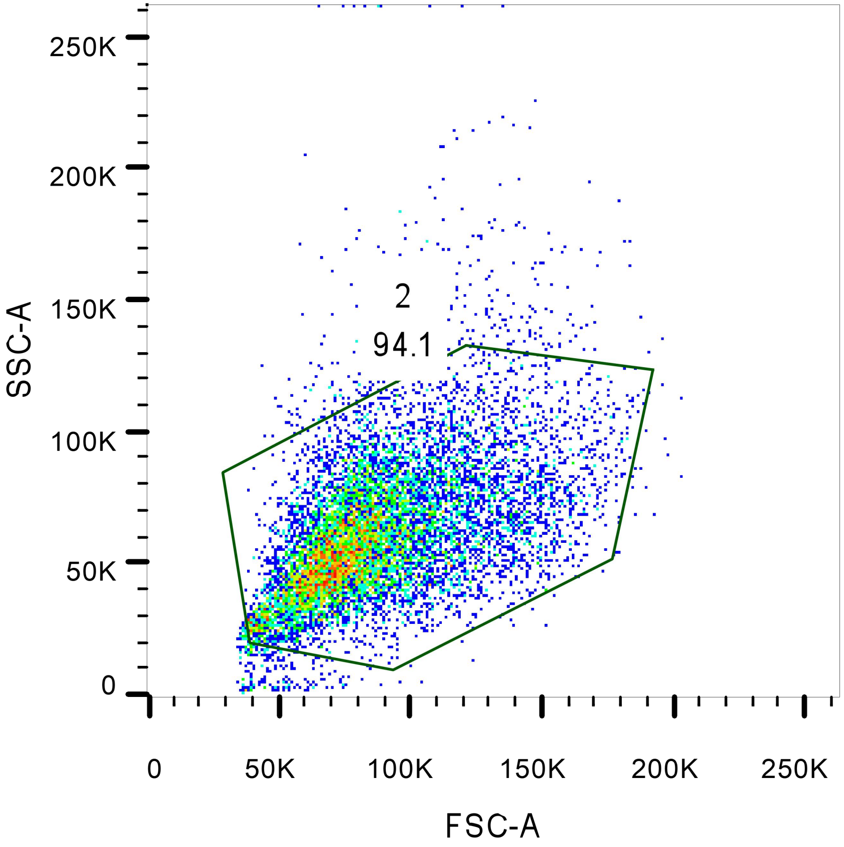

20210917_5_005.fcs
1
10801

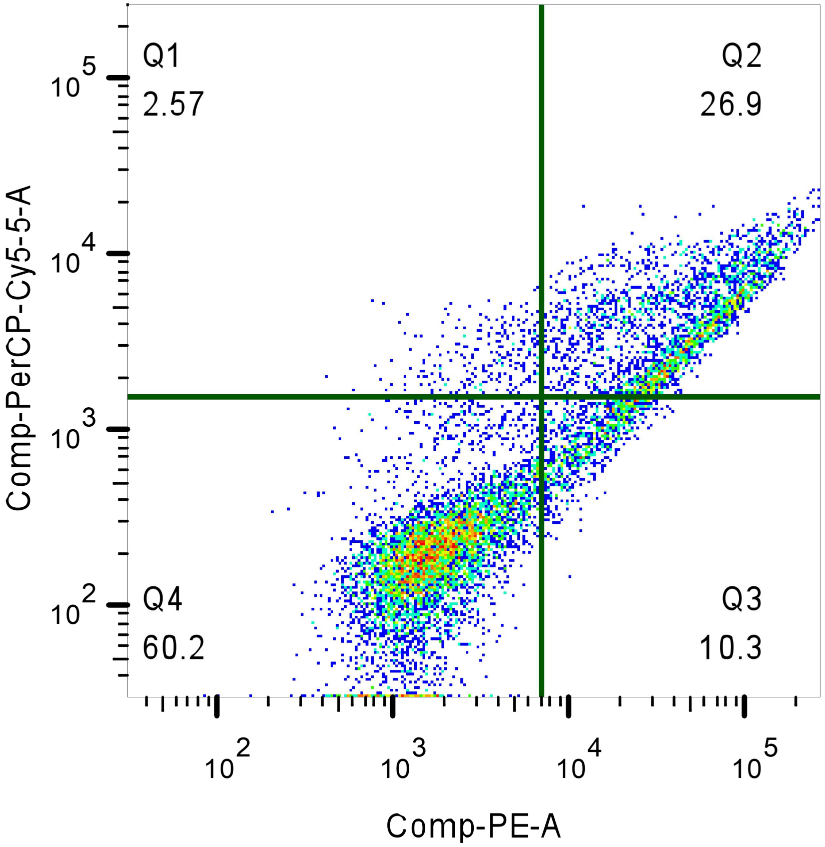

20210917_5_005.fcs
2
10162

4D-MCF7-RSL3

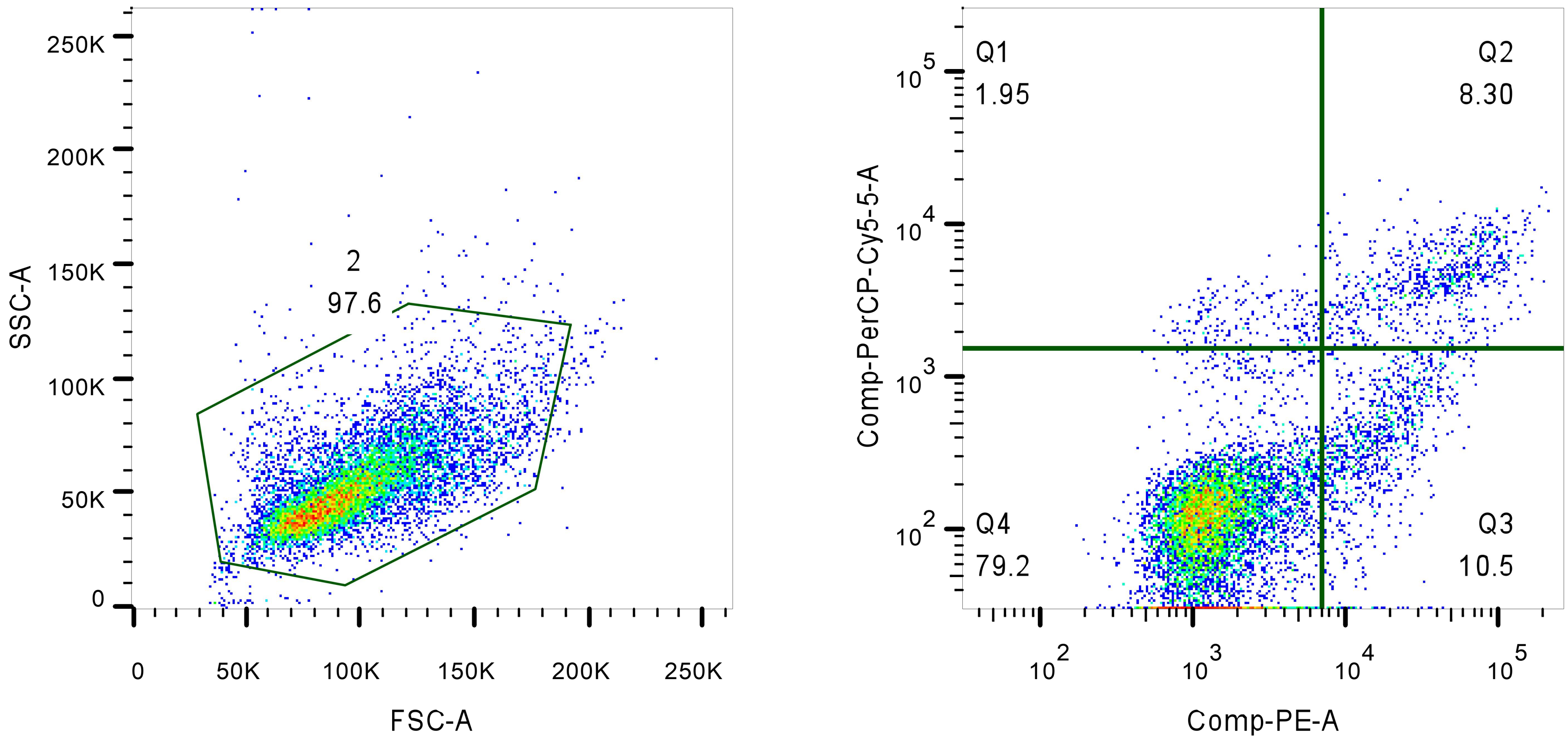

20210917_9_009.fcs
1
10220

20210917_9_009.fcs
2
9975

4D-MCF7-TetC+RSL3

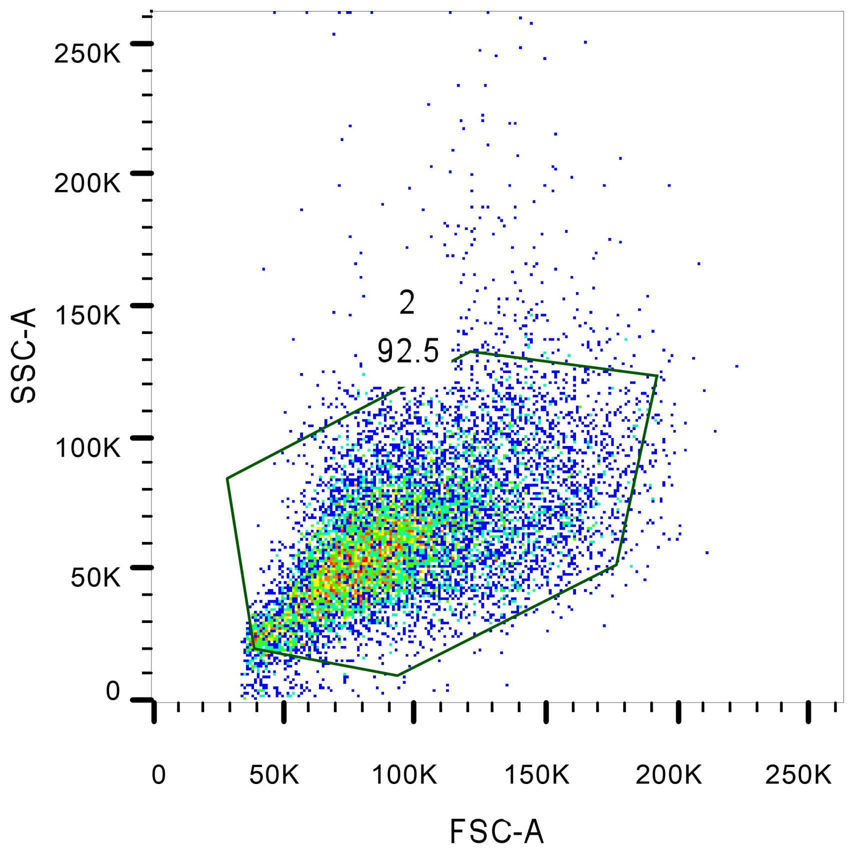

20210917_10_010.fcs
1
11078

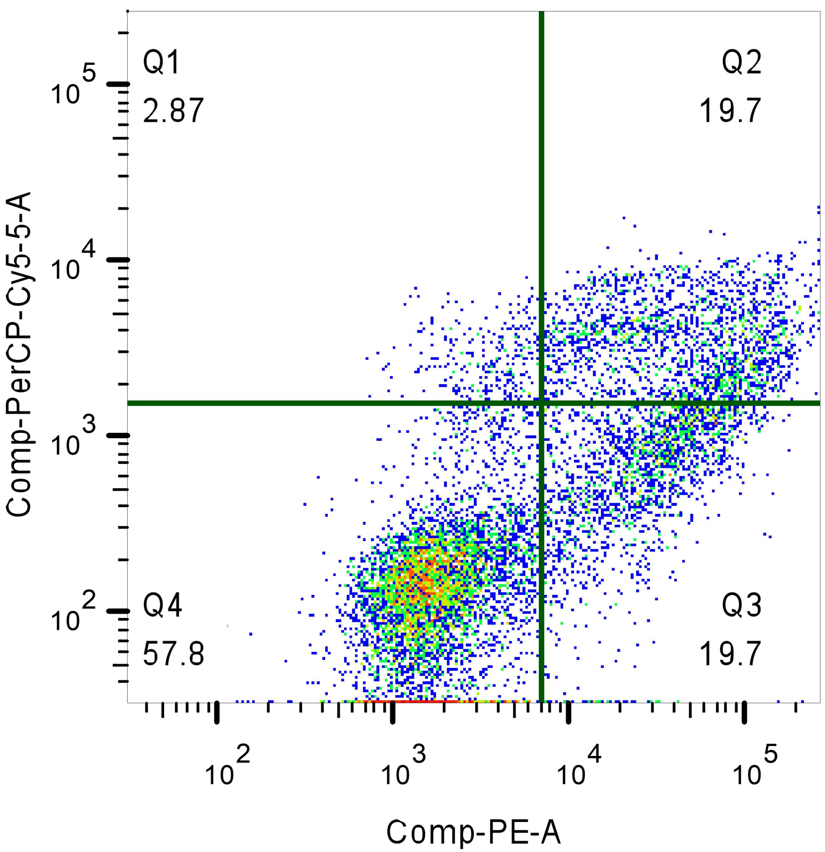

20210917_10_010.fcs
2
10248

4D-MDA-MB-231-DMSO

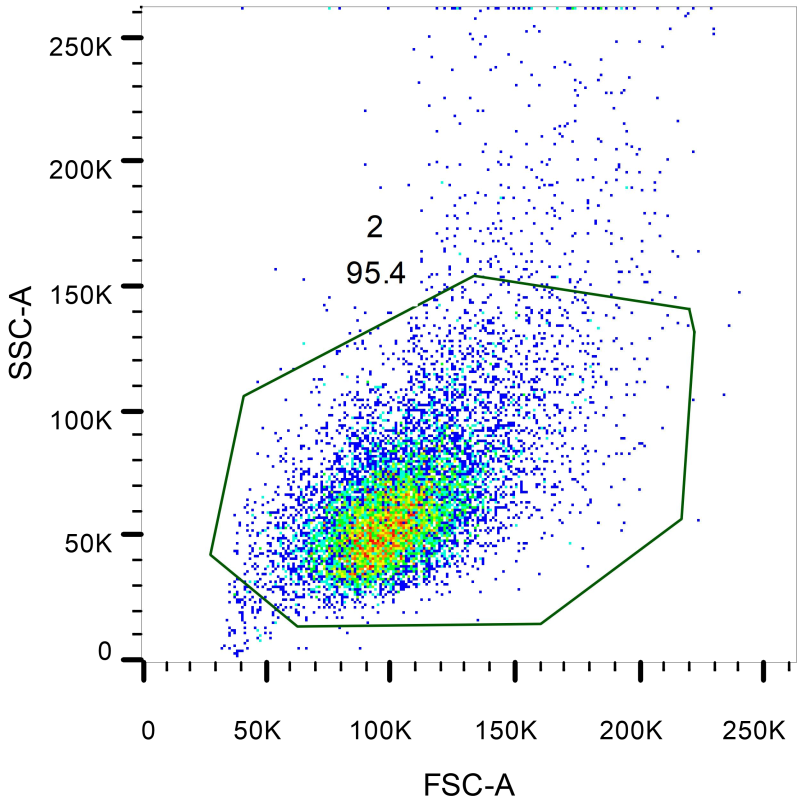

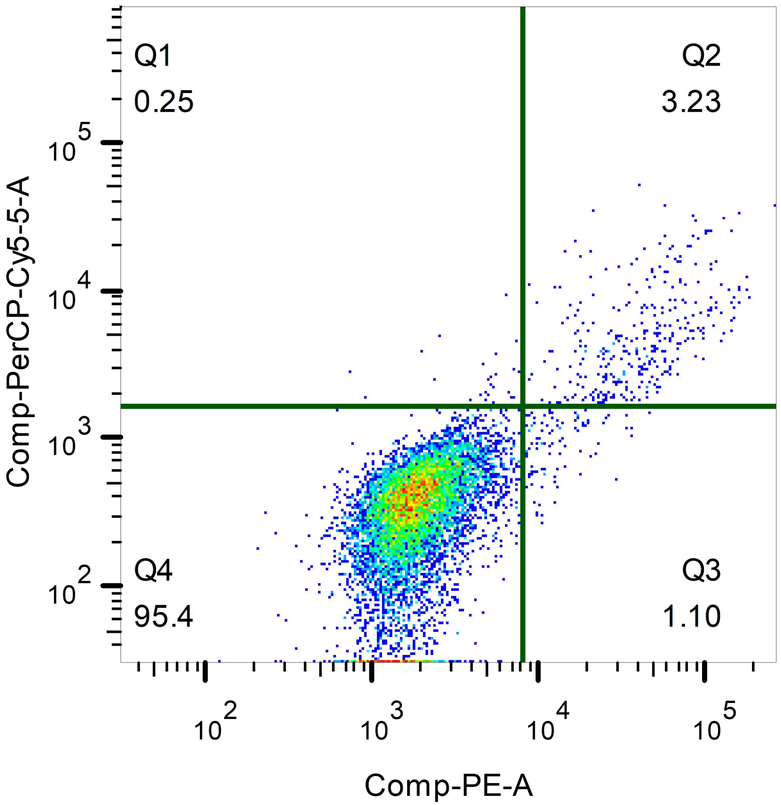

20210922_19_019.fcs
1
10707

20210922_19_019.fcs
2
10218

4D-MDA-MB-231-Erastin

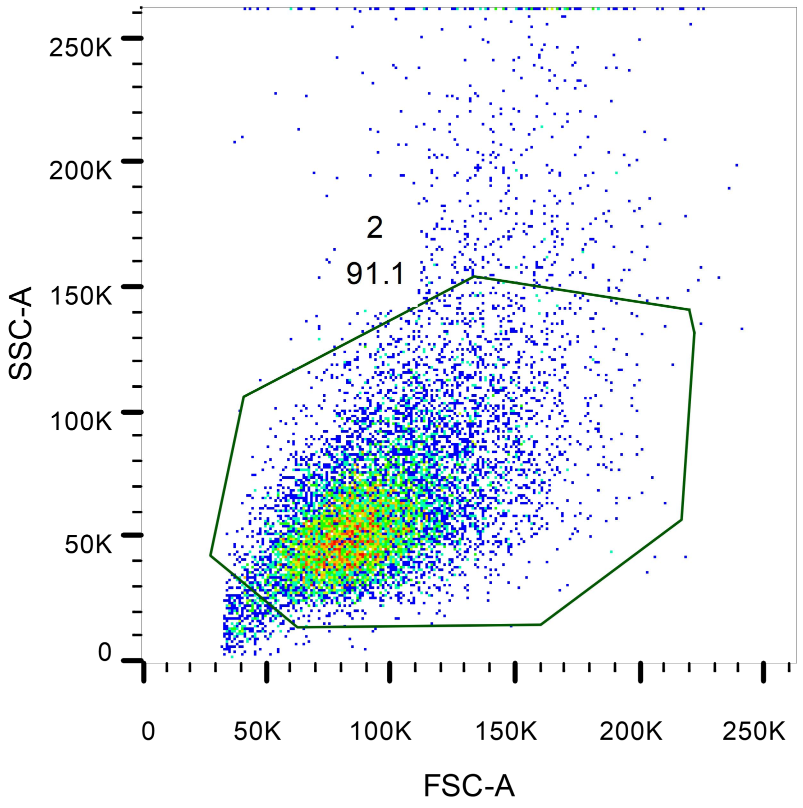

20210922_25_025.fcs
1
11356

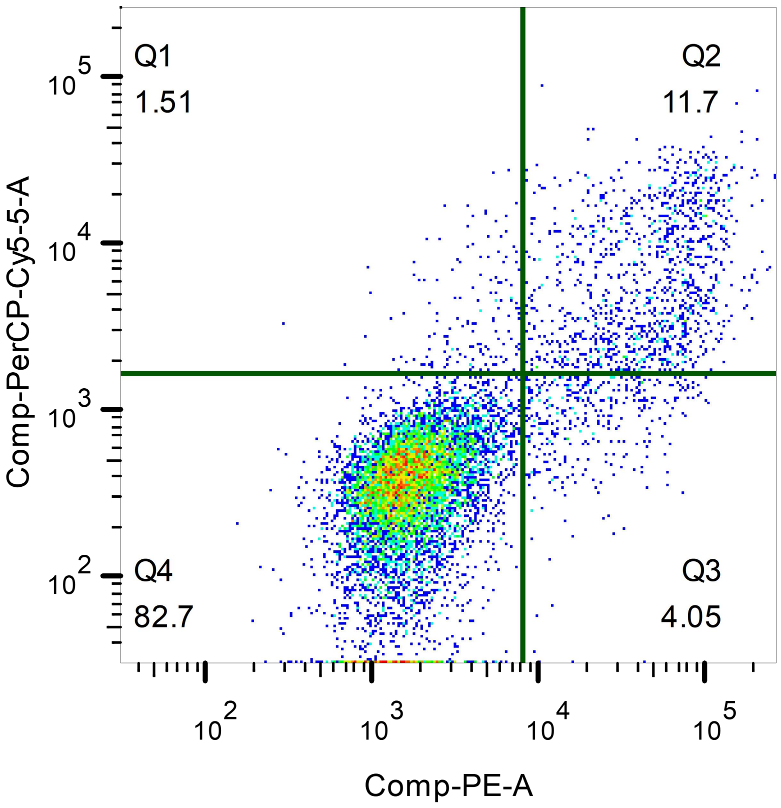

20210922_25_025.fcs
2
10348

4D-MDA-MB-231-TetC+Erastin

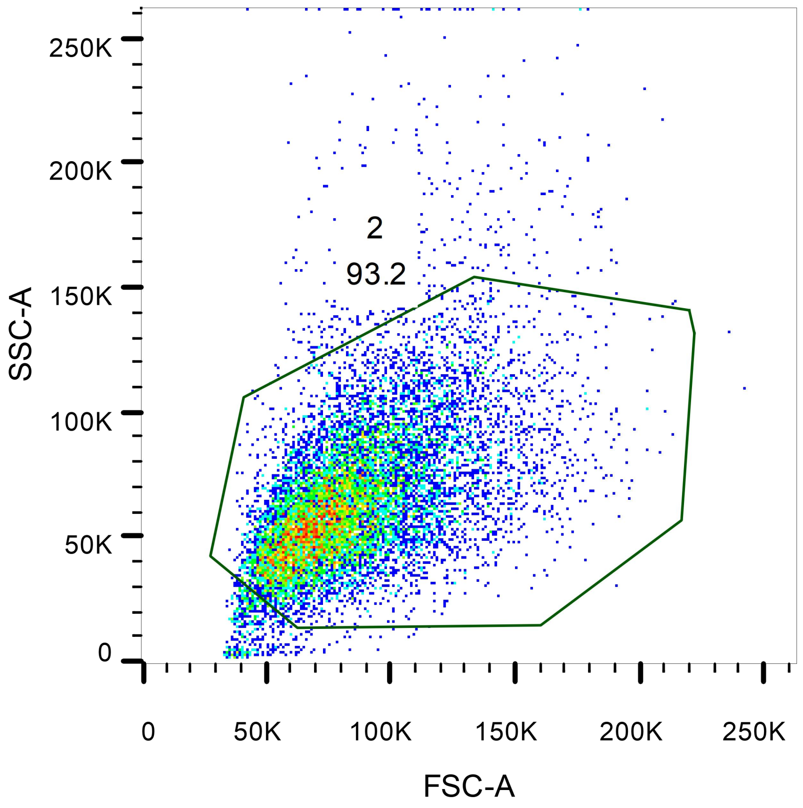

20210922_14_014.fcs
1
12722

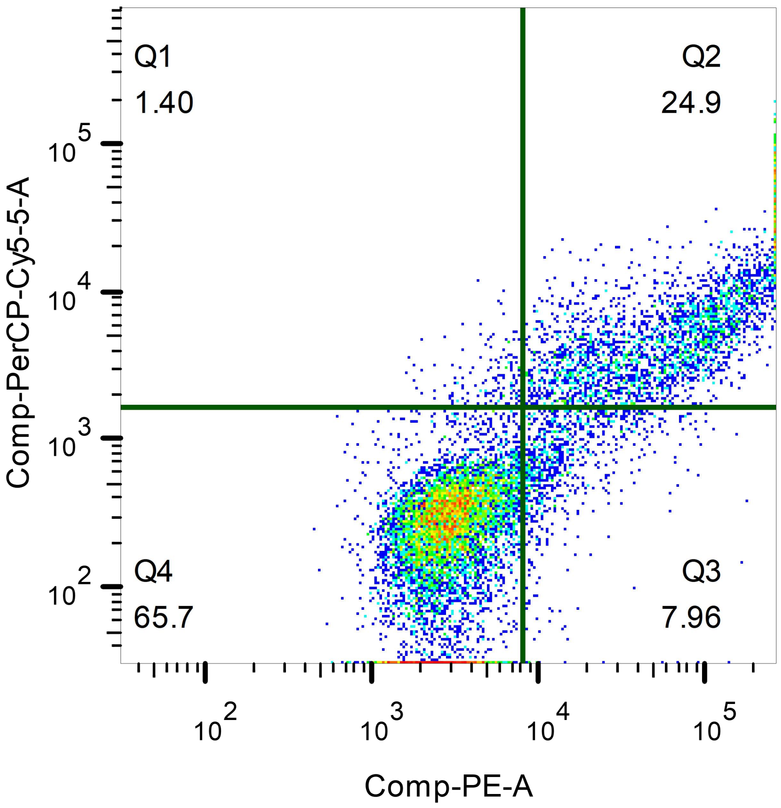

20210922_14_014.fcs
2
11857

4D-MDA-MB-231-RSL3

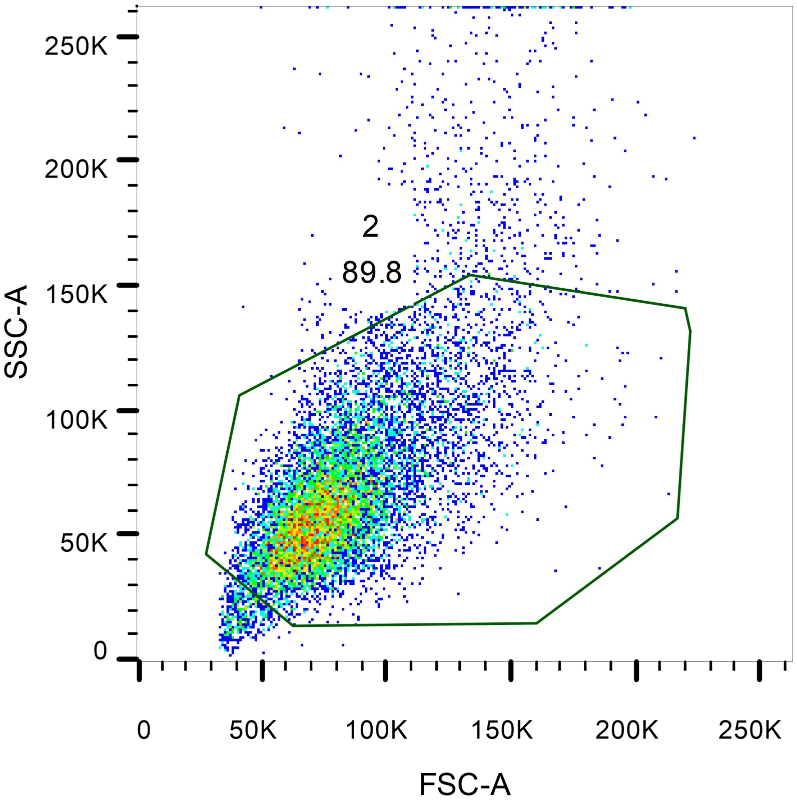

20210922_23_023.fcs
1
11497

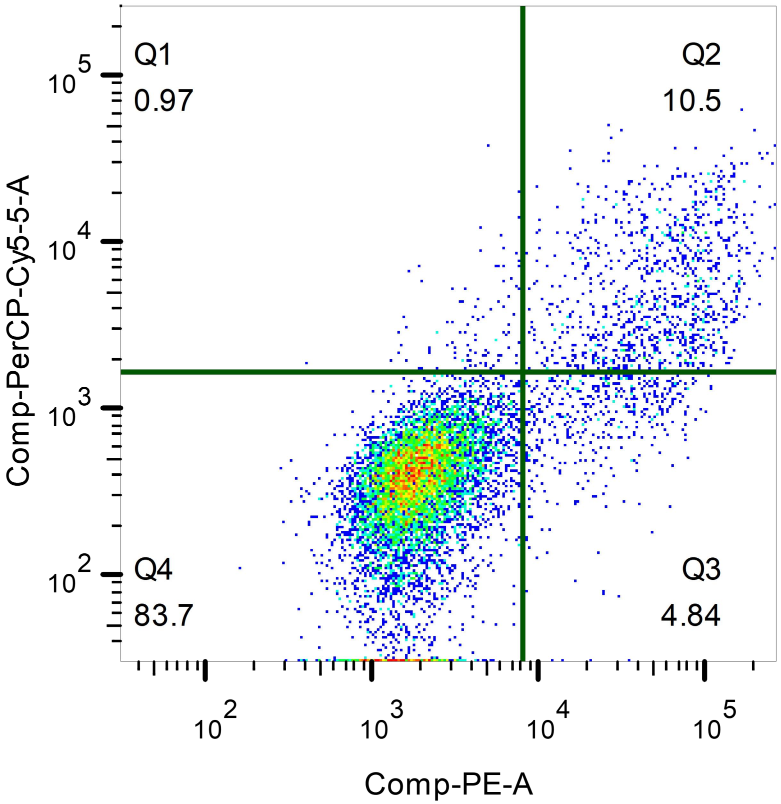

20210922_23_023.fcs
2
10320

4D-MDA-MB-231-TetC+RSL3

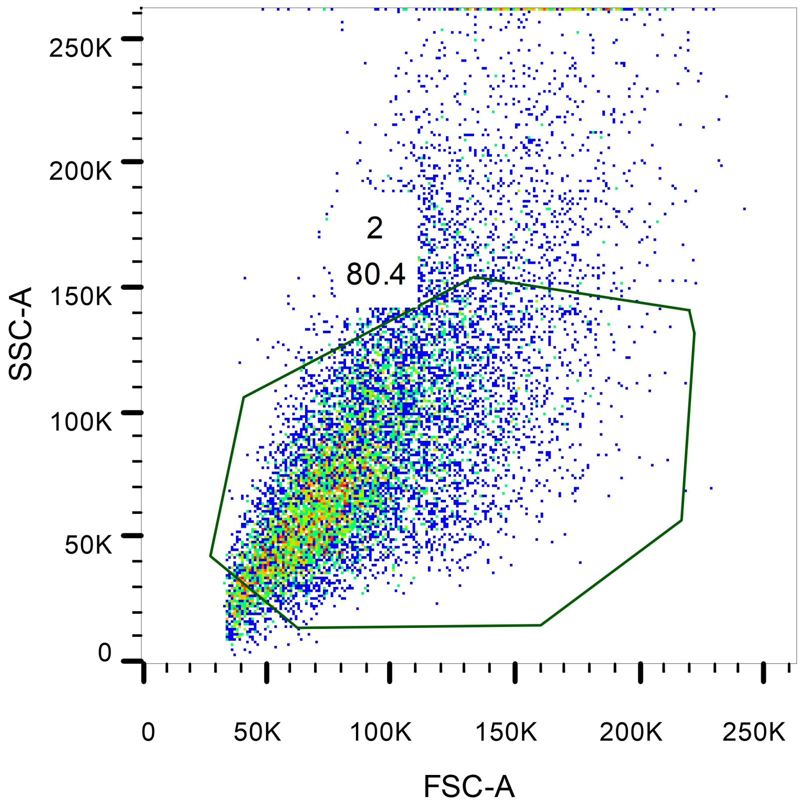

20210922_24_024.fcs
1
12235

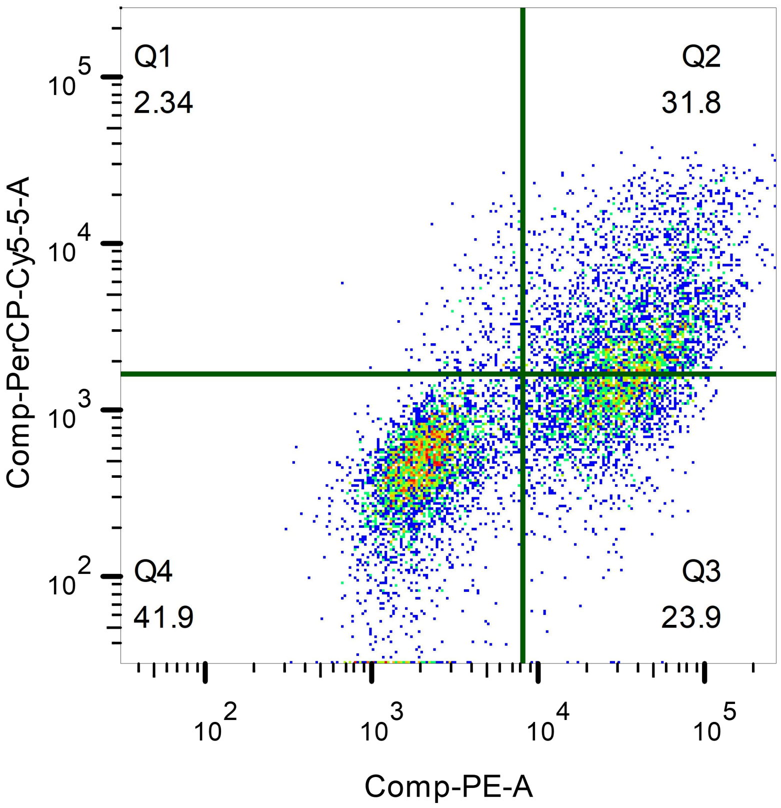

20210922_24_024.fcs
2
9834

5A-MCF7-NCOA4

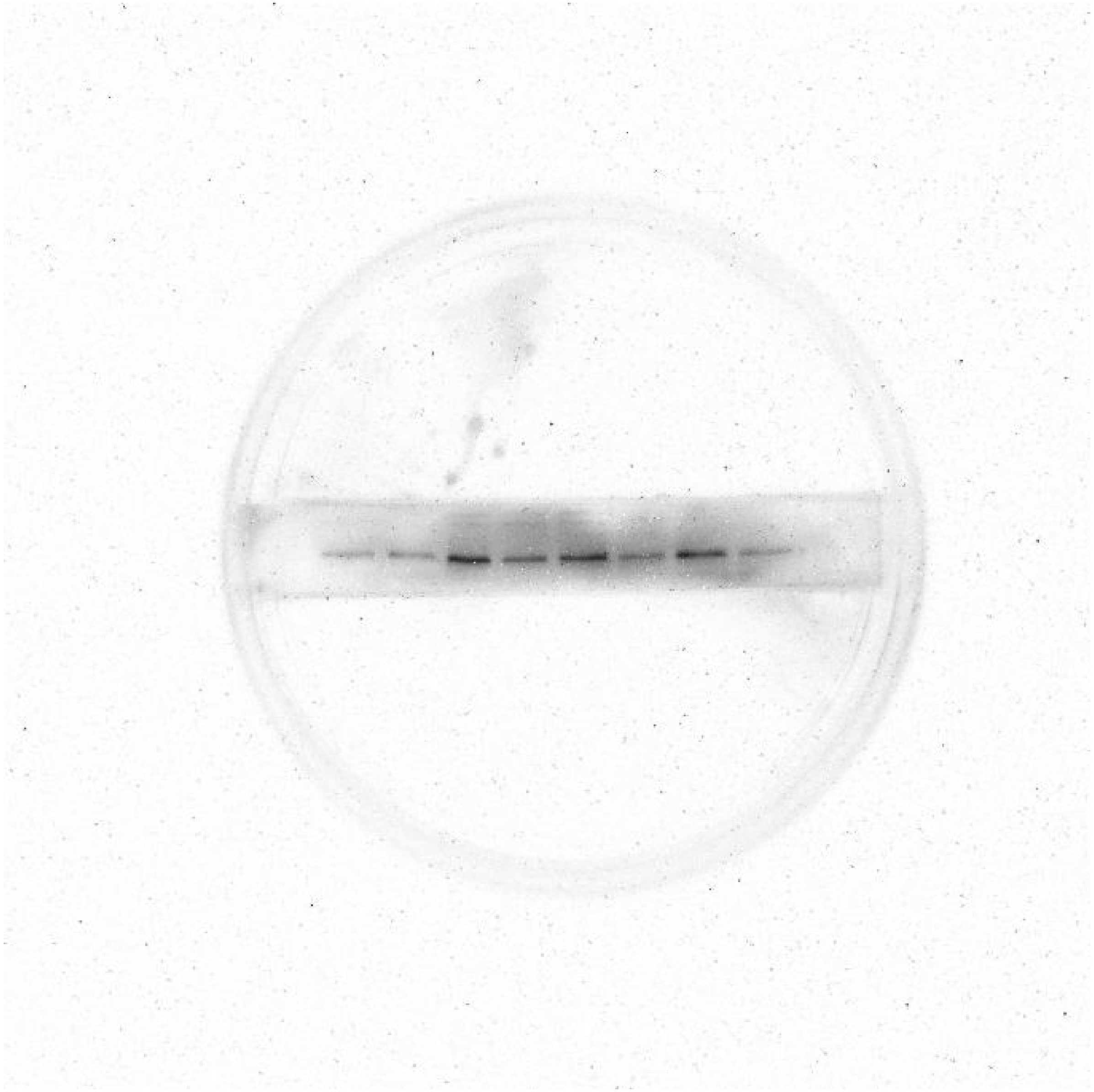

5A-MCF7-FTH1

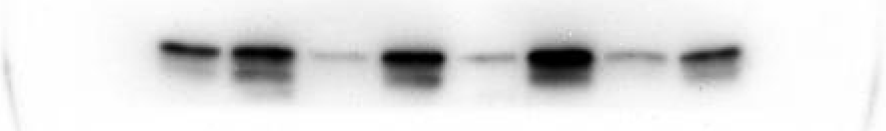

5A-MCF7-GPX4

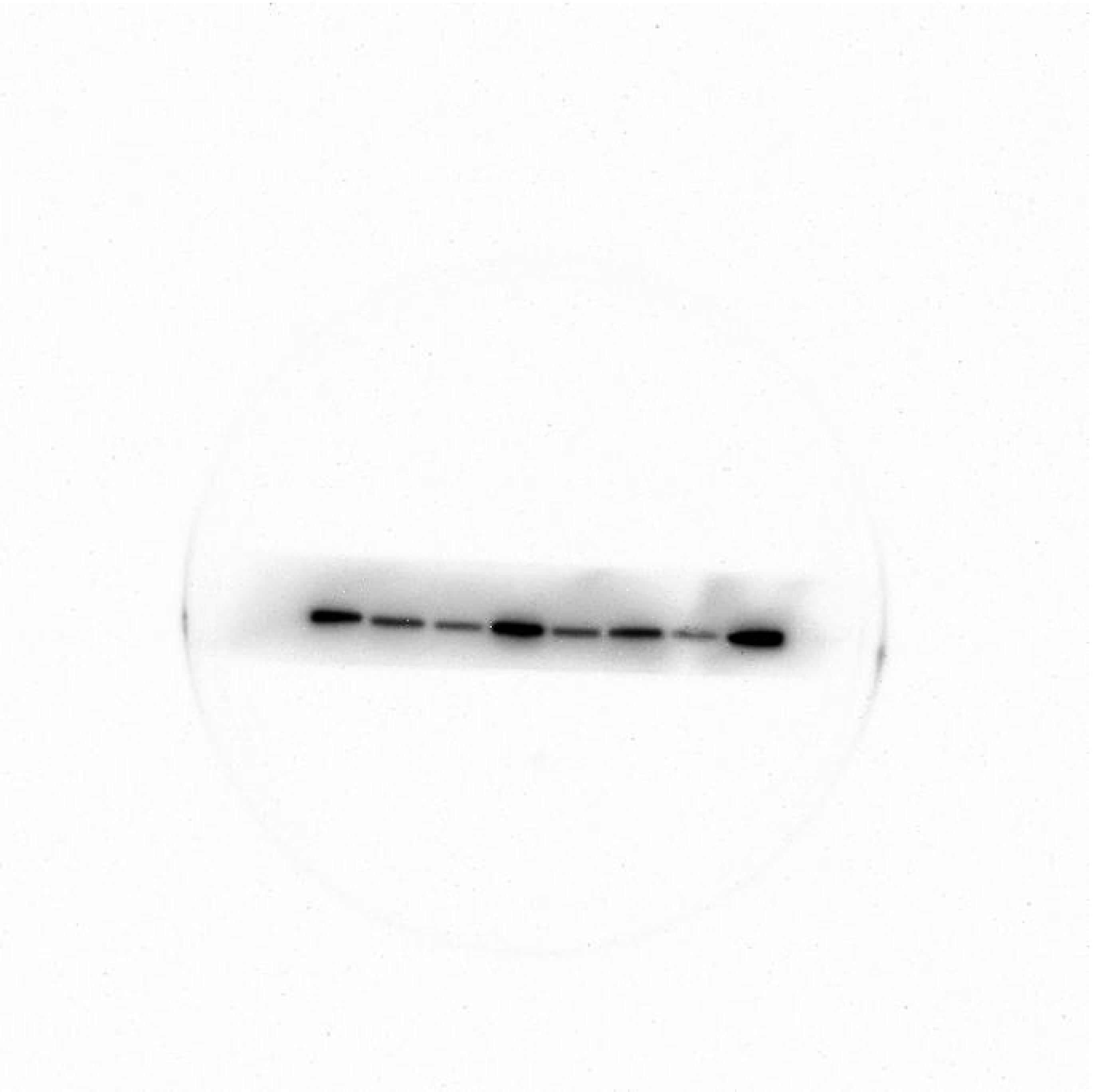

5A-MCF7-β-actin

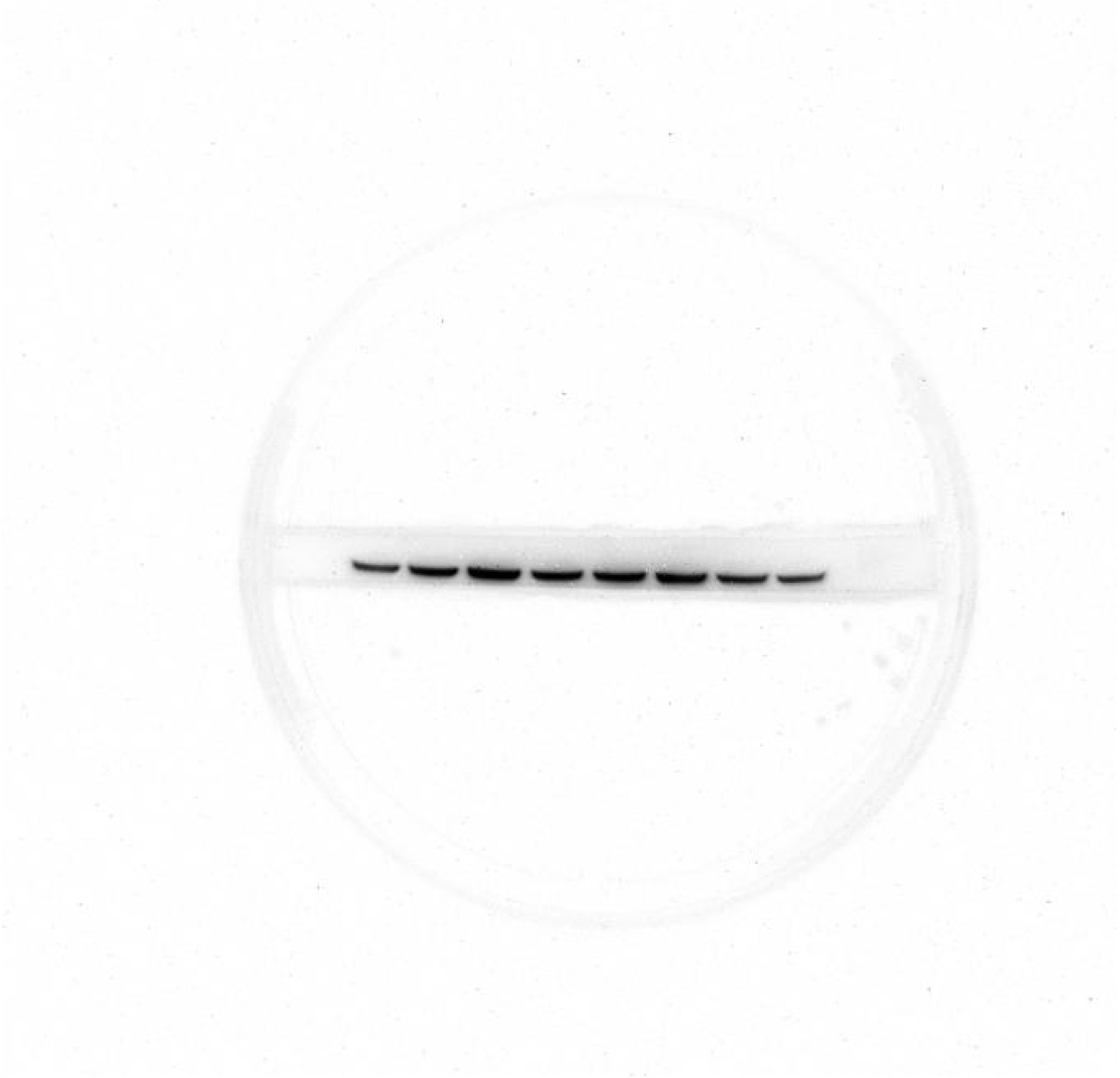

5B-MDA-MB-231-NCOA4

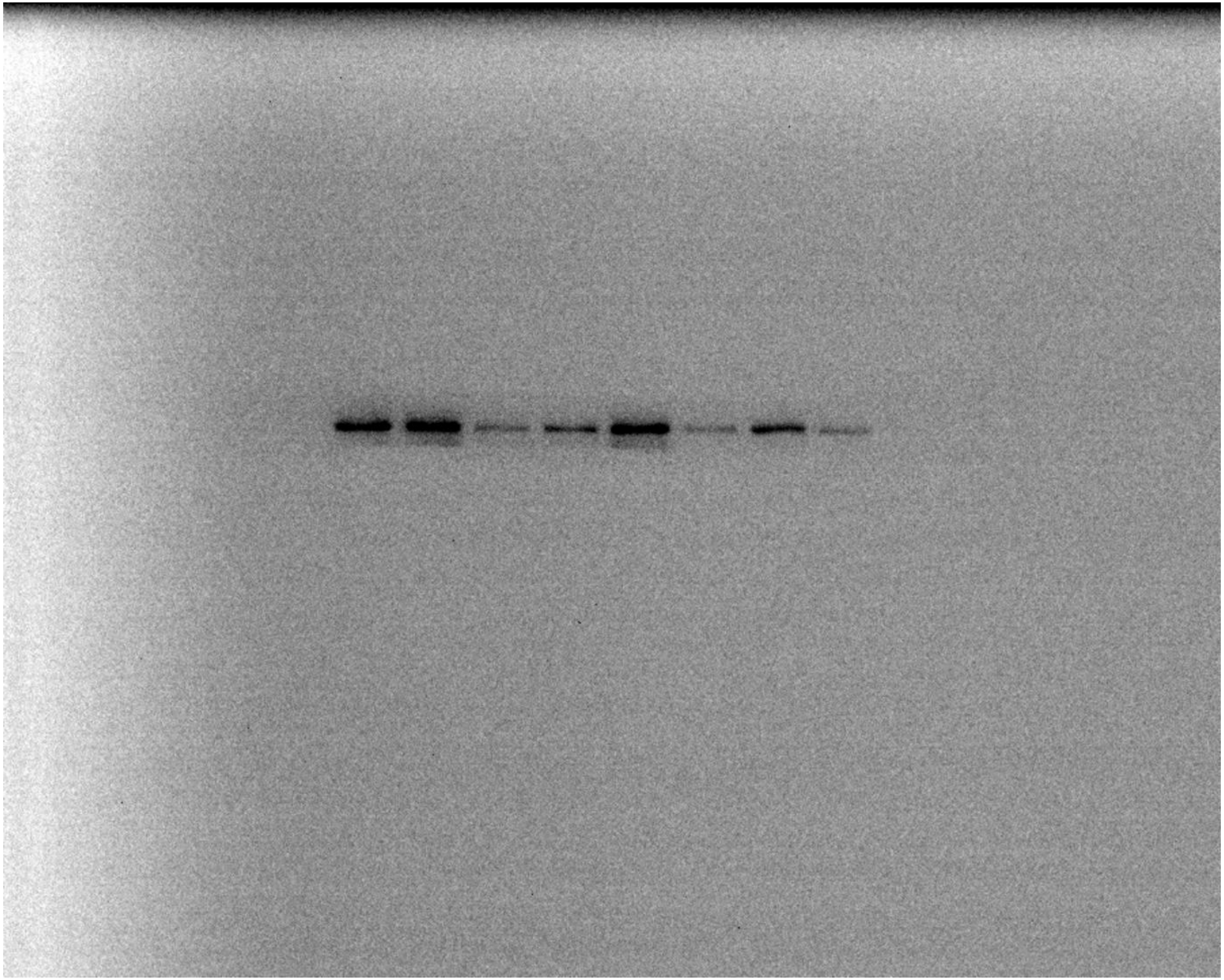

5B-MDA-MB-231-FTH1

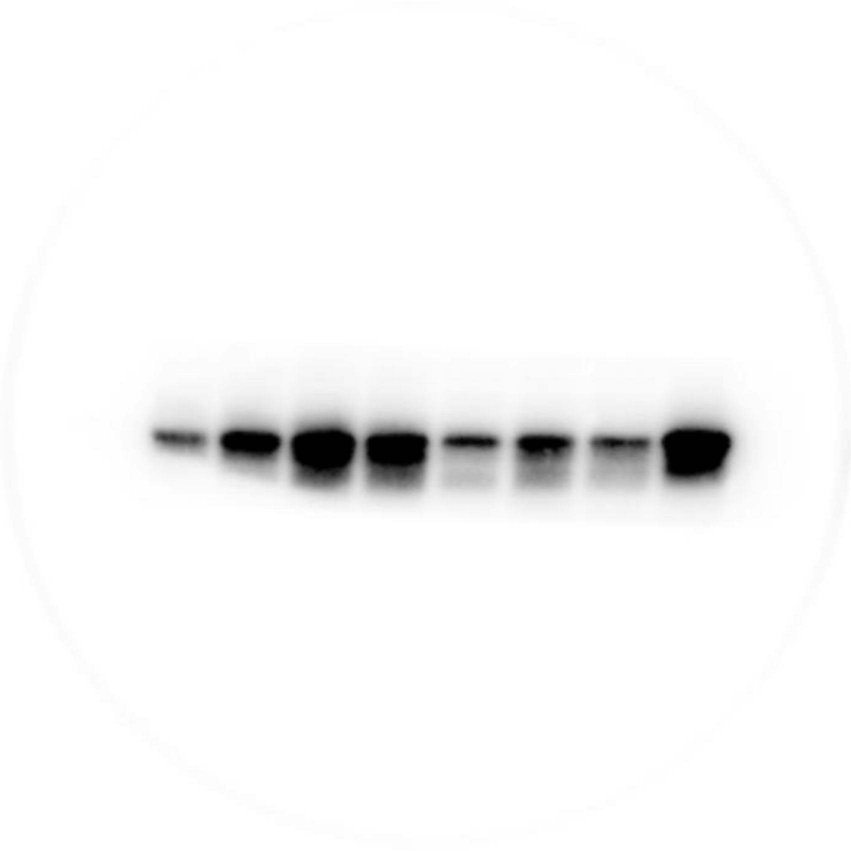

5B-MDA-MB-231-GPX4

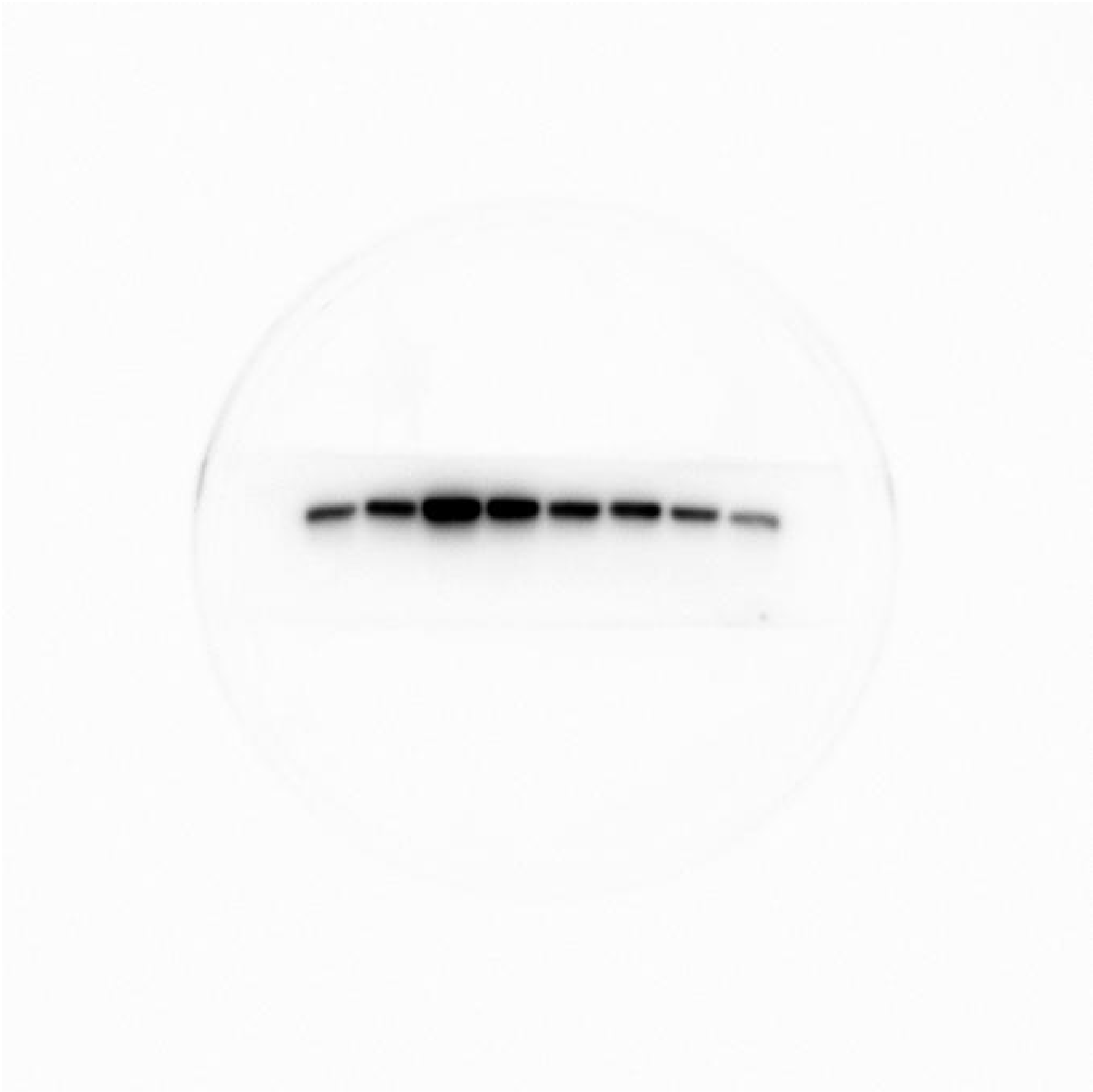

5B-MDA-MB-231-β-actin

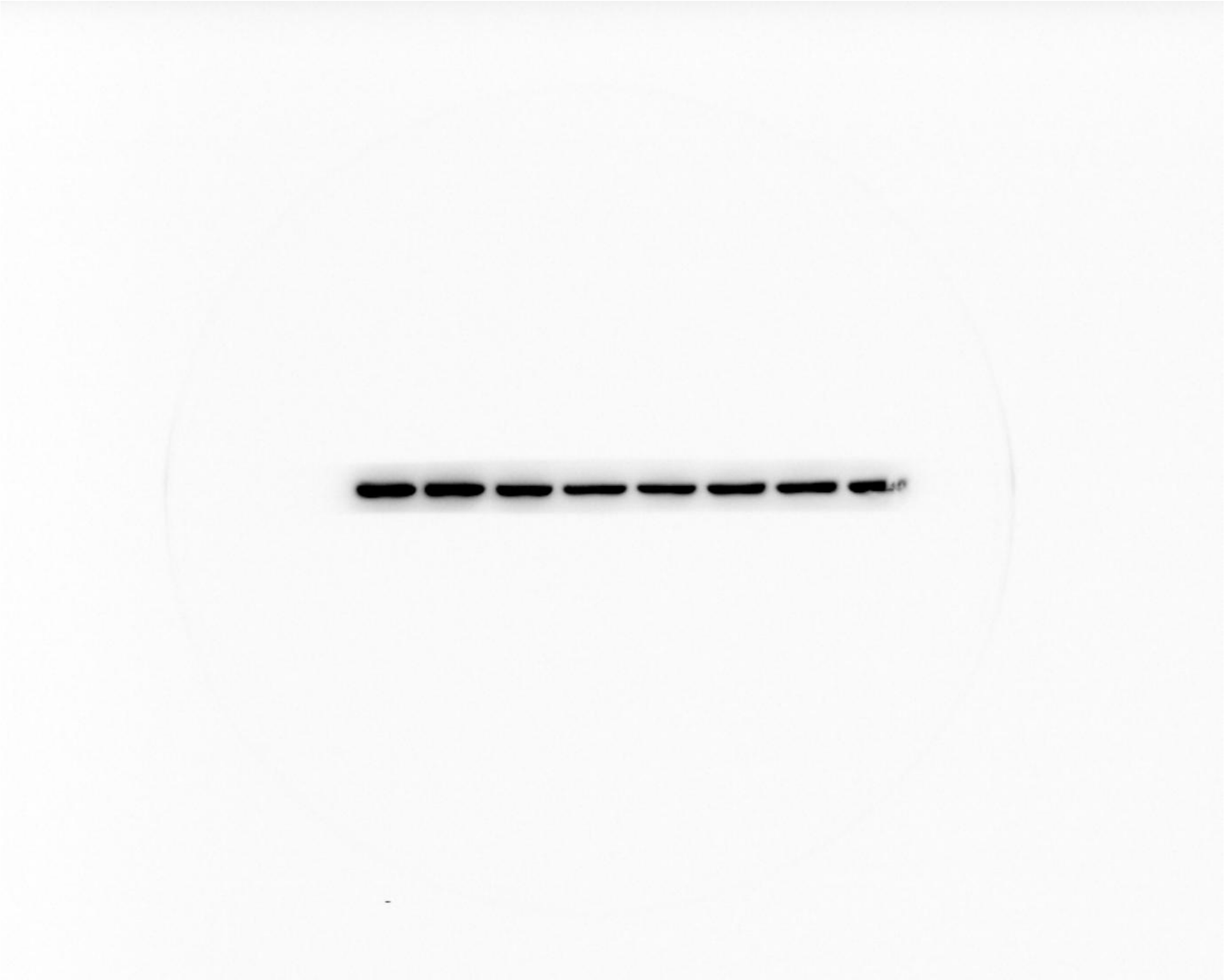

5C-MCF7-NCOA4

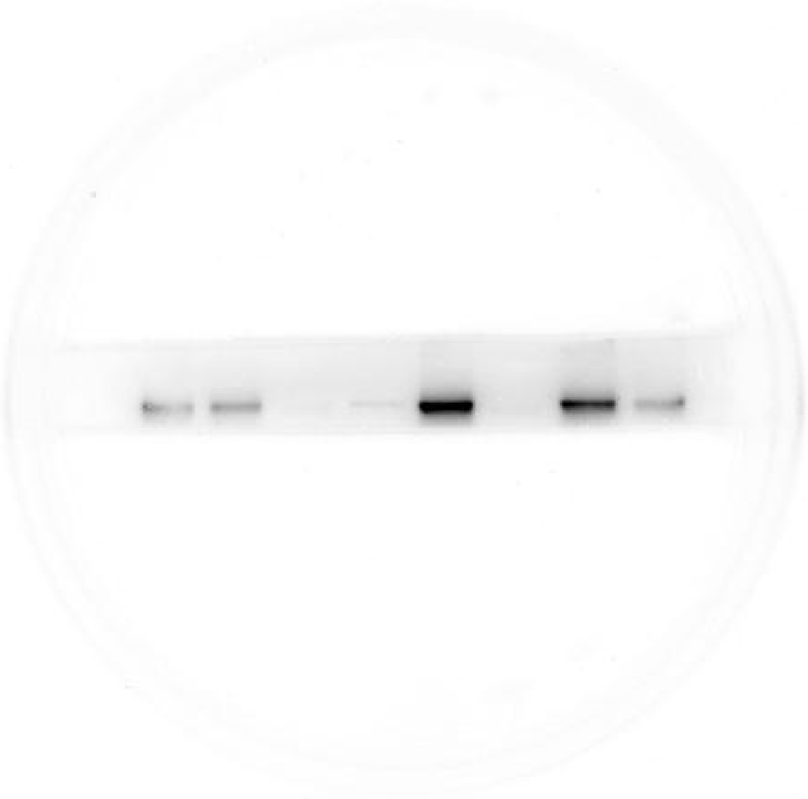

5C-MCF7-FTH1

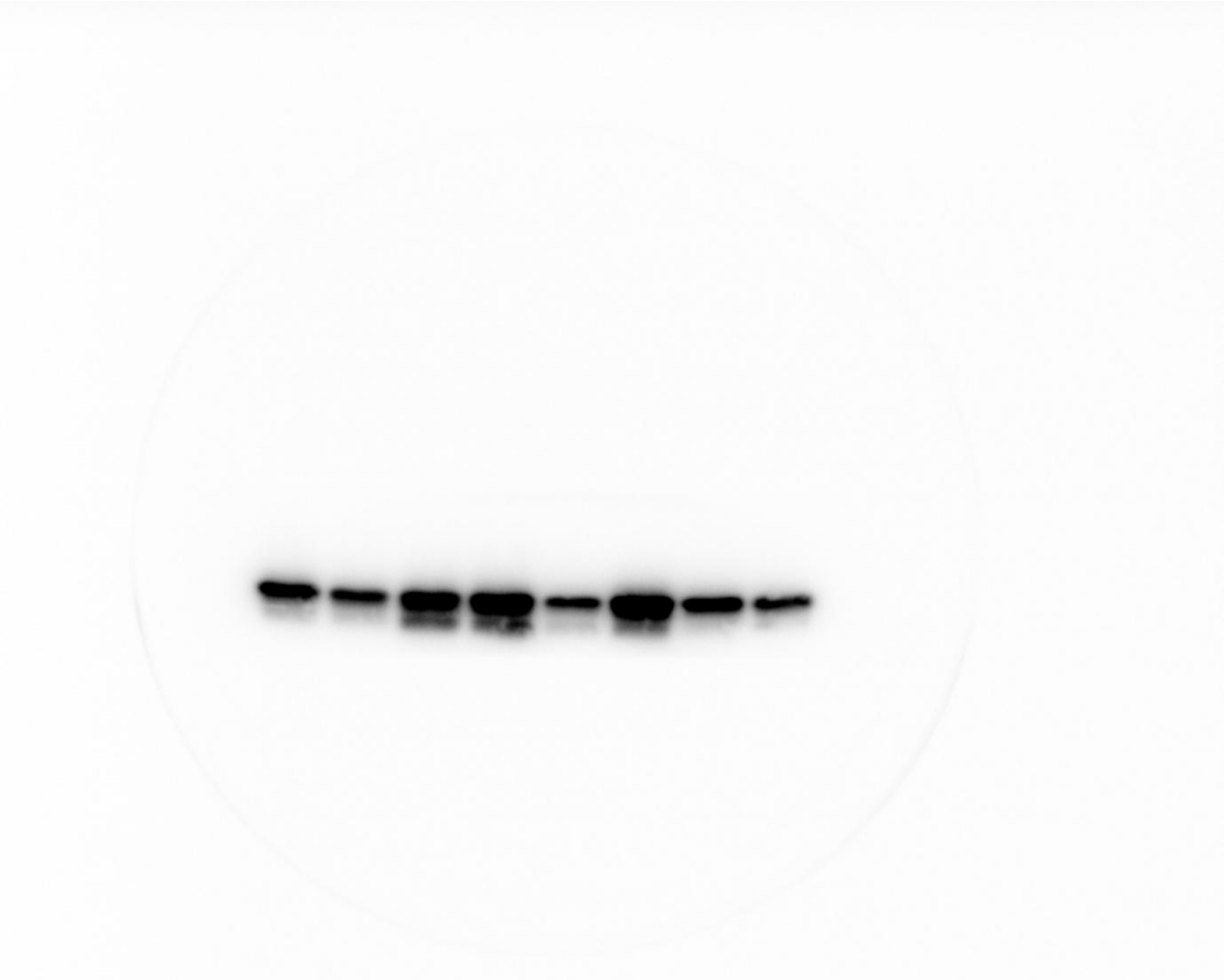

5C-MCF7-GPX4

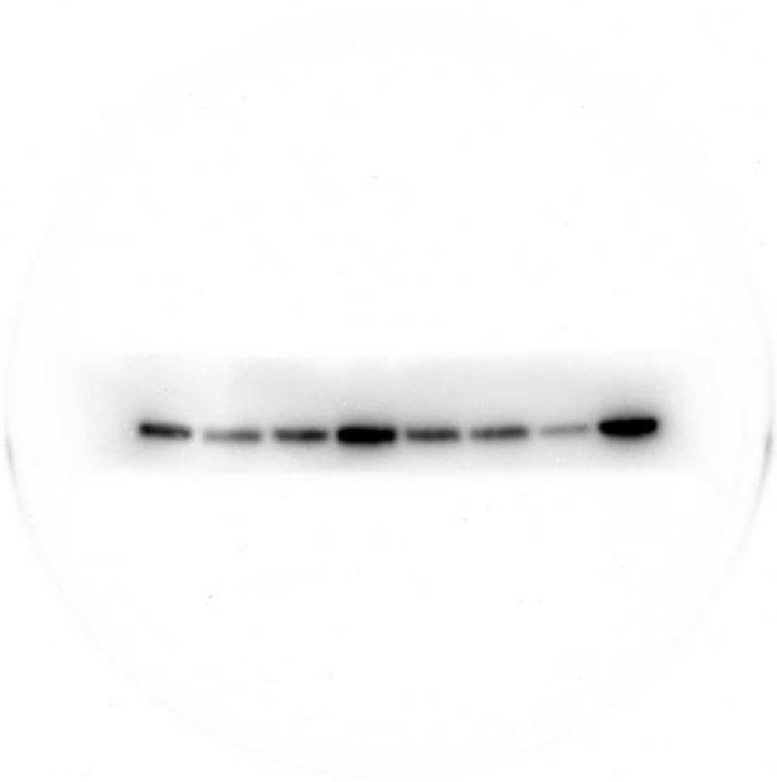

5C-MCF7-β-actin

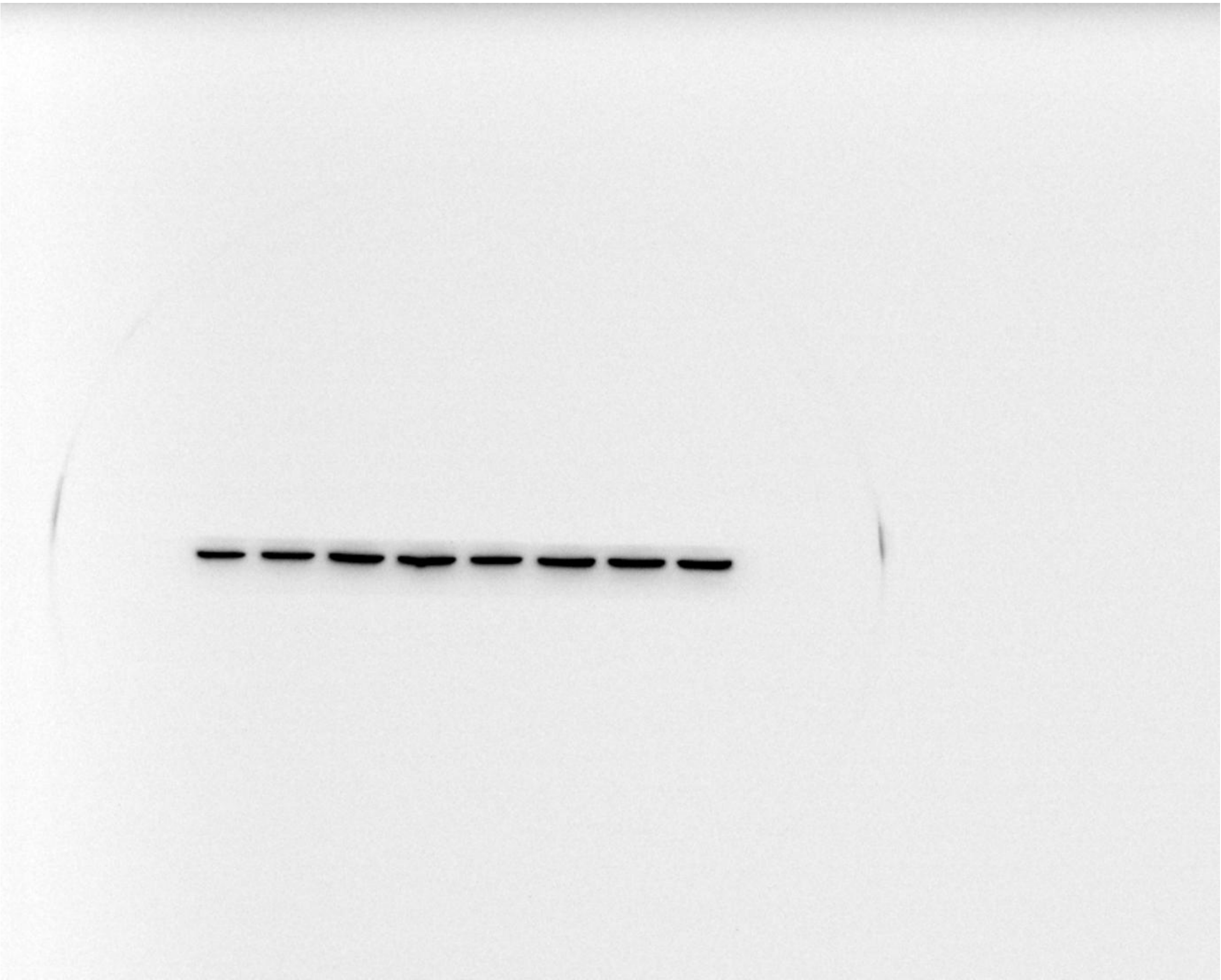

5D-MDA-MB-231-NCOA4

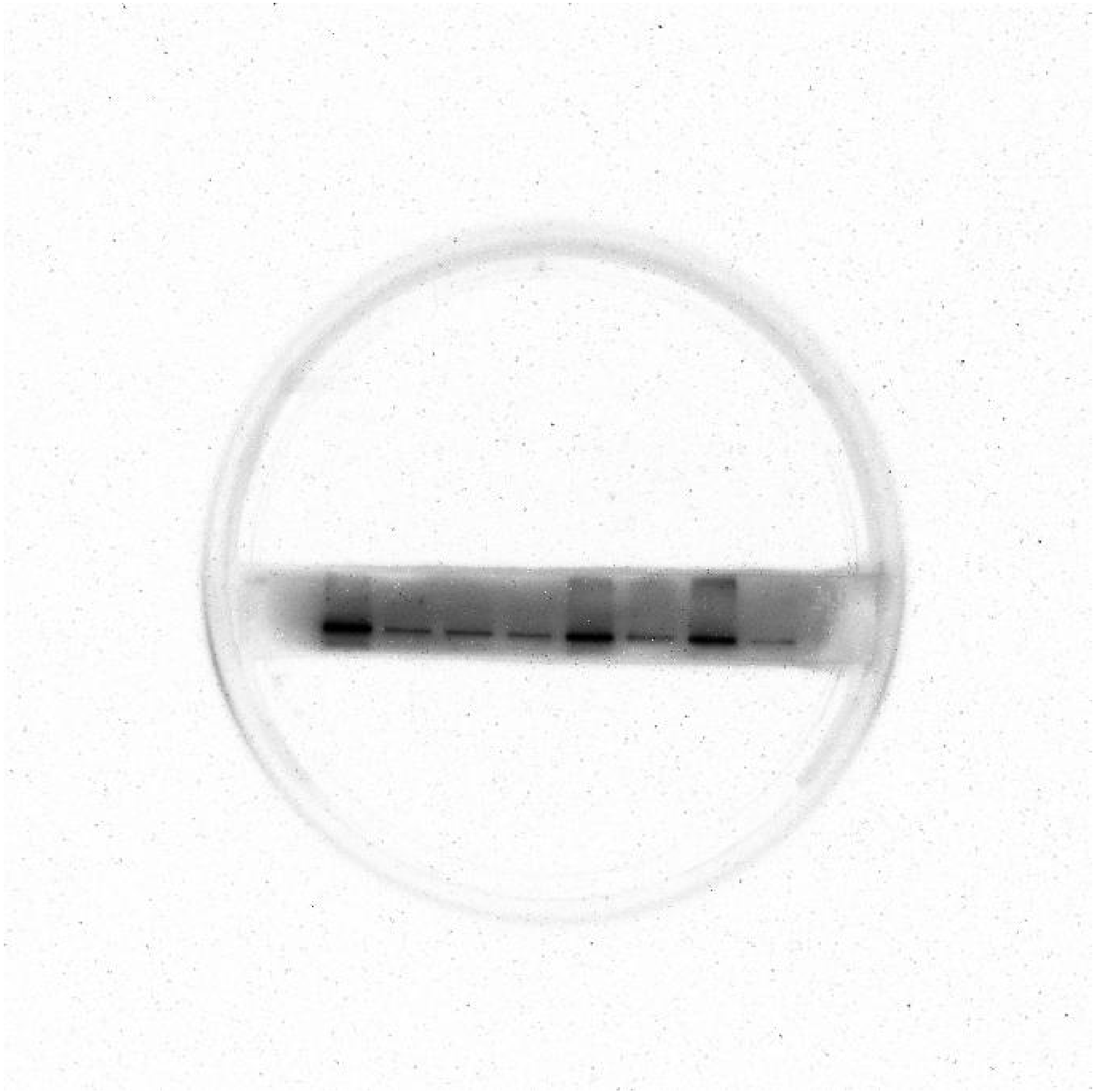

5D-MDA-MB-231-FTH1

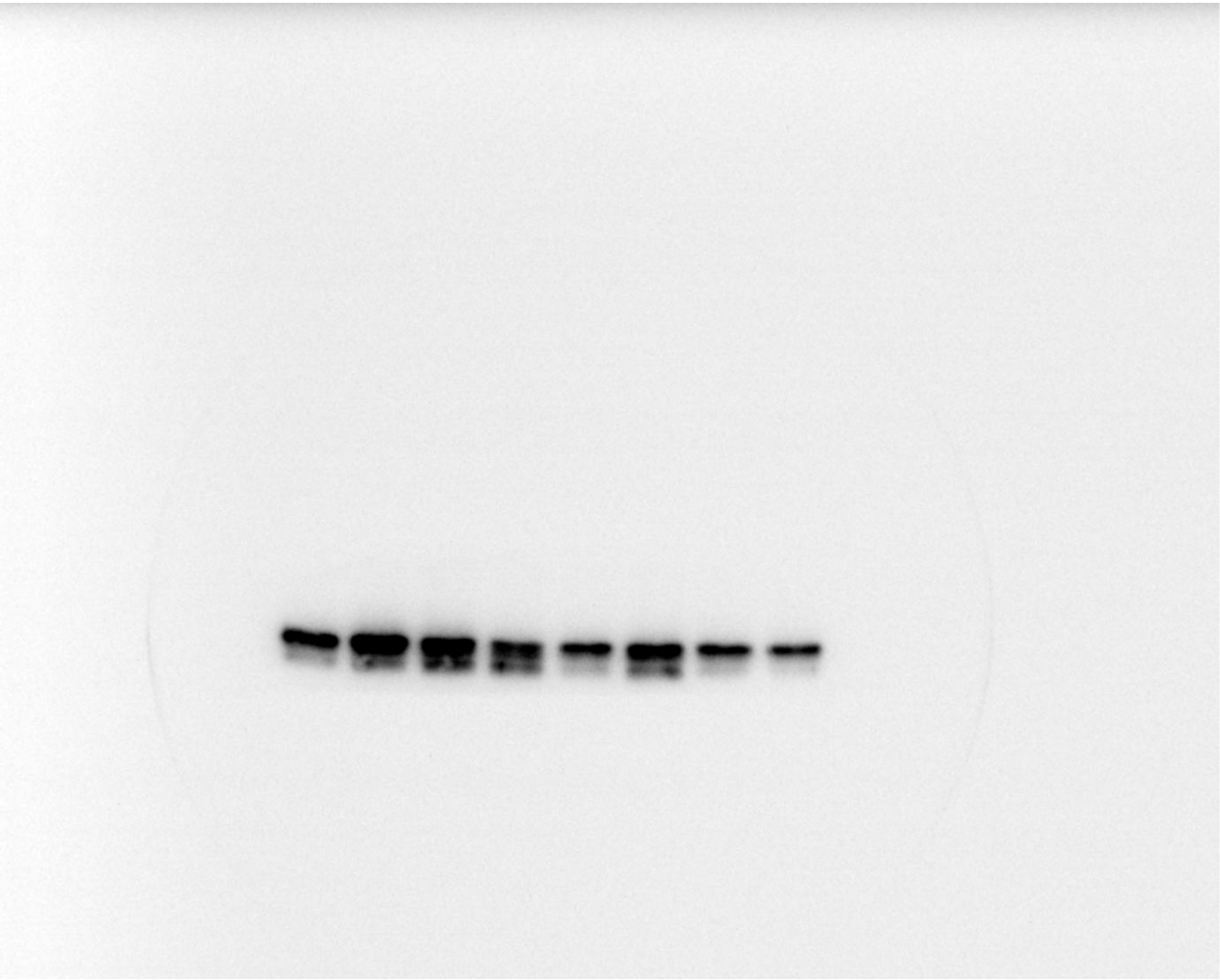

5D-MDA-MB-231-GPX4

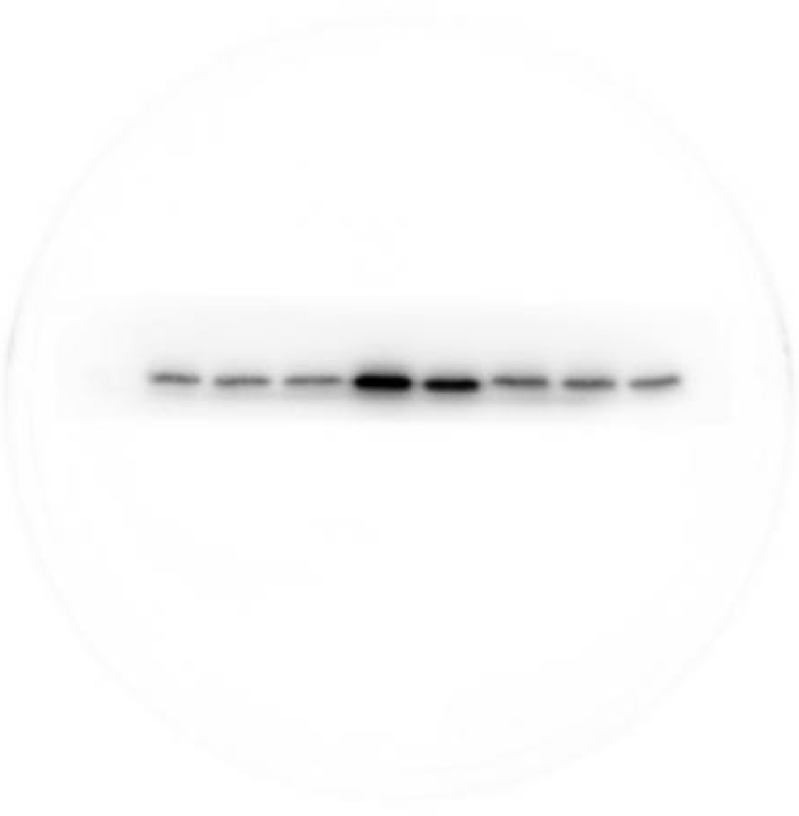

5D-MDA-MB-231-β-actin

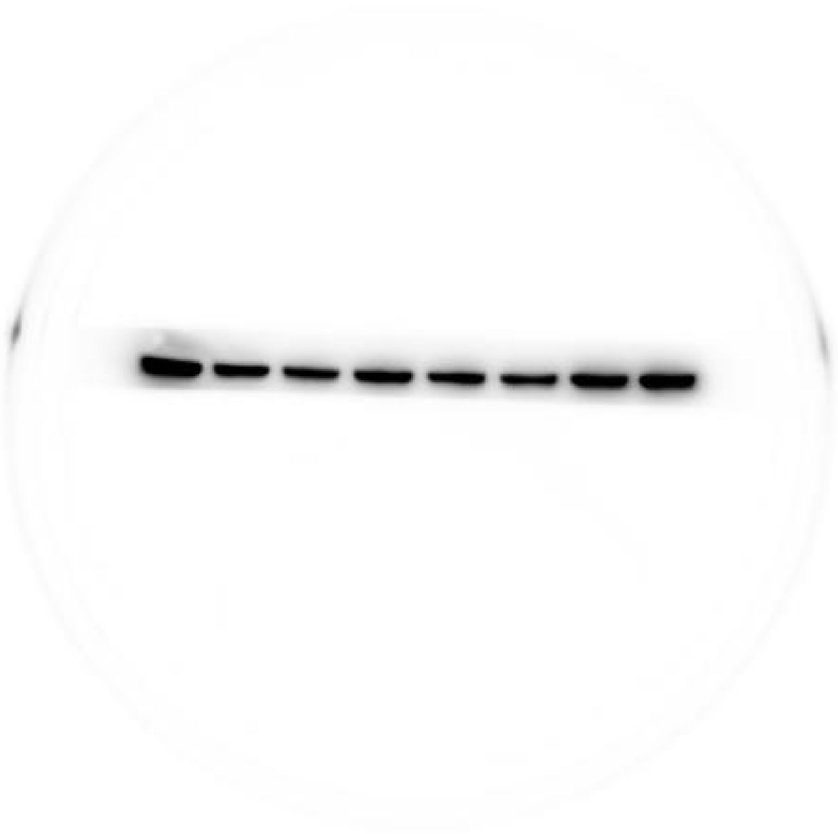

6A-MCF7-DMSO

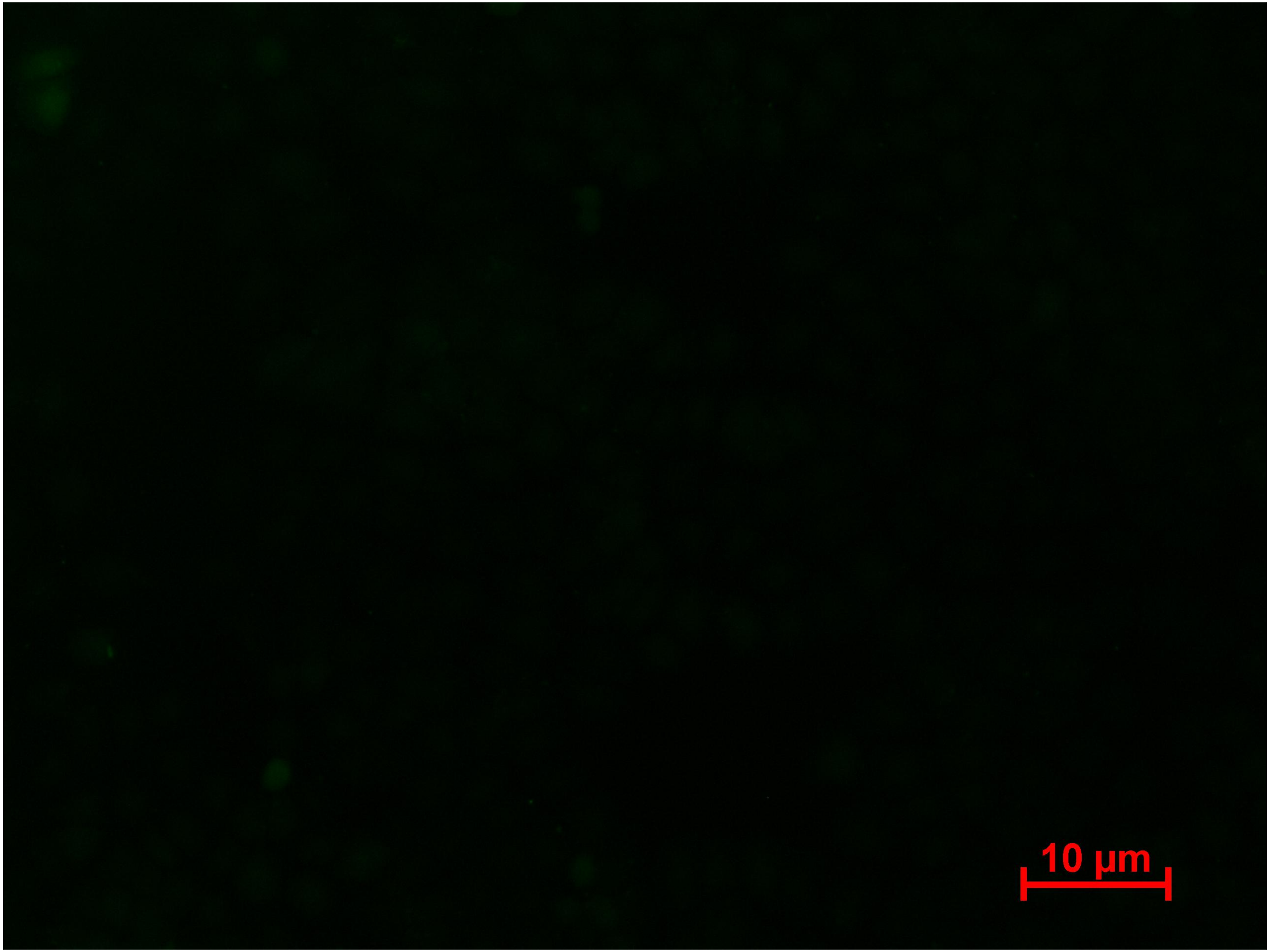

6A-MCF7-TetC

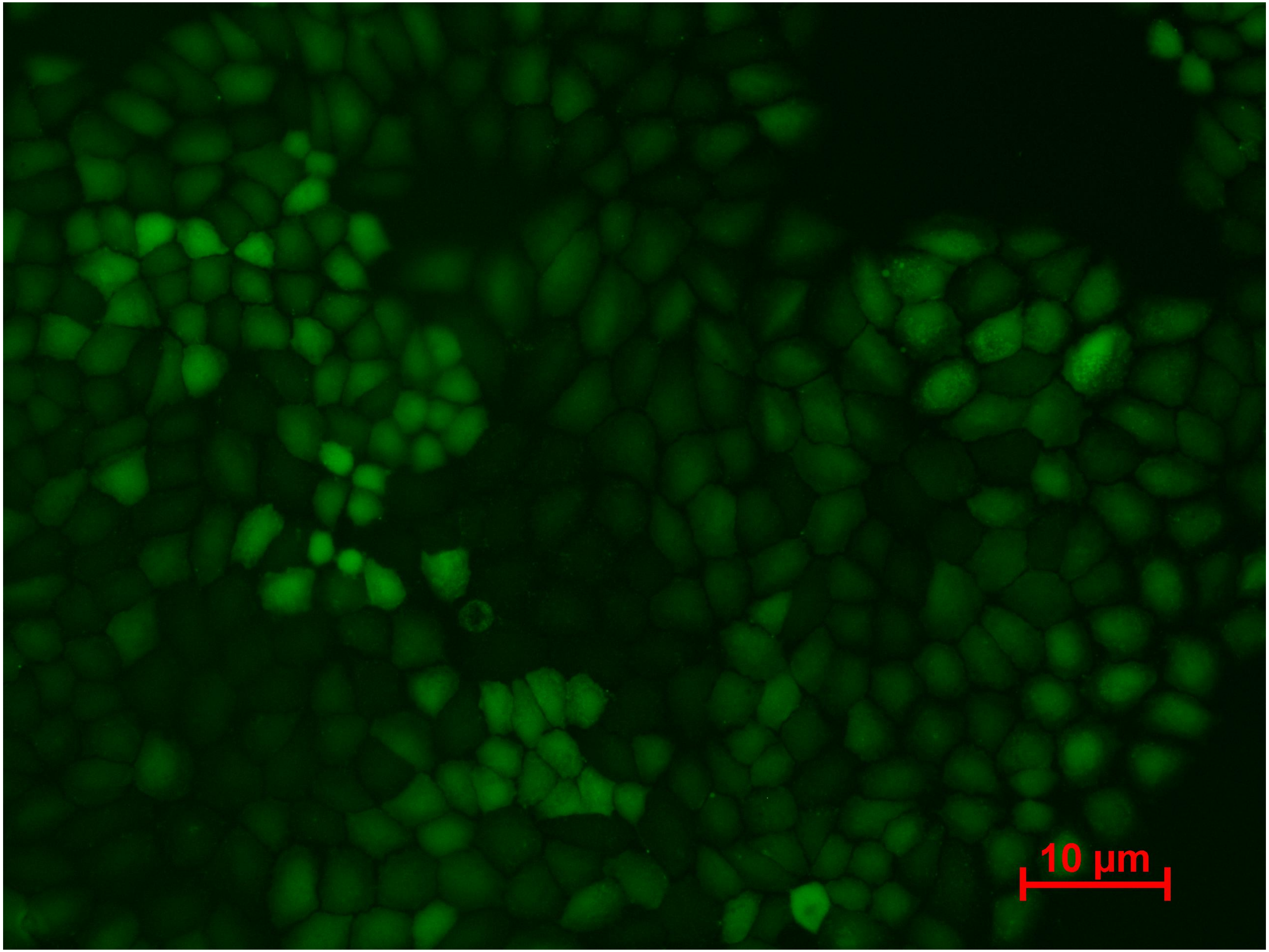

6A-MCF7-TetC+Fer-1

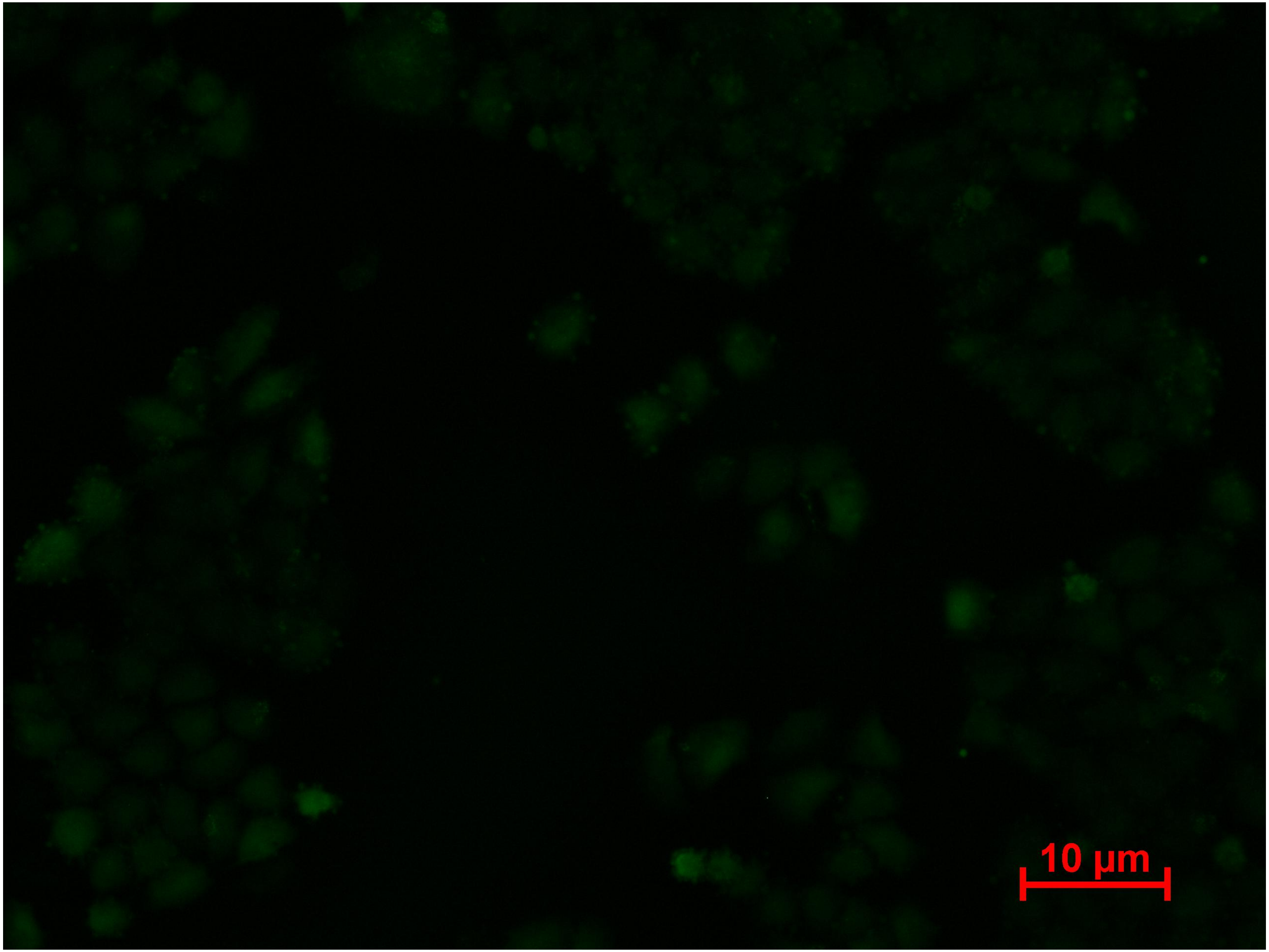

6A-MDA-MB-231-DMSO

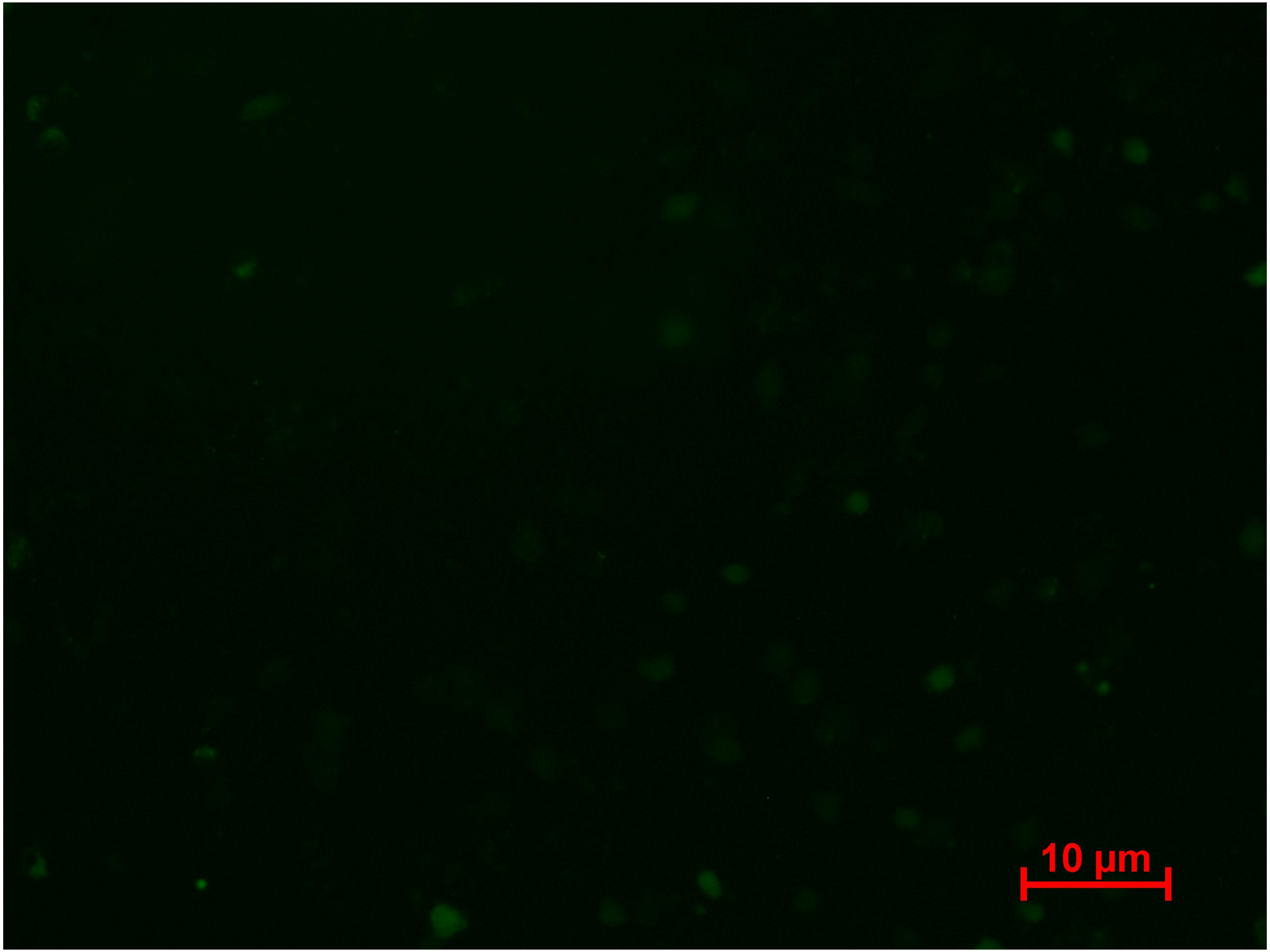

6A-MDA-MB-231-TetC

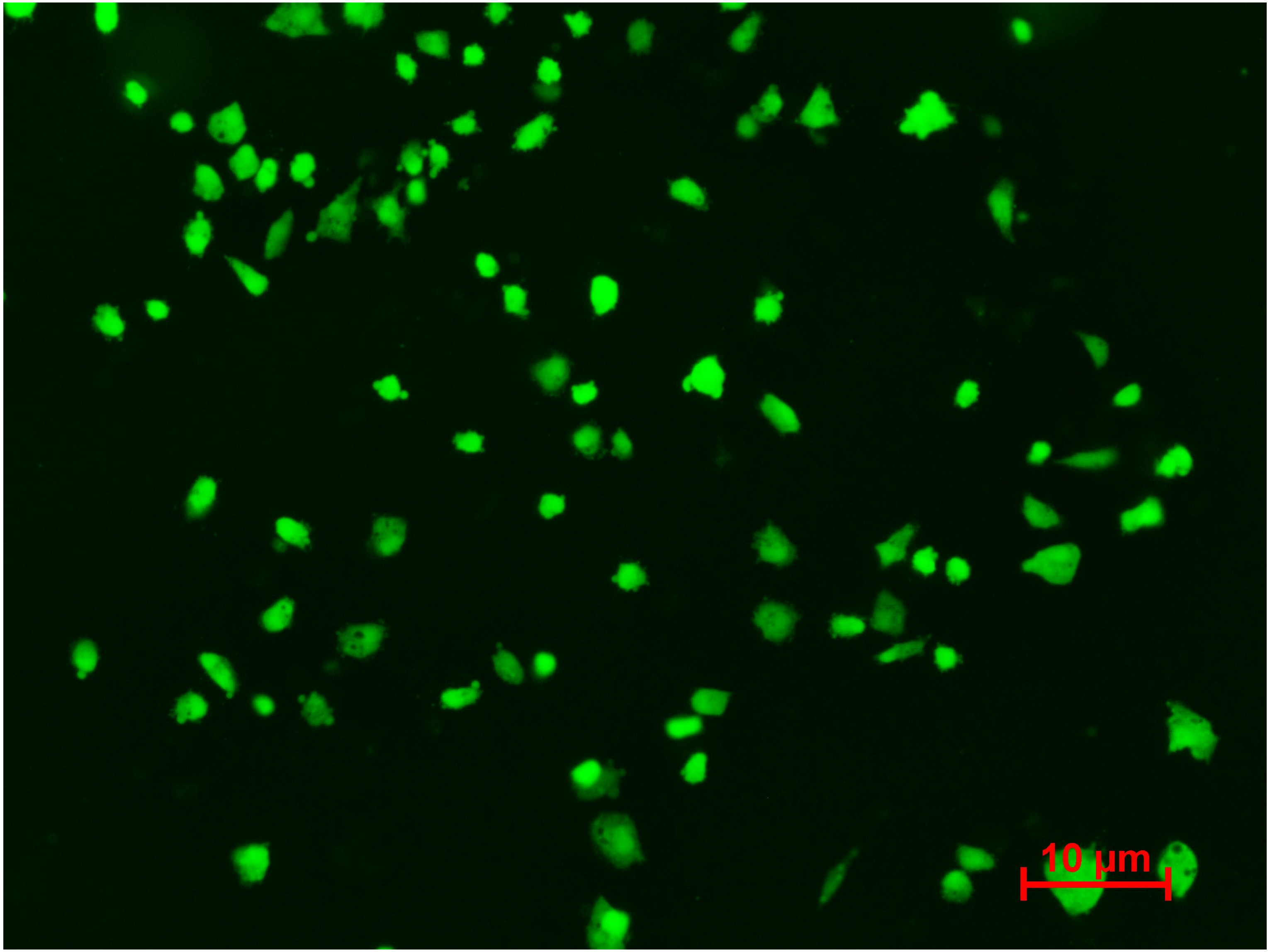

6A-MDA-MB-231-TetC+Fer-1

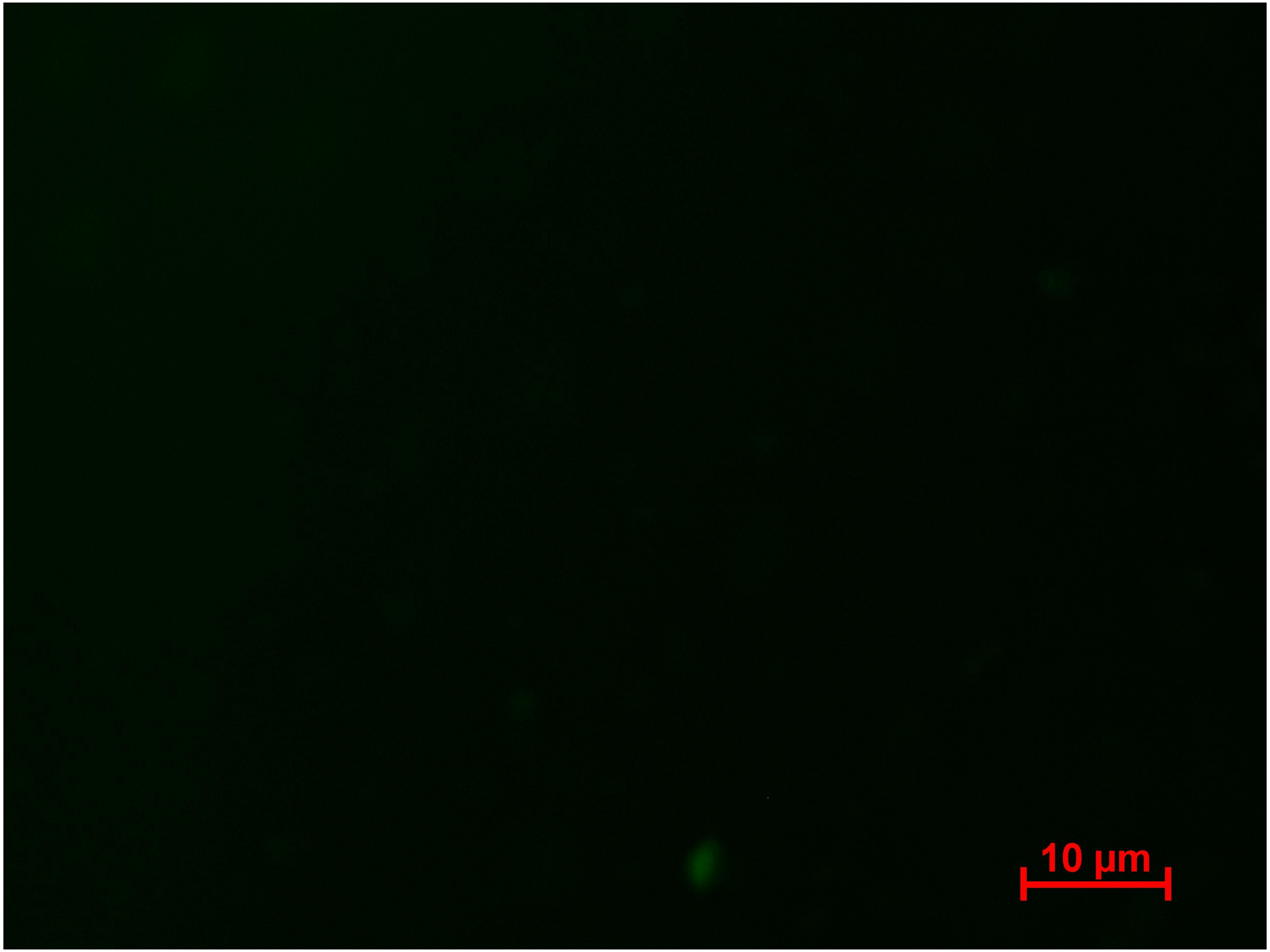

6D-MCF7-NCOA4

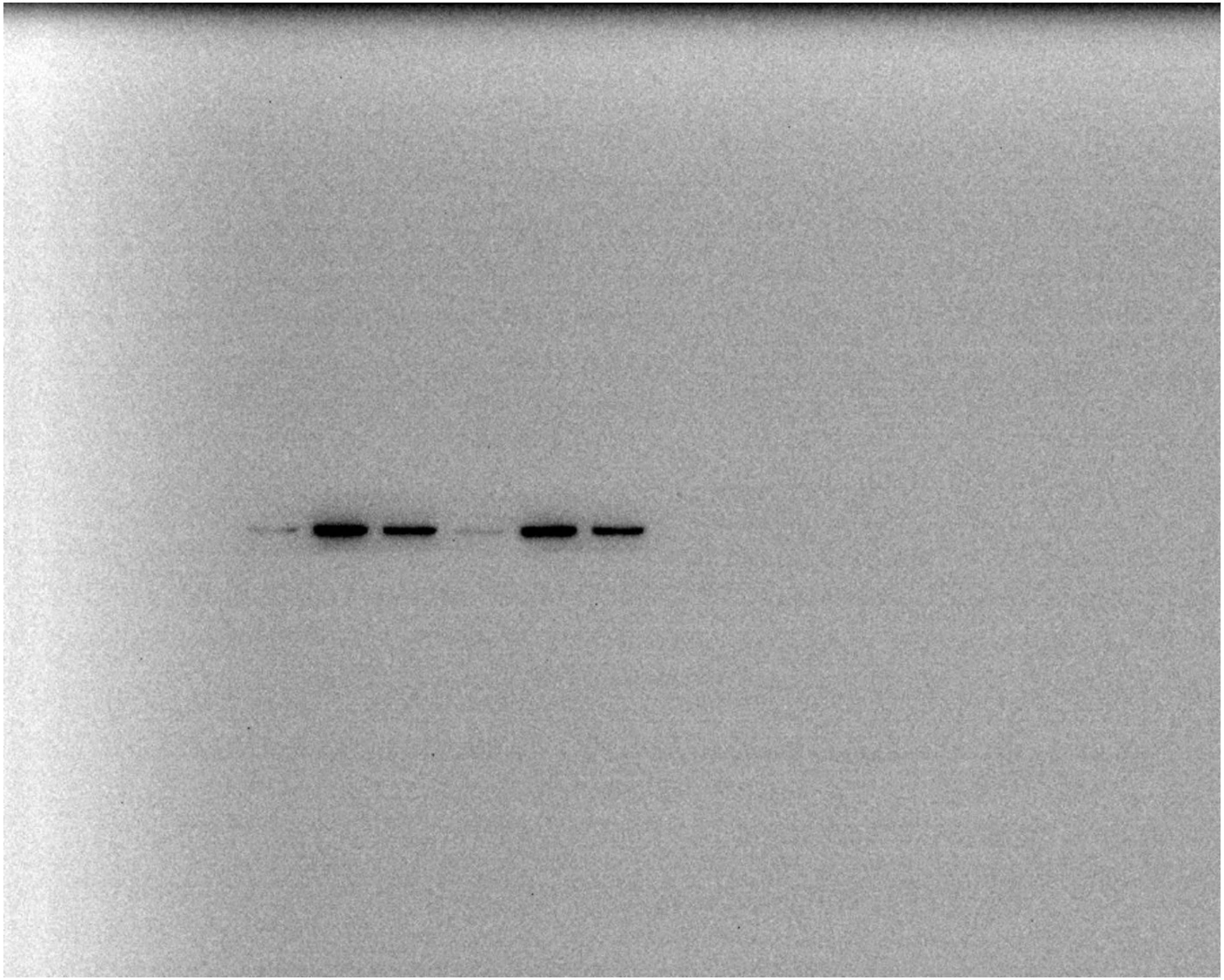

6D-MCF7-FTH1

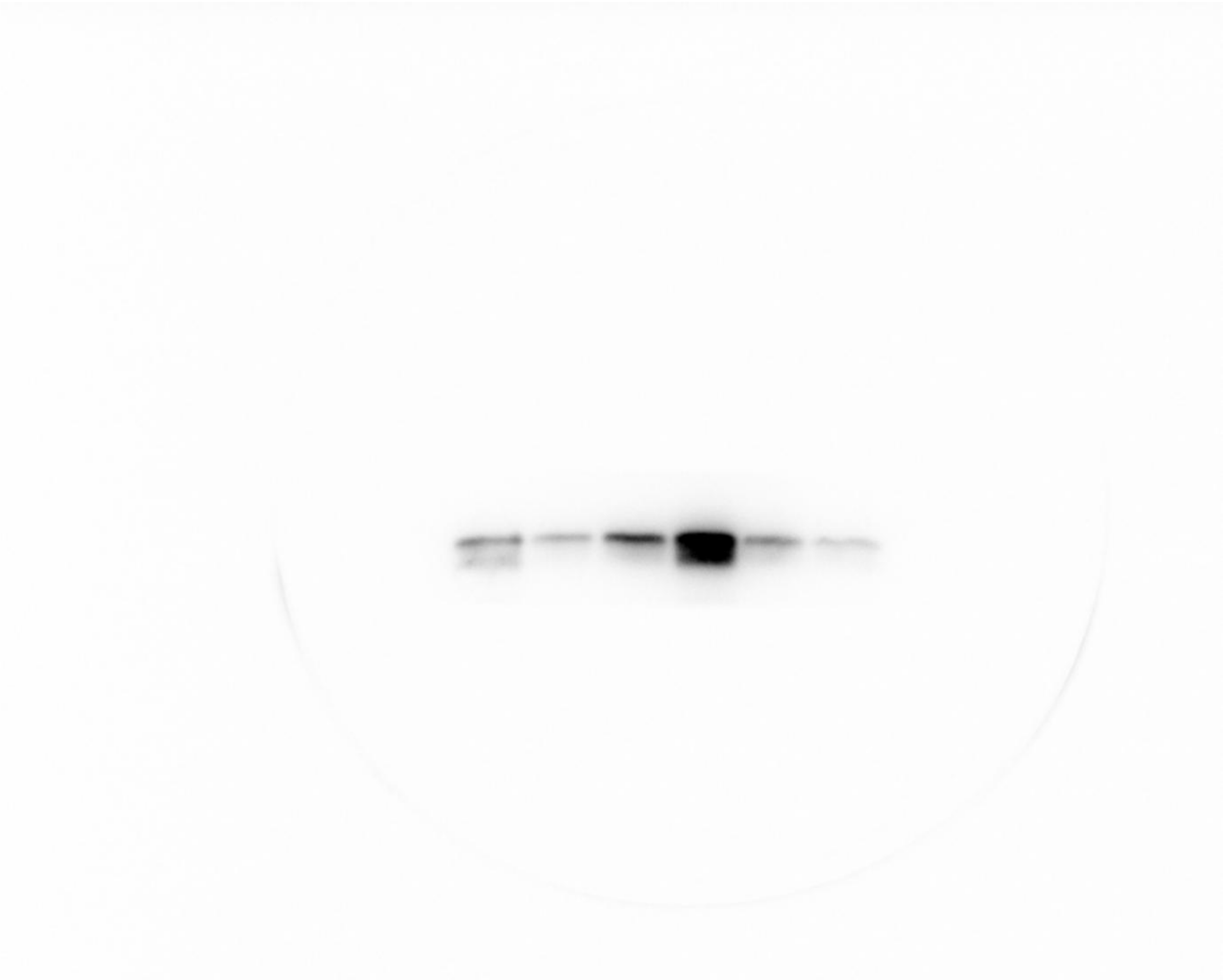

6D-MCF7-GPX4

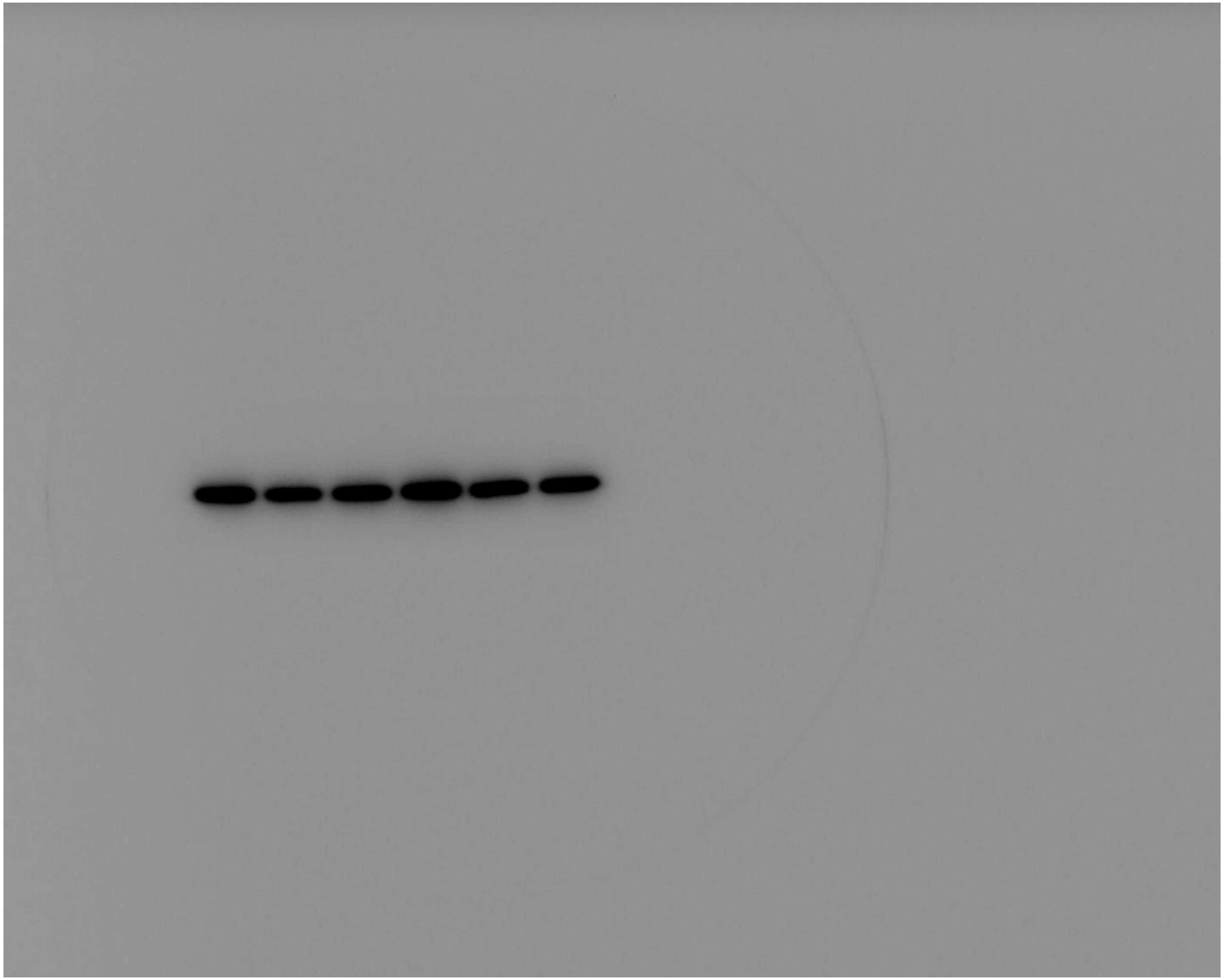

6D-MCF7-β-actin

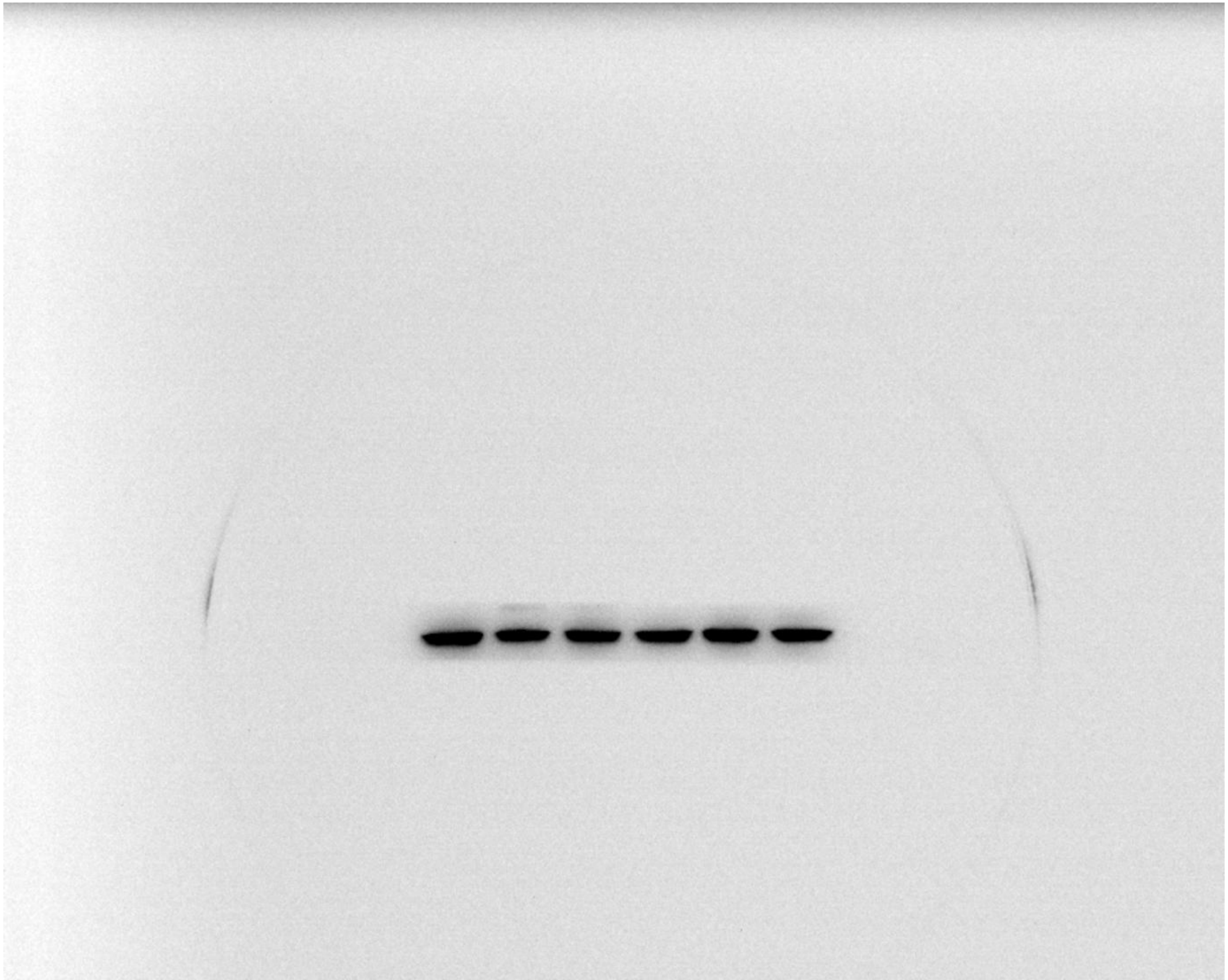

6E-MDA-MB-231-NCOA4

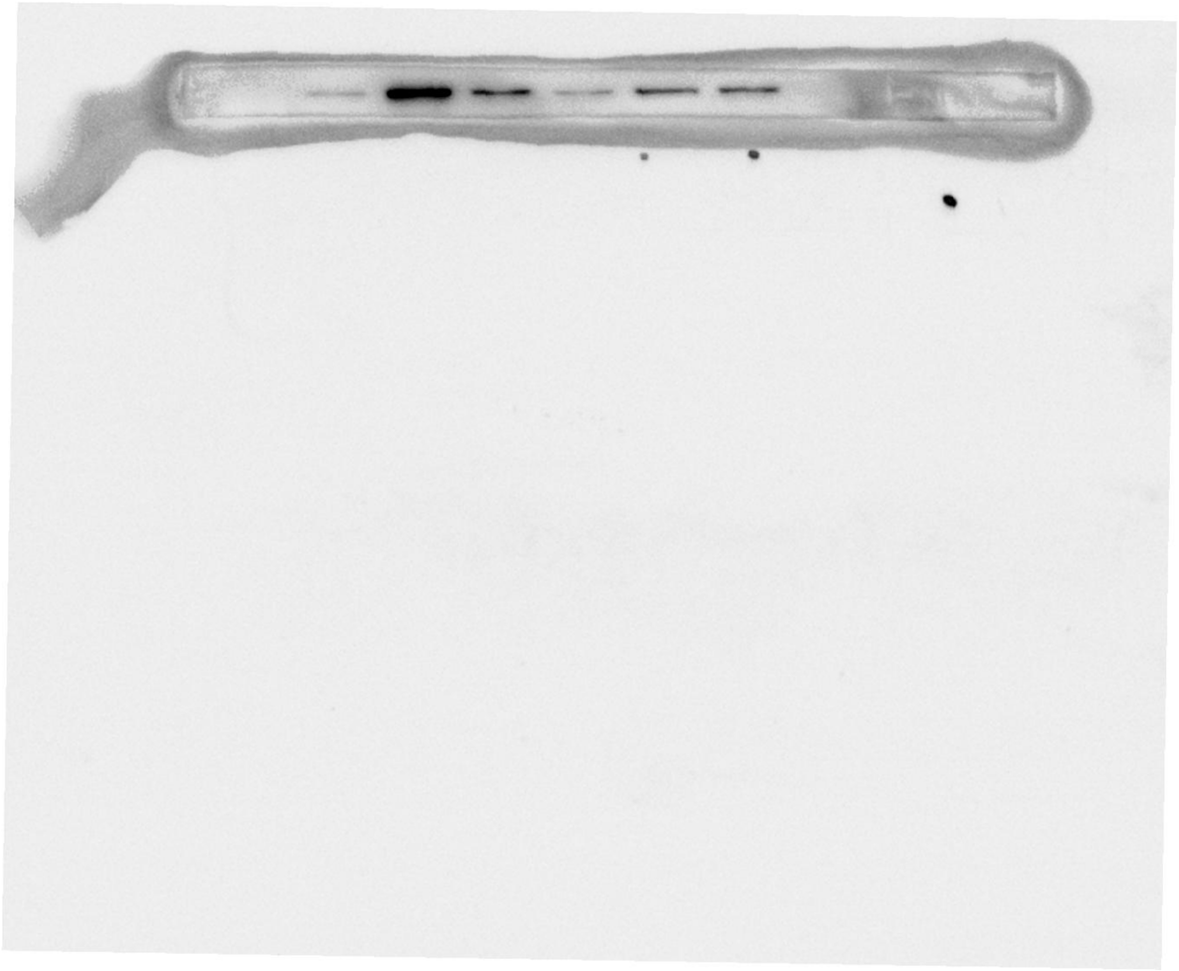

6E-MDA-MB-231-FTH1

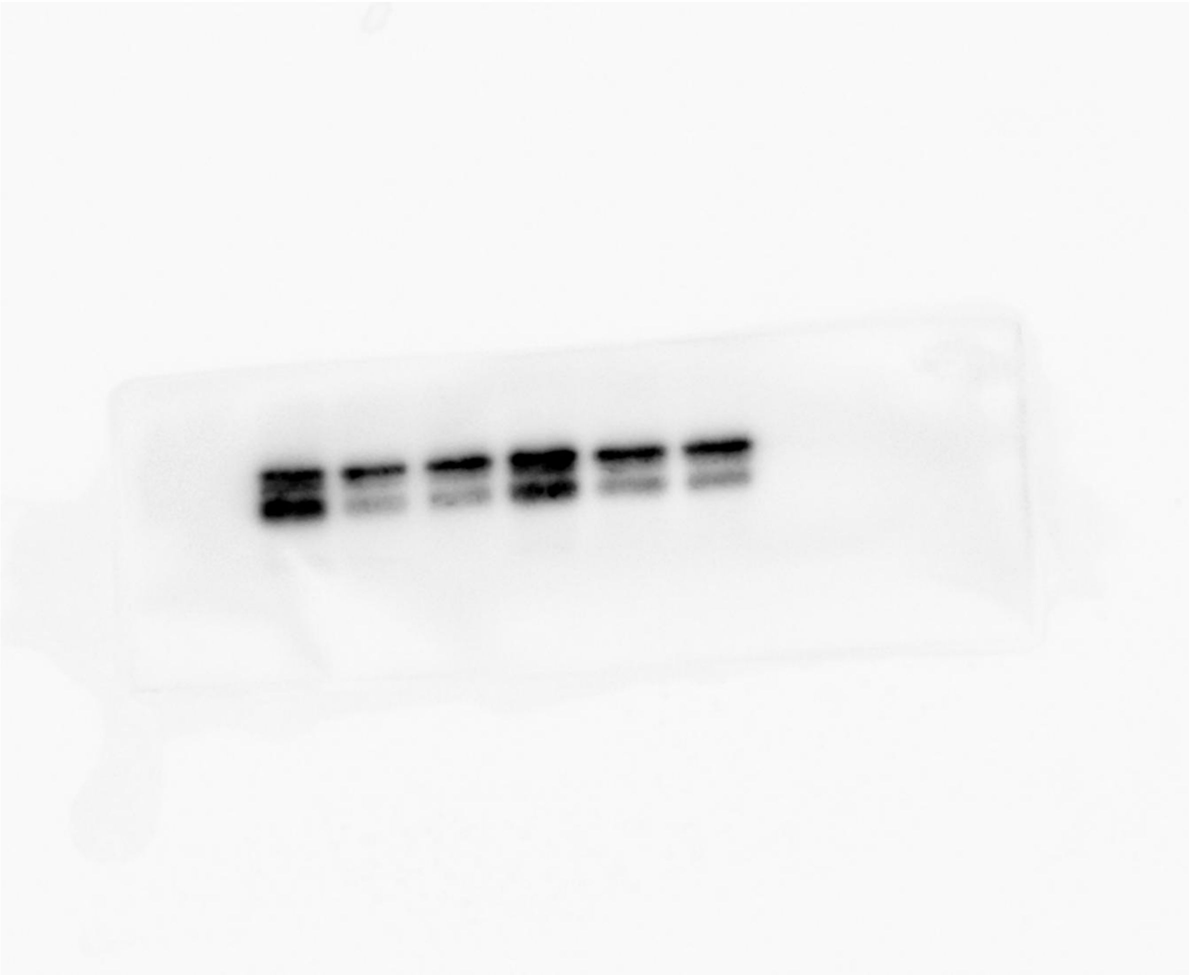

6E-MDA-MB-231-GPX4

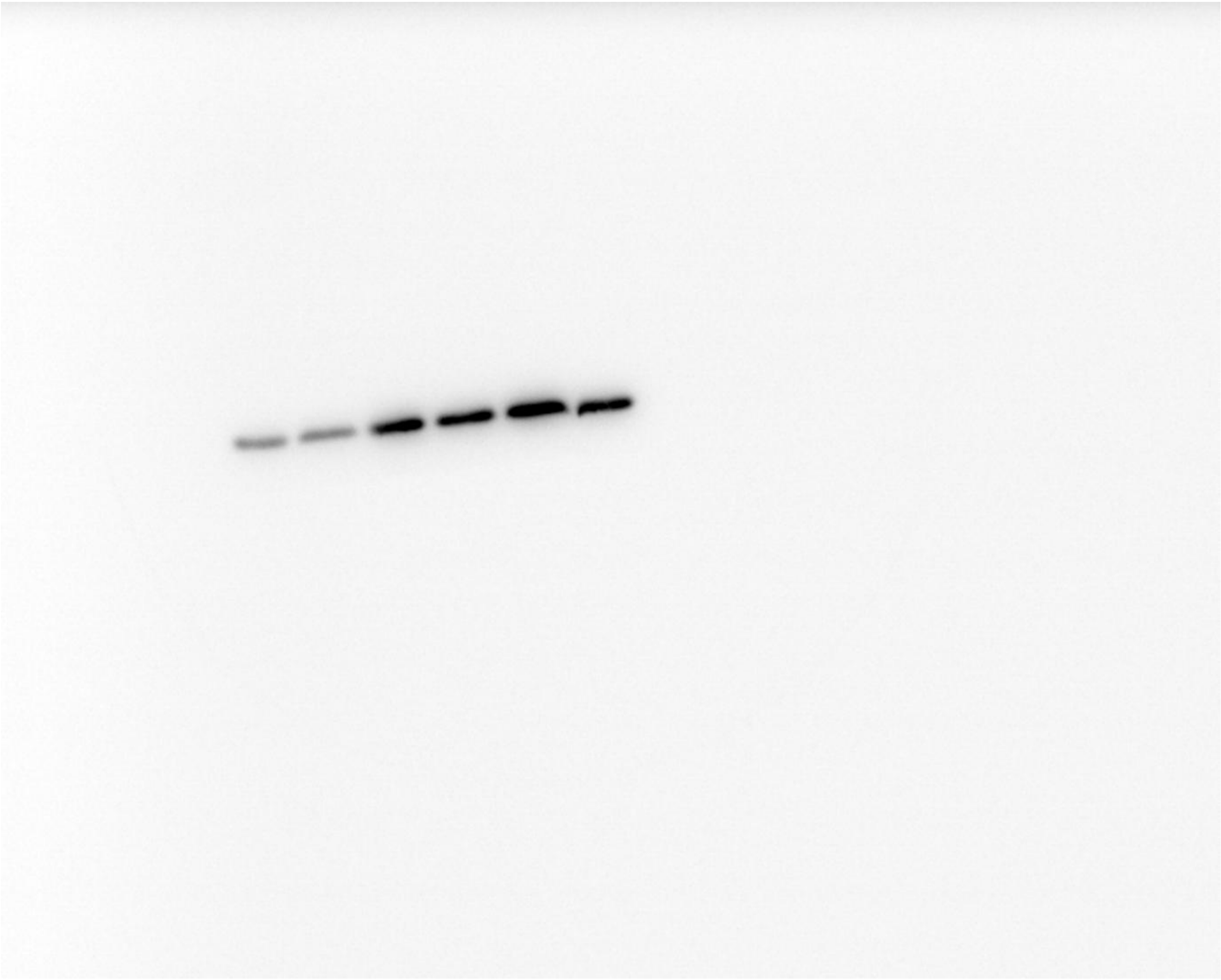

6E-MDA-MB-231-β-actin

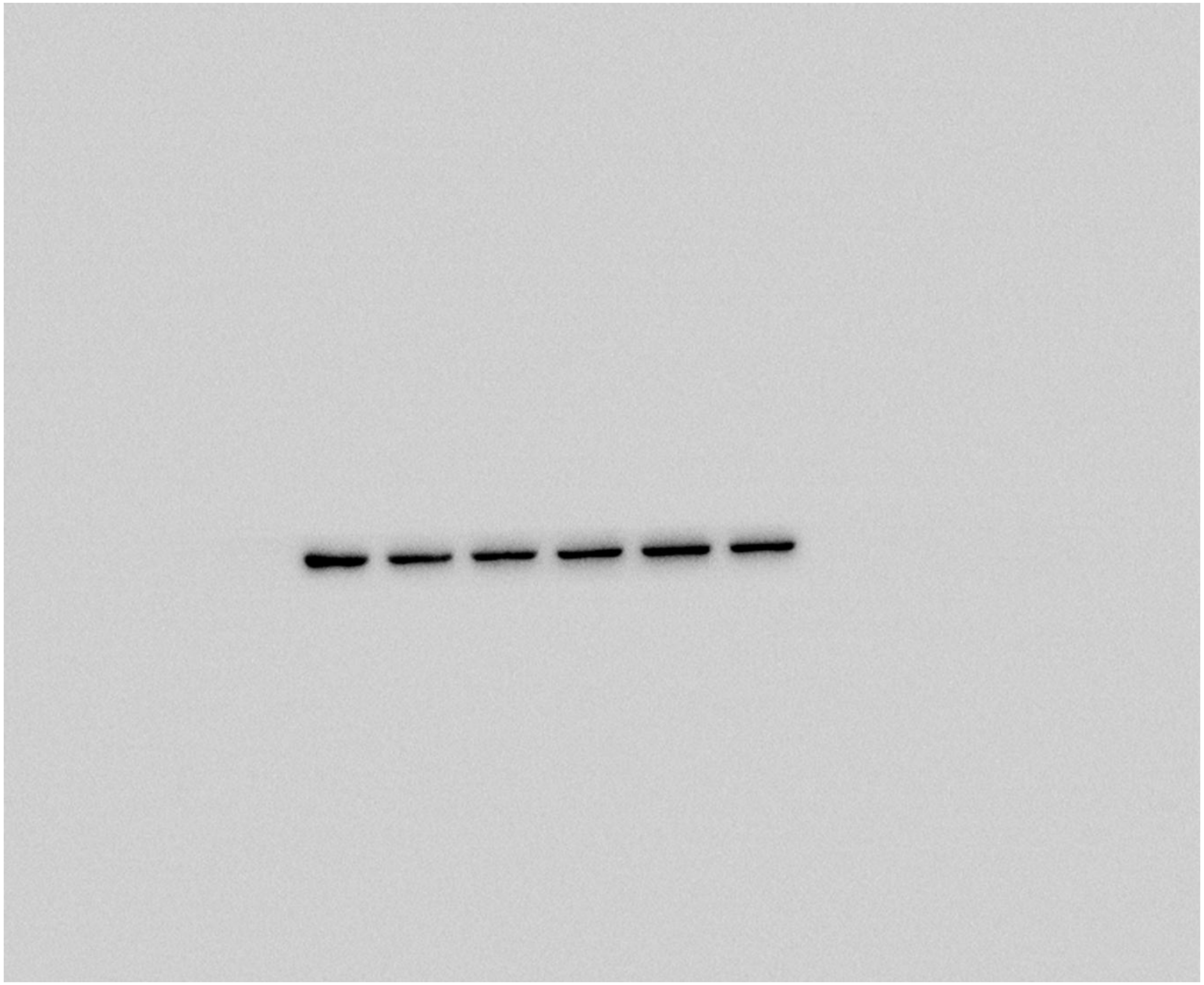

Supplement: Supplementary file 2 [file DataSheet1.PDF]
